# Dimerization of 9-Phenyl-ferroceno[2,3]indenylmethyl Radicals: Electrochemical and Spectroelectrochemical Studies

**DOI:** 10.1021/acsorginorgau.3c00070

**Published:** 2024-04-19

**Authors:** Larissa A. Casper, Katharina L. Deuter, Anja Rehse, Rainer F. Winter

**Affiliations:** Department of Chemistry, Universität Konstanz, Konstanz 78457, Germany

**Keywords:** fluorenylium, ferrocene, dynamic covalent chemistry, electrochemistry, spectroelectrochemistry, X-ray structure analysis

## Abstract

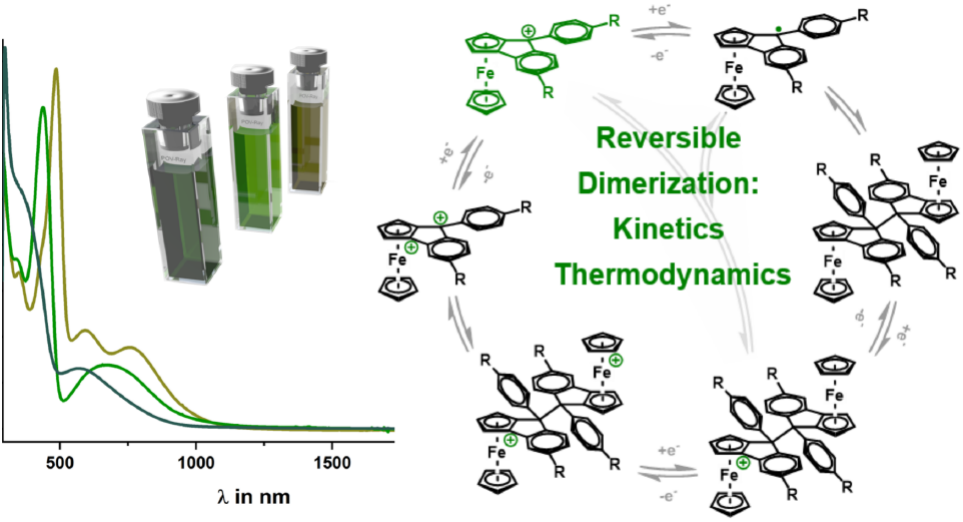

We report on three new 9-phenyl-substituted ferroceno[2,3]indenylmethylium dyes **1**^**+**^–**3**^**+**^ with electron-donating (OMe, Me) or electron-withdrawing (CF_3_) substituents. Complexes **1**^**+**^–**3**^**+**^ exist as racemic mixtures of *Rp* and *Sp* enantiomers. Pyramidalization at the methyl C atom in the precursor carbinol species **1-OH**–**3-OH** or the corresponding one-electron reduced radicals induces a second stereocenter, as the 9-phenyl substituent may reside in an *endo* or an *exo* position. Indeed, alcohol **2-OH** crystallizes as a racemate of *Rp*,*S* and *Sp*,*R* isomers. Cationic complexes **1**^**+**^–**3**^**+**^ are of deep green color and show intense electronic absorption in the visible. The oxidation and reduction processes are thoroughly investigated by means of cyclic voltammetry and UV/vis/NIR spectroelectrochemistry, the latter showing their electrochromic behavior. *T*-dependent EPR spectroscopy, EPR spin counting at 20 °C, as well as the UV/vis/NIR spectra of the reduced samples suggest that the one-electron reduced, neutral radicals dimerize nearly quantitatively (≥99.98%). Chemical reduction of **2**^**+**^ furnished an isomeric mixture of dimeric **2**–**2**. As was shown by cyclic voltammetry and UV/vis/NIR spectroelectrochemistry, the latter dimer redissociates to monomers **2**^**+**^ upon oxidation, thereby closing a reversible cycle of redox-induced C–C bond making and breaking.

## Introduction

**Scheme 1:**
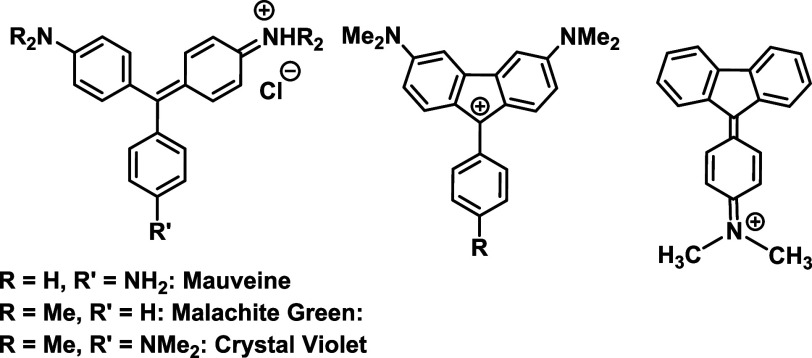
Tritylium and Related Fluorenylium Dyes and the Dominant Resonance Structure of the 4-NMe_2_Ph-Substituted Congener^6,12^

In 1856, 18-year-old William Perkin accidentally made purple Mauveine, the first synthetic organic colorant, in his attempt to synthesize quinine.^1,2^ Perkin’s discovery paved the way for a plethora of synthetic organic dyes to be discovered in the following 168 years.^3−11^ Other famous examples of triarylmethylium (tritylium) dyes are Malachite green and crystal violet (cf. Scheme 1).^6^

Although less common as a structural motif, examples of fluorenylium-type triarylmethylium dyes, including the analogs of the aforementioned tritylium dyestuffs, are also known, as shown in Scheme 1.^12−16^ Fluorenylium ions are generally considered to be antiaromatic due to their 4π-electron cyclopentadienylium constituent. Their antiaromatic character is, however, attenuated by 9-substituents that are able to delocalize the positive charge onto the periphery.^17−21^ As an example, most chemists conclude today that the 9-phenylfluorenylium cation combines local aromaticity with peripheral antiaromaticity.^22^ This becomes particularly evident in the 9-(4-dimethylaminophenyl)-substituted fluorenylium cation, whose experimentally determined structure is strongly suggestive of a quinoidally distorted phenyl ring (Scheme 1, right), rendering the five-membered ring of fulvenic nature.^20^

**Scheme 2:**
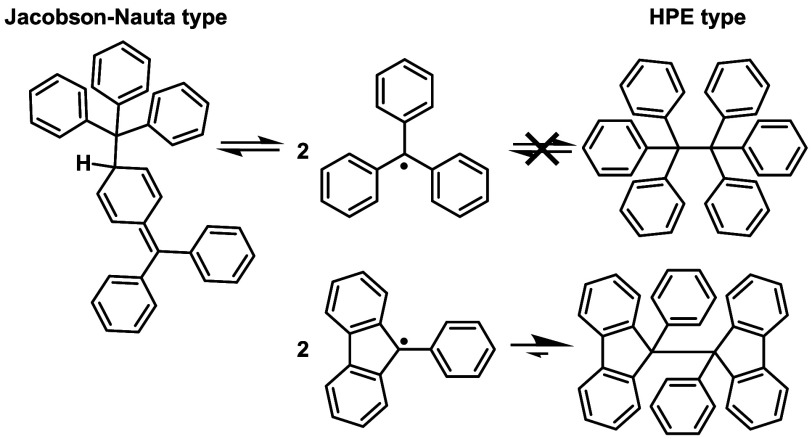
Dimerization of Trityl and Fluorenyl Radicals

One-electron reduced 9-phenylfluorenyl radicals are nonaromatic and possess nonplanar structures.^13,14,18,19,23−25^ They were shown to dimerize nearly quantitatively to hexaphenylethane (HPE)-type structures, hence displaying dynamic covalent chemistry.^16,26,27^ This mode of dimerization even pertains to the parent 9-phenylfluorenyl radical, which contrasts with the Jacobson–Nauta dimer formed by the trityl radical (cf. Scheme 2).^28−30^

**Scheme 3:**
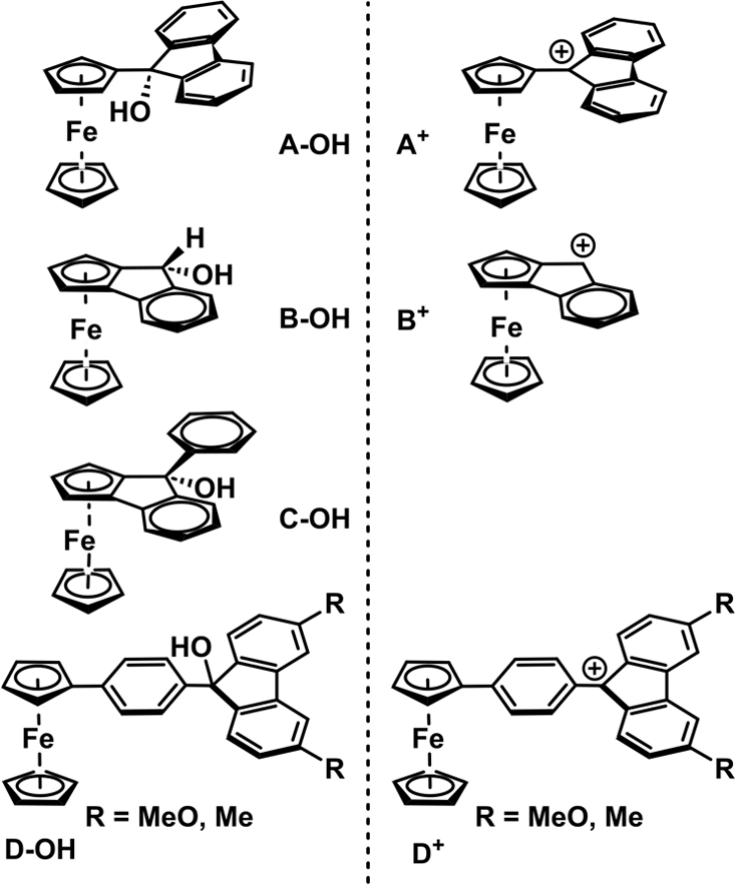
Ferrocenyl-Substituted Fluorenyl Carbinols (Left) and Derived Methylium Cations (Right) Reported in the Literature^34,36−38^

Our group has previously reported on some ferrocenyl-substituted tritylium dyes such as diferrocenylphenyl- and 4-phenylferrocenylmethylium complexes,^31−35^ including two 4-phenylferrocenyl fluorenylium congeners (**D**^**+**^ in Scheme 3).^34^ Earlier examples of ferrocenyl-modified fluorenylium cations are the green 9-ferrocenyl derivative **A**^**+**^**BF**_**4**_^**–**^ (Scheme 3) of Buchmeiser and Schottenberger, generated *in situ* from the ferrocene fluorenyl carbinol **A–OH** (cf. Scheme 3). However, only IR data were provided owing to its poor stability.^36^

**Scheme 4:**
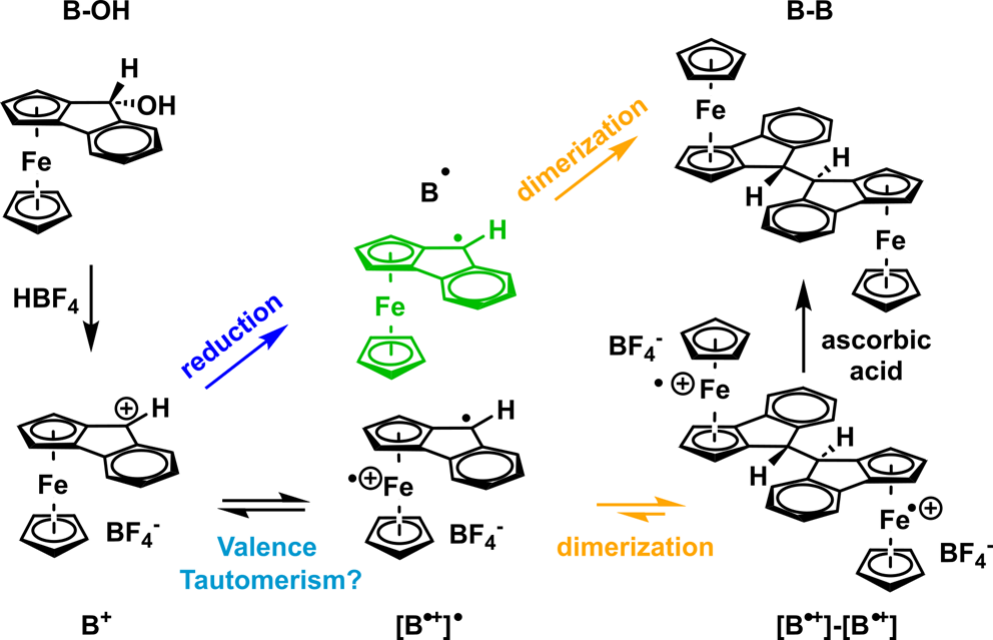
Dimerization of Ferroceno[2,3]indenylium Cation B^+^ via Its Valence Tautomer **[B**^•**+**^**]**^•^ and of the Corresponding Neutral Radical **B**^•^(42,43)

Only a handful of other *pseudo*-fluorenyl-type ferrocenes have been reported to date,^38^ mostly as their carbinols (e.g., **C–OH** in Scheme 3).^37^ Compound **B–OH** was first synthesized by reduction of the corresponding 2,3-ferrocenoindenone as early as 1965.^37,39−41^ As shown in Scheme 4, the deep green cation **B**^**+**^ was found to dimerize, most likely via a ferrocenium-fluorenyl diradical species **[B**^•**+**^**]**^•^, which is in essence a valence tautomer of fluorenylium ion **B**^**+**^.^42^ The resulting dicationic C–C coupled dimer **[B**^•**+**^**]–[B**^•**+**^**]** was then reduced by ascorbic acid to provide neutral **B–B**. Prior to that, Bildstein et al. had observed the dimerization of the one-electron reduced form **B**^•^ (cf. Scheme 4) of **B**^**+**^ and established its structure by X-ray crystallography.^43^

In our quest for new ferrocene-modified tritylium ions capable of exhibiting valence tautomerism, i.e., to coexist with their ferrocenium-trityl diradical isomers, we hence turned our attention to 9-phenyl-substituted ferroceno[2,3]indenyl methylium dyes akin to **B**^**+**^. To these ends, we synthesized and investigated three new congeners with electron-donating methoxy and methyl or electron-withdrawing trifluoromethyl substituents at the phenyl rings. Particular emphasis of the present work is on the optical and redox properties, which had so far remained elusive owing to the low stability and the high reactivity exhibited by **B**^**+**^ (Scheme 4). We have also determined the extent of dimerization of their monoreduced forms by quantitative EPR spin counting. The purposeful synthesis and electrochemical as well as spectroelectrochemical analysis of dimer **2**–**2** yielded further details on the chemically reversible cycle of an electron-transfer-induced formation and scission of a C–C bond.

## Synthesis and Characterization

**Scheme 5:**
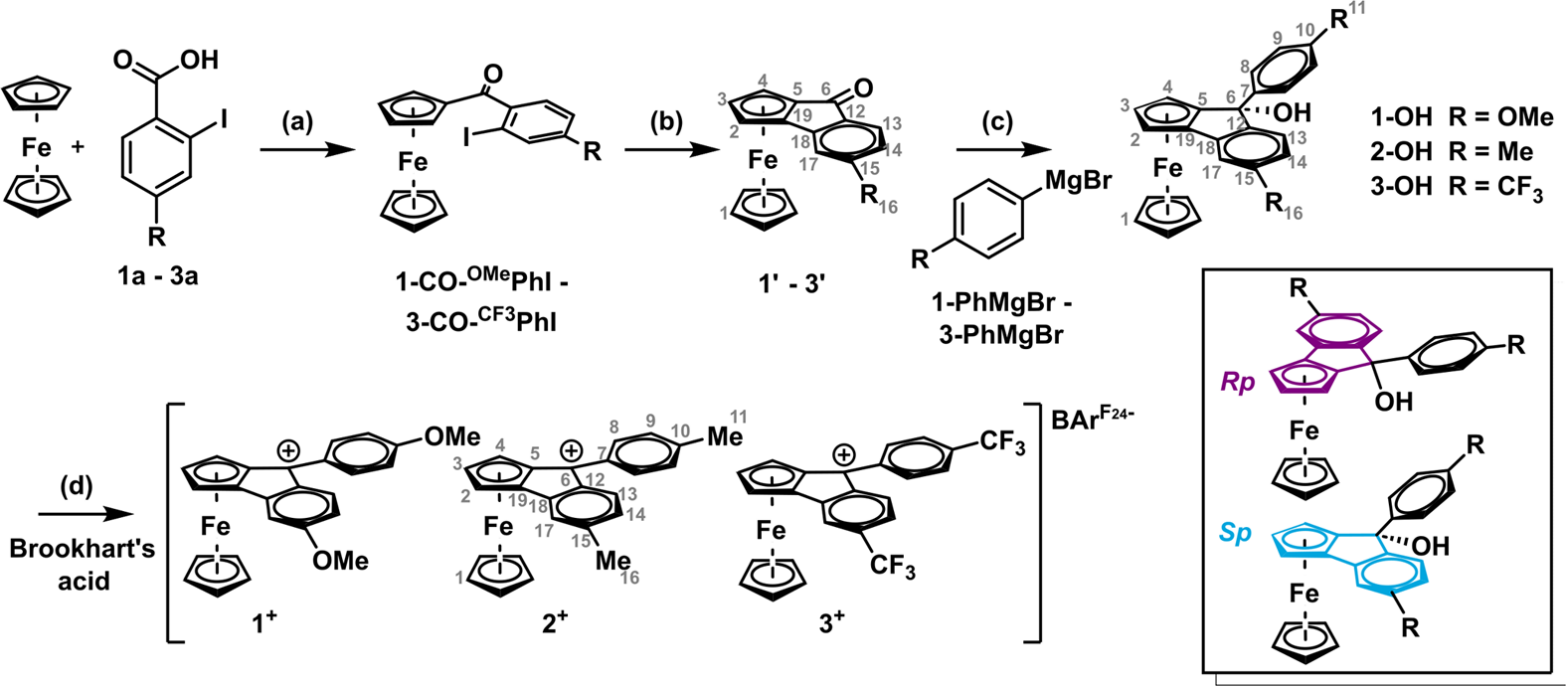
Synthesis of the New 9-Phenyl-ferroceno[2,3]indenylmethylium Cations **1**^**+**^–**3**^**+**^ and Their Carbinol Precursors **1-OH**–**3-OH** (a) C_2_Cl_2_O_2_, DMF, r.t., 1 h; then AlCl_3_, ferrocene, CH_2_Cl_2_, r.t., 16 h; (b) Pd(OAc)_2_, (*rac*)-BINAP, Cs_2_CO_3_, toluene, 100 °C, 20 h; (c) Mg, the corresponding ^R^PhBr, 1,2-C_2_H_4_Br_2_, Et_2_O for **2-OH** and **3-OH**, THF for **1-OH**, reflux, 1–4 h, then r.t., 48 h for **1-OH**; (d) Brookhart’s acid [H(OEt_2_)_2_]^+^ [B{C_6_H_3_(CF_3_)_2_-3,5}_4_]^−^ in CH_2_Cl_2_ or 1,2-C_2_H_4_Cl_2_, respectively, r.t., 5 min.

Complexes **1**^**+**^–**3**^**+**^ were synthesized by the four-step procedure shown in Scheme 5 according to the following sequence: (a) Friedel–Crafts acylation of ferrocene to provide ketones **1-CO**^**Me**^**-PhI**–**3-CO**^**CF3**^**-PhI**; (b) palladium-catalyzed asymmetric cross-coupling to ferroceno[2,3]-inden-1-ones **1′**–**3′**; (c) nucleophilic addition of the Grignard reagents **1-PhMgBr**–**3-PhMgBr**; and (d) protolytic dehydration of the resulting carbinols **1-OH**–**3-OH** with Brookhart’s acid [H(OEt_2_)_2_]^+^ [B{C_6_H_3_(CF_3_)_2_-3,5}_4_]^−^ ().^44^

Details of the syntheses of the benzoyl-substituted ferrocenes and of compounds **1′**–**3′** are provided in the Experimental Section of the Supporting Information. Because the BINAP diphosphine ligand in reaction (b) was used as a racemic mixture of *R* and *S* enantiomers, the obtained dark red to purple ferroceno[2,3]-inden-1-ones **1’**–**3′** are likewise 1:1 mixtures of *Rp* and *Sp* enantiomers (see Figure S29; for NMR data, see the Experimental Section in the Supporting Information and Figures S15–S27). As was reported by Le Plouzennec and Dabard,^37^ the Grignard reaction (c) to carbinols **1-OH**–**3-OH** proceeds with excellent stereoselectivity, with the aryl nucleophile approaching the fluorenone carbonyl substituent from the face opposite to the ferrocenyl residue. This results in an *endo* configuration, where the −OH substituent is poised toward the ferrocenyl unit (cf. Figure S29). As a consequence, carbinols **1-OH**–**3-OH** are obtained as racemic pairs of *Rp*/*S* and *Sp*/*R* enantiomers, which cannot be distinguished by their NMR spectra (for NMR and mass spectra, see Figures S30–S42).

In the case of ditolyl derivative **2-OH**, we succeeded in growing single crystals that proved suitable for X-ray crystallography. Details of the data collection, the crystal and structure data, as well as a comparison of the most pertinent bond lengths, interatomic bond angles and interplanar angles with related congeners can be found as Tables S1 and S2. **2-OH** crystallizes in the monoclinic space group *P*12_1_/*c*1. The four molecules within the unit cell constitute racemic pairs of two crystallographically independent individuals with slight structural differences between them.

**Figure 1:**
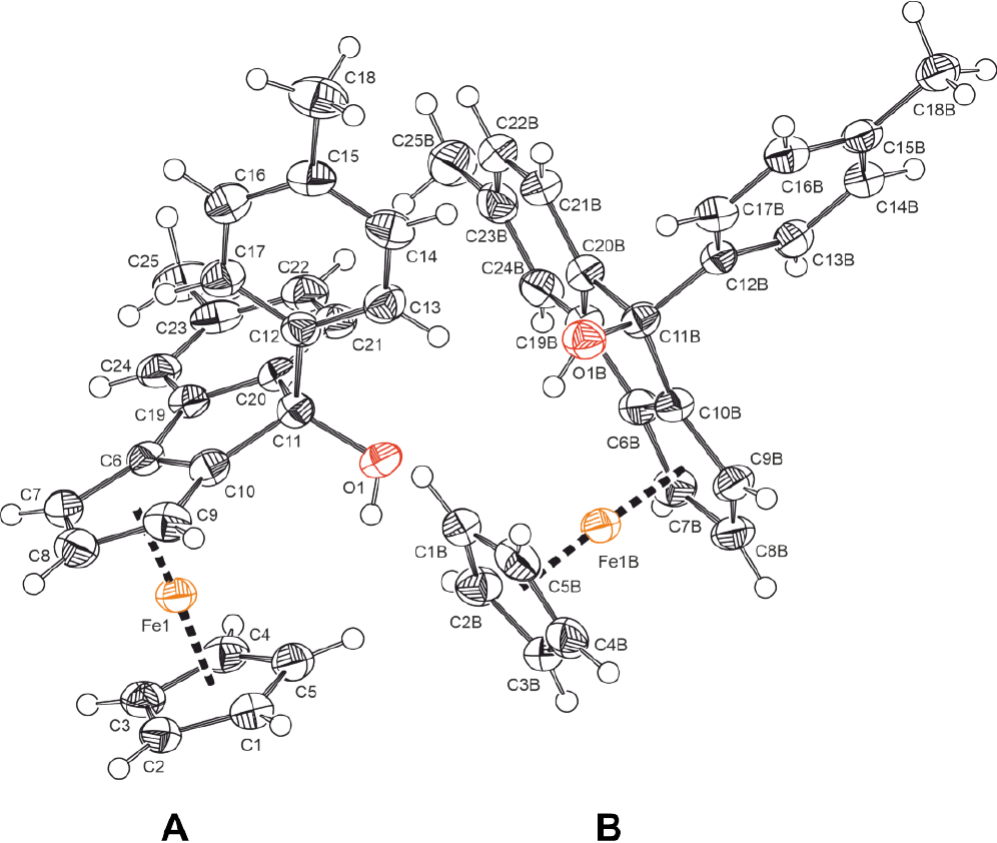
ORTEPs of one enantiomer (*Rp*, left; *Sp*, right) of each of the two crystallographically independent molecules in the unit cell of carbinol **2-OH** with the atomic numbering. Ellipsoids are displayed at the 50% probability level.

The two members of each pair represent the *Rp* and *Sp* enantiomers with respect to the chiral plane defined by the unsymmetrically 1,2-disubstituted cyclopentadienide (Cp) ring, whereas the two crystallographically distinct molecules differ mainly with respect to the mutual rotation of their Cp ligands. Both adopt an *endo* configuration at the chiral C atom C11(B). Figure 1 displays the molecular structures of one specific enantiomer for each of the two crystallographically independent molecules with the atom numbering.

The cyclopentadienide ligands of molecule A (left in Figure 1) adopt a nearly eclipsed conformation (average rotation angle of 3.5°), while that of molecule B (right in Figure 1) is in between eclipsed and staggered (average rotation angle 25.2°, cf. Table S1). At 4.1(3)°, the Cp decks of the individual ferrocenyl substituents are slightly tilted. Tilt angles for other ferroceno[2,3]indenyl carbinols in the literature range from 0.86(16) to 6.00(8)° (cf. Figure S44 and Table S2).^42,45^ The aryl and cyclopentadienide rings of the tricyclic skeleton are nearly coplanar with angles of 0.6(2) or 6.9(2)° between their ring planes, whereas the appended *p*-tolyl substituent (Ph1, cf. Figure 1) is rotated by 84.1(2) or 81.8(2)° out of the plane of the substituted Cp ring. Acute intracyclic bond angles at the sp^3^-hybridized carbinol C atom C10(B)–C11(B)–C20(B) of 100.4(3)° are counterbalanced by the opening of the C–C–C angles C10(B)–C11(B)–C12(B) involving the appended tolyl substituent to 112.0(4) or 113.9(3)°. C–C bond lengths within the ferrocenyl, indolyl, and phenyl constituents are unexceptional.

In the unit cell of **2-OH**, the two pairs of enantiomers associate through a total of eight intermolecular C–H···π interactions to centrosymmetric tetramers (see Figure S45). Close contacts exist between a proton of the tolyl rings of their ferroceno[2,3]indenyl skeletons (*d* = 2.816 Å, interplanar angle = 89.52°), the unsubstituted Cp rings and the same skeletal tolyl rings (*d* = 2.765 Å, interplanar angle = 85.64°), the unsubstituted Cp rings (*d* = 2.770 Å, interplanar angle = 81.95°), as well as the 9-tolyl substituents and the unsubstituted Cp rings (*d* = 2.890 Å, interplanar angle = 81.36°). The latter are augmented by pairwise, weaker C–H···O contacts of 2.662 Å between a proton of the unsubstituted Cp ring and the hydroxyl groups. Neighboring tetramers are connected by likewise strong intermolecular interactions between the orthogonally disposed substituted Cp rings (*d* C–H···C = 2.670 and 2.779 Å, interplanar angle 89.88°) as well as the substituted Cp rings and the tolyl substituents (*d* C–H···C = 2.773 and 2.811 Å, interplanar angle 49.11°) of the two crystallographically unique molecules. The latter interactions link tetramers to double chains that run parallel to the *a*-axis of the unit cell. Figure S45 shows such a double chain of interconnected tetramers along with the relevant intermolecular contacts. Neighboring double chains interlock through the mutual placing of tolyl rings of one chain into the voids of its neighboring chains, yet without any short intermolecular contacts. In contrast to other ferrocenyl carbinols FcC(OH)Ar_2_,^42−43^ no intermolecular O–H···O hydrogen bonds are observed. Instead, short contacts of 2.985 and 3.091 Å between the hydroxyl proton and the Fe atom of the same molecule exist (see the top panel of Figure S45).

As mentioned above, treatment of carbinols **1-OH**–**3-OH** with Brookhart’s acid resulted in protolytic dehydration to furnish the green to teal ferroceno[2,3]indenylmethylium ions **1**^**+**^–**3**^**+**^ in essentially quantitative yields as their  salts. Color impressions of their dichloromethane solutions are provided in Figure S46. The cationic complexes were readily identified by their ESI-mass spectra (cf. Figure S47), their characteristic ^1^H, ^13^C, and ^19^F NMR resonance signals (cf. Figures S48–S62) and their electronic absorption spectra (cf. Figure 7). Owing to rehydration during the measurement, the mass spectra of the cationic species **2**^**+**^ and **3**^**+**^ inevitably contain ion peaks of their carbinol precursors **2-OH** and **3-OH**. On the contrary, the mass spectrum of carbinol **1-OH** almost exclusively shows mass peaks assignable to **1**^**+**^ (cf. Figure S43). It appears that the ratio of the molecular ion peak of the respective cationic complex to that of its parent carbinol reflects the increasing stability of the cationic complex in the ordering **3**^**+**^ < **2**^**+**^ < **1**^**+**^.

**Figure 2:**
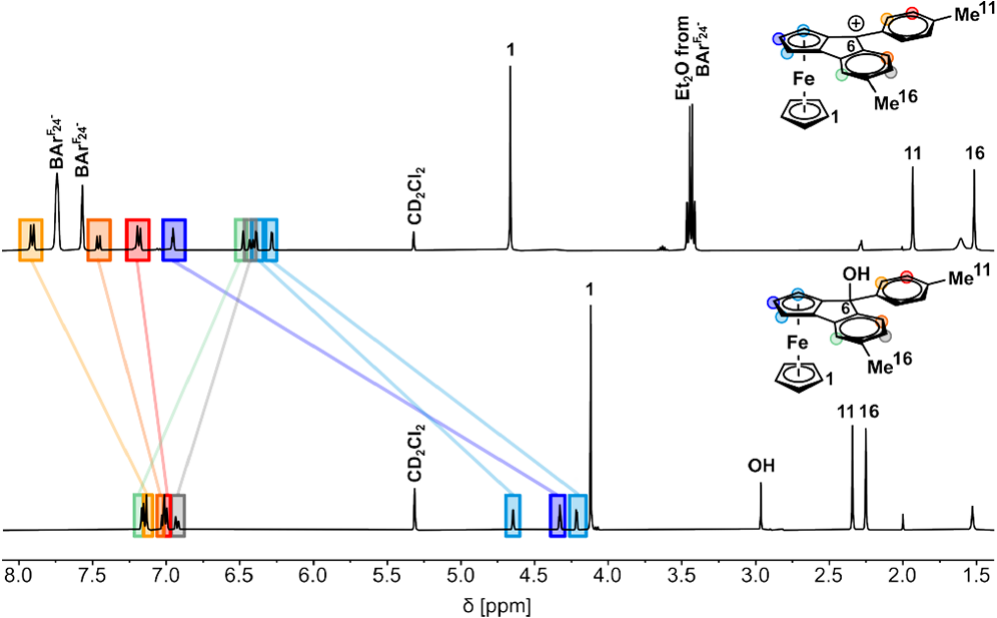
Comparison between the ^1^H NMR (400 MHz) spectra of carbinol **2-OH** (bottom) and the *in situ* generated cationic complex **2**^**+**^ (top).

Table 1 lists selected NMR data for the ketones, carbinols, and the ferroceno[2,3]indenylmethylium ions; the corresponding atom numbering is shown in Scheme 5. As a representative example of the spectral changes concomitant with cation formation, Figure 2 compares the ^1^H NMR spectrum of **2**^**+**^ to that of carbinol **2-OH**. The absence of any NMR resonance signals assignable to **2-OH** in the spectrum of **2**^**+**^ and the absence of other resonances indicate completeness of the conversion. Of note are the distinct downfield shifts of nearly all proton and carbon resonances within the cationic complexes. In concert with previous observations,^31^ the protons of the annulated Cp rings experience particularly large shifts of 1.96 (H-3 in **1**^**+**^) to 2.59 ppm (H-3 in **3**^**+**^) so that they show up well in the aromatic region (cf. blue marked signals in Figure 2). The only exception are the resonances of H-14 and H-17 at the substituted phenyl ring of the fluorenylium-type moiety, marked in gray and green colors in Figure 2, which are shifted upfield. In passing, we note that shift differences Δδ between the present ferroceno[2,3]indenylmethylium ions and their carbinol precursors are notably larger than those for ferrocenyl methylium cations FcCAr_2_^+^ and their parent carbinols.^31−33,35^

**Table 1:** Characteristic NMR Data for Ketones **1′**–**3′**, Carbinols **1-OH**–**3-OH**, and Cations **1**^**+**^–**3**^**+**^; Chemical Shifts δ in ppm

	H-17/C-17	H-13/C-13	C-6	H-3/C-3	H-2/H-4
					C-2/C-4
**1′**	6.71/107.4	7.44/124.8	194.2	4.79/74.9	4.93/4.82
					66.3/66.1
**2′**	7.00/123.0	7.38/121.4	195.3	4.83/75.2	4.83/4.95
					66.3/66.2
**3′**	7.39/116.9	7.58/123.2	193.6	5.08/76.4	4.98/4.97
					67.3/67.1
**1-OH**	6.91/106.8	7.06/125.4	79.1	4.34/70.9	4.66/4.25
					62.4/60.7
**2-OH**	7.21/121.5	7.06/124.5	79.5	4.37/70.8	4.65/4.22
					62.3/60.7
**3-OH**	7.62/117.5	7.40/125.5	79.3	4.46/72.0	4.80/4.31
					63.0/61.7
**1**^**+**^	6.41/111.1	7.53/128.1	137.4	6.75/91.5	6.16/6.16
					76.9/79.2
**2**^**+**^	6.47/126.4	7.46/123.7	150.8	6.95/95.2	6.39/6.28
					77.1/84.4
**3**^**+**^	6.84/121.8	7.63/121.0	156.7	7.05/99.3	6.63/6.48
					77.5/88.1

The resonance shift of carbon atom C-6, which changes from a tetrahedral, neutral trityl carbinol to a trivalent methylium center, is particularly sensitive toward substituent effects.^46−48^ It was found to shift by 58.3 ppm (vs **1-OH**) to 137.4 ppm in **1**^**+**^, by 71.3 ppm (vs **2-OH**) to 150.8 ppm in **2**^**+**^, and by 77.4 ppm (vs **3-OH**) to 156.7 ppm in **3**^**+**^. The ordering of δ (C-6) as **1**^**+**^ < **2**^**+**^ < **3**^**+**^ reflects the decreasing ability of the *para*-substituents at the intracyclic and the 9-phenyl rings to stabilize the positive charge at the methylium carbon atom^31,47^ and correspond to their Hammett parameters (σ*_p_*_-MeO_ = −0.27, σ*_p_*_-Me_ = −0.17, σ*_p_*_-CF3_ = +0.53).^49^ The present resonance shifts are nevertheless significantly smaller than those of 202.7 ppm for the parent 9-phenyl fluorenylium cation,^50^ 204.1 ppm for its 4-pentafluorsulfanyl,^50^ or 215.6 ppm for the 2-*^t^*butyl derivative,^51^ and even that of 161.0 ppm for the 4-dimethylamino-substituted congener.^20^ This again underlines the stabilizing effect of the incorporated ferrocenyl unit. Accompanying downfield shifts of the proton and carbon resonances of the annealed ferrocenyl ring, whose magnitudes follow the same ordering as δ (C-6), are a token of the increasing burden on the ferrocenyl constituent.

**Scheme 6:**
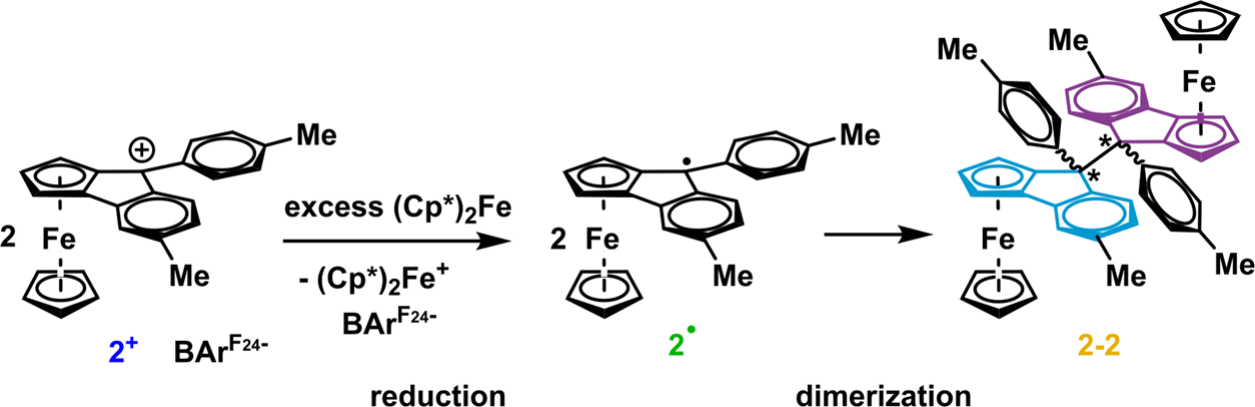
Synthesis of Dimer **2**–**2**

In expanding the scope of this work, we synthesized the racemic dimer **2**–**2** by reduction of **2**^**+**^ with an excess of decamethylferrocene as shown in Scheme 6. The neutral dimer was purified by gradient column chromatography and isolated as a light orange solid (cf. Experimental Section). As a token of successful reduction, decamethylferrocenium  was isolated as green crystals directly from the reaction mixture.

**Figure 3:**
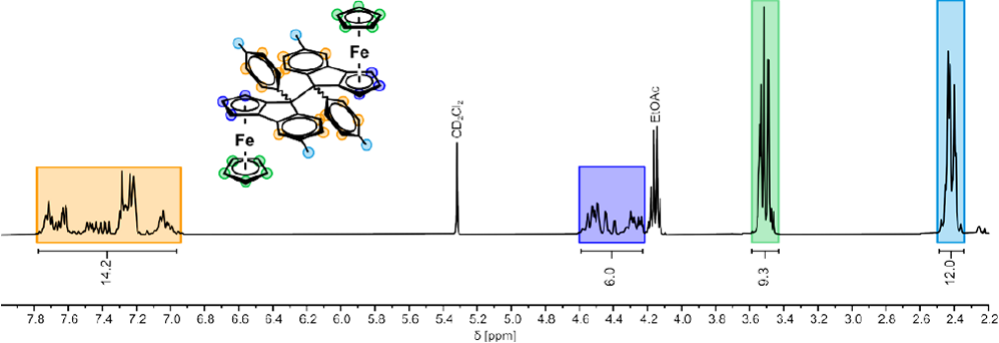
^1^H NMR (400 MHz) spectrum of the racemic mixture of potentially 10 different dimers **2**–**2** in CD_2_Cl_2_.

Radical intermediates **2**^•^ already exists as a racemic mixture of *Rp* and *Sp* enantiomers (see Figure S29). Dimerization produces two additional stereocenters at carbon atoms C-9/C-9′, as the 9-phenyl substituent may reside in an *endo*- or *exo*-position with respect to the annealed ferrocenyl nucleus. This leads to a total of 10 possible stereoisomers of the dimers, as is schematically shown in Figure S64. The resulting plethora of proton and carbon resonance signals (see Figures 3 and S63) thwarted proper characterization of **2**–**2** solely via NMR spectroscopy. However, integration of the shift regions of the aromatic protons (14H), the protons at the cyclopentadienide ligands (10 + 6H), and the methyl protons (12H) provided adequate integration ratios.

Further hints at the identity of **2**–**2** as such comes from the reasonable agreement between the experimental ATR-IR spectrum of the mixture with the DFT-calculated IR spectra of the two randomly chosen isomers 2 and 7 of Figure S65. ESI-MS only provided the mass peaks for **2**^**+**^ and the parent carbinol **2-OH** (cf. Figure S66), but no peaks that could be assigned to an ionized intact dimer. This points to ready dissociation of the latter upon oxidation, at least under the ionizing conditions of mass spectrometry (MS). The complete absence of **2-OH** in the ^1^H NMR spectrum of the dimer in Figure 3 indicates that the latter species originates from hydration of **2**^**+**^, which is formed during the MS experiment. Voltammetric and UV/vis/NIR spectroelectrochemical studies indeed confirmed that dimer **2**–**2** readily dissociates upon oxidation. This will be discussed later in this work.

## Electrochemistry

**Figure 4:**
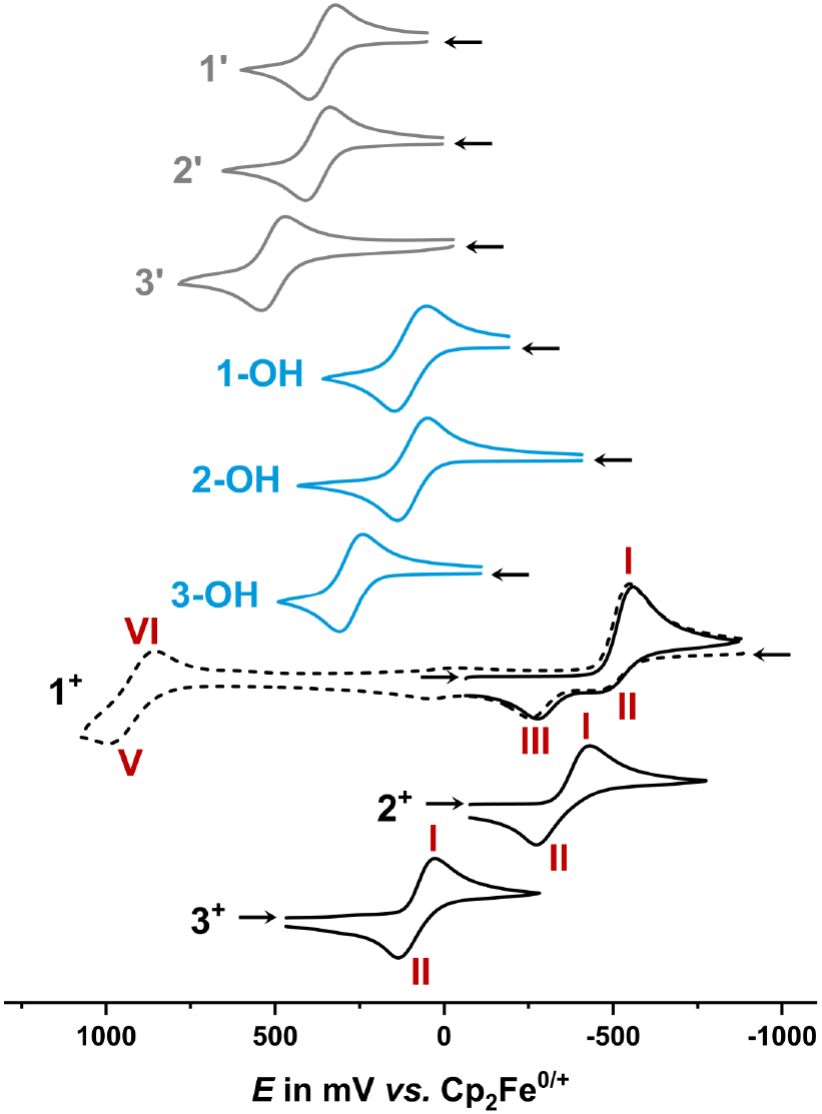
Cyclic voltammograms of **1′**–**3′**, **1-OH**–**3-OH**, and **1**^**+**^–**3**^**+**^ (CH_2_Cl_2_/ 0.1 M NBu_4_^+^, *T* = 293(±3) K, *v* = 100 mV/s, Pt working, Pt counter, and Ag/AgCl (pseudo) reference electrode). Plotting convention: Polarographic.

The redox properties of carbinols **1-OH**–**3-OH** and fluorenylium-type cations **1**^**+**^–**3**^**+**^, as well as those of their ketone precursors **1′**–**3′** were probed by cyclic voltammetry. For the sake of consistency, and with view of the high electrophilicity of fluorenylium cations,^20,47,48^ our electrochemical studies employed the very weakly nucleophilic 0.1 M NBu_4_^+^/CH_2_Cl_2_ electrolyte.^31,52^ Pertinent data are compiled in Figure 4 and Table 2.

**Table 2:** Electrochemical Data^a^ for All Complexes

	*E*_1/2_^0/+^ (Δ*E*_p_) [mV]	*i*_p,a_/*i*_p,c_	*E*_1/2_^+/0^ (Δ*E*_p_) [mV]	*i*_p,a_/*i*_p,c_
**1’**	+383 (73)	0.96	-	-
**2’**	+381 (72)	0.98	-	-
**3′**	+511 (64)	0.87	-	-
**1-OH**	+94 (96)	0.91	-	-
**2-OH**	+93 (88)	0.97	-	-
**3-OH**	+274 (66)	0.90	-	-
**1**^**+**^	+927 (103)^b^	-b	–508 (80)	0.35
**2**^**+**^	-	-	–353 (134)	0.88
**3^+^**	-	-	+82 (109)	0.97
**(9-Ph)FLU-C**^**+**^	-	-	+110^c^	-
**9-(2,4,6-Me_3_)PhFLU-C^+^**			+217^c^	

a Potentials (±3 mV) in mV in CH_2_Cl_2_/NBu_4_^+^ [BAr^F_24_^]^−^ (0.1 M) at *T* = 293(±3) K and at a scan rate *v* of 100 mV/s relative to the Cp_2_Fe^0/+^ redox couple.

b Different redox process: *E*_1/2_^+/2+^; peak current ratio could not be analyzed due to the proximity to the limit of the redox window.

c In DMSO/0.2 M NEt_4_^+^ BF_4_^–^.^51^

All ketones (gray in Figure 4) and carbinols (blue in Figure 4) exhibit a quasi-reversible one-electron oxidation to the corresponding ferrocenium species **1-OH**^**+**^–**3-OH**^**+**^ or **1′**^**+**^–**3′**^**+**^ with close to or somewhat larger peak-to-peak splittings or smaller peak current ratios *i*_p,c_/*i*_p,a_ than the ideal values of 59 mV or unity (see data in Table 2). Half-wave potentials of the carbinols range from 93 mV (**2-OH**) to 274 mV (**3-OH**) and are all positive of the *E*_1/2_ of 0 mV for the ferrocene/ferrocenium redox couple. Oxidation potentials of the ketones **1′**–**3′** (cf. Table 2) are shifted even further anodically, to 383–511 mV. Trends in the redox potentials of both types of ferroceno[2,3]indanes respond to the Hammett parameters σ*_p_* of their *para*-substituents (cf. Figure S67).

Conversion of the carbinols to the fluorenylium species induces a further, even more substantial anodic shift. Only for the most electron-rich methoxy-substituted cation **1**^**+**^ were we able to detect the ferrocene-based oxidation wave (peaks **V**/**VI** in Figure 4). The oxidations of **2**^**+**^ and **3**^**+**^ are evidently shifted to potentials larger than +1400 mV, which defines the anodic limit in these experiments. Attempts to measure the oxidation potentials via square wave voltammetry in liquid SO_2_ at −20 °C^32^ unfortunately failed due to decomposition of the cations in this solvent.

*E*_1/2_^+/0^ values for the ligand-centered reduction of the fluorenylium-type cations span a range of 590 mV and likewise align with the Hammett σ_p_ constants of the aryl substituent (cf. Figure S67). Comparison of **2**^**+**^ with the related 9-phenylfluorenylium ion **(9**-**Ph)-FLU-C**^**+**^ and its 9-mesityl derivative^53^ (see the entries in Table 2) shows that the annealed ferrocenyl donor shifts the reduction potential by roughly 450 mV to more negative values, although the accuracy of this comparison is somewhat compromised by the missing methyl substituent in **(9**-**Ph)-FLU-C**^**+**^ and the different solvents and electrolytes used.^33^ Larger than ideal peak potential splittings for the reduction/reoxidation process, in particular of complexes **2**^**+**^ and **3**^**+**^ (cf. Table 2), might be rooted in the accompanying structural change from a planar methylium cation with a sp^2^-hybridized carbon atom to a pyramidalized radical structure with an sp^3^ carbon center.

**Scheme 7:**
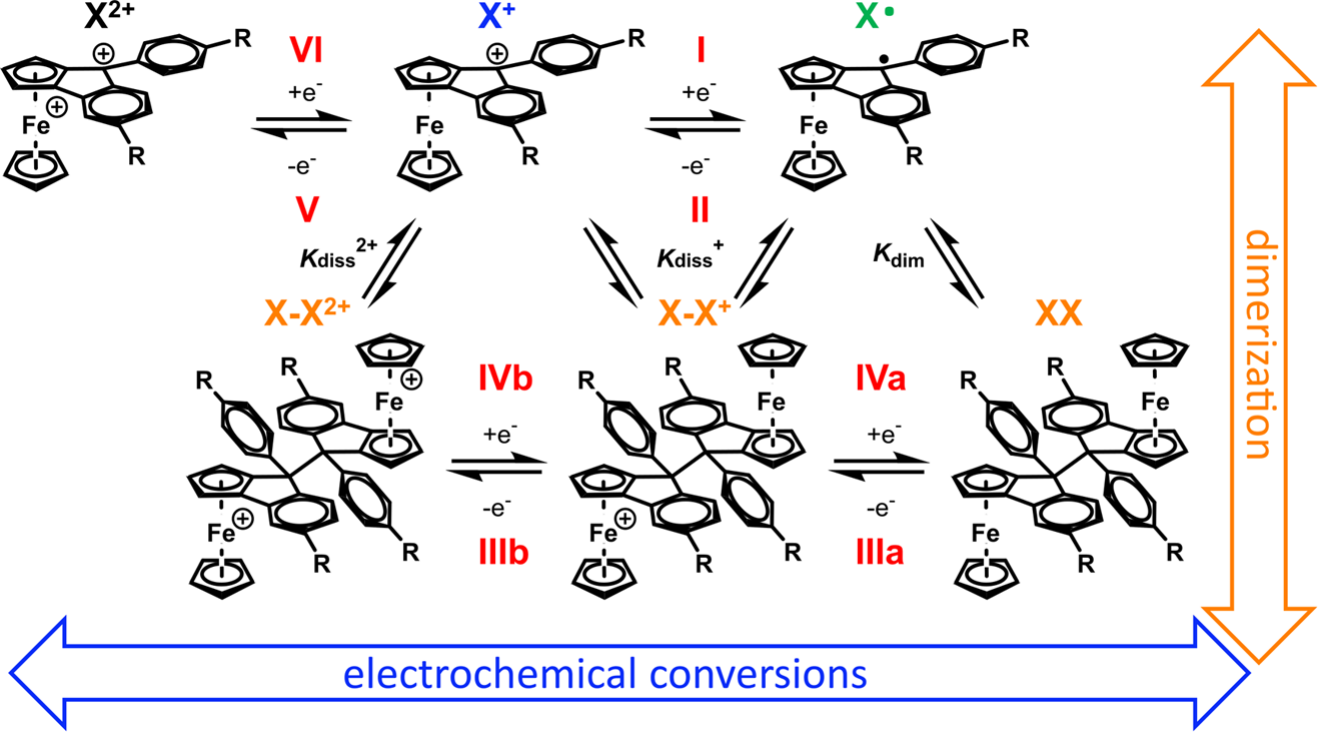
Electrochemical and Chemical Reactions of [X_*m*_]^*n*+^ in Solution (*m* = 1, *n* = +1, 0; *m* = 2: *n* = 0, + 1, + 2)

**Figure 5:**
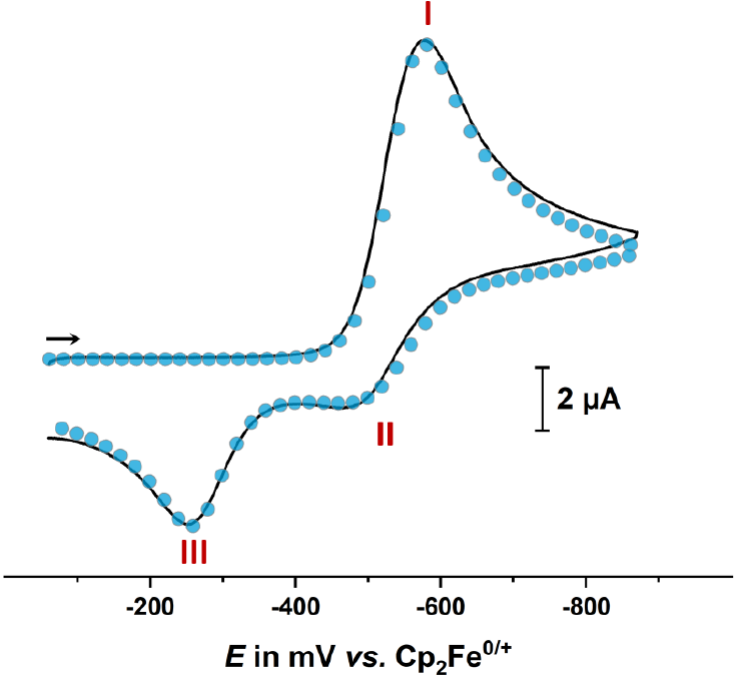
Experimental cyclic voltammogram of **1**^**+**^ (black line) at *v* = 400 mV/s and the corresponding simulation (blue dots) with the individual electrochemical and chemical steps of Scheme 7 denoted by red Roman numbers. Measured in CH_2_Cl_2_/0.1 M NBu_4_^+^ at *T* = 293(±3) K with a Pt working, Pt counter, and Ag/AgCl (pseudo) reference electrode). Plotting convention: Polarographic.

On comparing the voltammograms of the cationic complexes in Figure 4, one notes that the behavior of cation **1**^+^ deviates from that of its congeners in that the reduction constitutes a chemically irreversible process. At *v* = 0.1 V/s and at r.t., the peak current ratio *i*_p,a_/*i*_p,c_ (peaks **I** and **II**) is only 0.35. Moreover, in the anodic scan, after passing through wave **I**/**II**, an additional anodic peak marked as **III** appears, which is absent when the sweep is clipped before entering the reduction wave. Evidently, the ensuing radical **1**^•^ is chemically reactive—a likely scenario is dimerization, as it is often observed for fluorenyl radicals.^16,26,27^ Considering only the reductive part of the voltammograms associated with peaks **I**–**III**, we could successfully reproduce the shape of the experimental voltammograms by digital simulations based on the reaction diagram provided in Scheme 7. Figure 5 illustrates the excellent match at the example of an intermediate scan rate of 400 mV/s. The values of the equilibrium and rate constants of the dimerization of **1**^•^ and the dissociation of dimer [**1**–**1**]^*n*^^+^ (*n* = 1, 2) were optimized by using voltammograms recorded over a range of scan rates from 50 mV/s to 2 V/s (cf. Experimental Section for details). The estimated values of the equilibrium constant *K*_dim_ of 4 × 10^4^ L mol^–1^ and of the forward rate constant *k*_f_ of 1.7 × 10^4^ s^–1^ for the dimerization of **1**^•^ indicate that, at room temperature, the equilibrium lies nearly completely on the side of the dimer **1**–**1** and that dimerization is very fast.

Although only one reoxidation peak **III** is apparent in the reverse scan, two separate, consecutive one-electron steps **IIIa** and **IIIb** (cf. the bottom horizontal branch of Scheme 7) with close-lying oxidation potentials were required in order to reproduce the experimental voltammograms adequately. Equilibrium constants *K*_diss_^*n*+^ = (*K*_dim_^*n*+^)^−1^ for dissociation of the oxidized dimer [**1**–**1**]^*n*+^ increase strongly from the one- (*n* = 1, *K*_diss_^+^ = 0.2 mol L^–1^) to the two-electron oxidized form (*n* = 2, *K*_diss_^2+^ = 2 × 10^6^ mol L^–1^). These processes are shown as the vertical branches in Scheme 7. The large values of *K*_diss_^2+^ and the rate constant *k*_f,diss_ = 8 × 10^9^ s^–1^ for the dissociation of [**1**–**1**]^2+^ account for the absence of a rereduction wave for the oxidized dimer (i.e., the cathodic counterpeak of peak **III** in Figure 5), even after multiple cycles.

**Figure 6:**
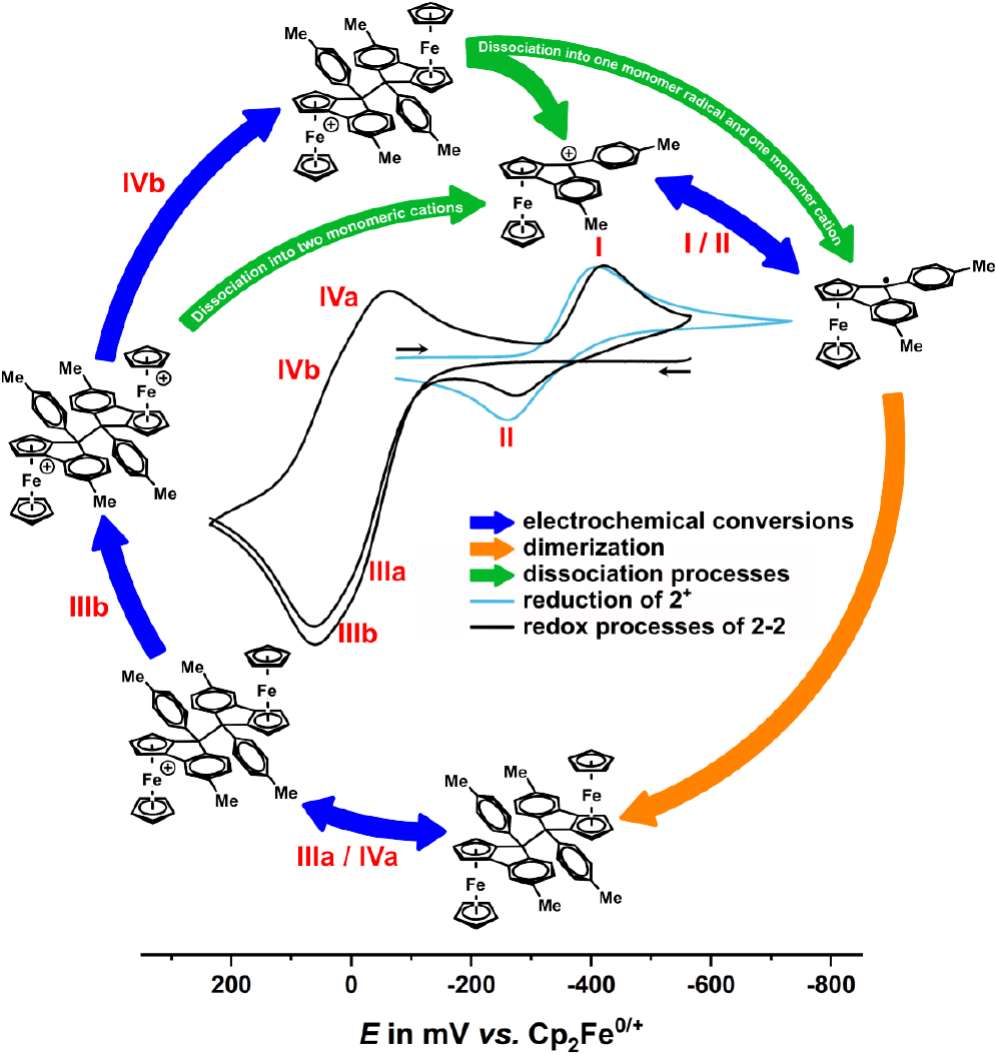
Cyclic voltammogram of dimer **2**–**2** (black) and cation **2**^**+**^ (light blue) integrated into the (electro-)chemical conversion (blue arrows) cycle, including the dimerization (orange arrow) of the monomer radical and the dissociation (green arrows) of the oxidized dimers. Partially reversible redox processes associated with **2**^**+**^ and **2**–**2** are indicated by blue double arrows.

The availability of **2**–**2** allowed us to also probe the monomer/dimer equilibrium from the side of a neutral dimer. As shown in Figure 6, voltammograms of the mixture of stereoisomers of dimer **2**–**2**, generated by reduction of **2**^**+**^ (*vide supra*), show two partially reversible, narrowly spaced one-electron oxidations denoted as peaks **IIIa**/**IIIb** with associated cathodic counterpeaks **IVb**/**IVa**. The presence of rereduction peaks **IVb** and **IVa** indicates that dissociation of [**2**–**2**]**^+^** into **2**^**+**^ and **2**^•^, or of [**2**–**2**]^2+^ into two equivalents of **2**^**+**^, occur at a slower rate when compared to **1**–**1**, where dissociation of the oxidized dimer [**1**–**1**]^2+^ was too fast to allow for the observation of associated counterpeaks. When, after scanning through the anodic wave of **2**–**2**, the cathodic scan is continued to more negative potentials, a cathodic follow peak **I** and its associated anodic return peak **II** appear. Peaks **I** and **II** match with those of the authentic **2**^**+**^**/2**^•^ redox couple recorded under the same conditions, as shown by the light blue curve in Figure 6. The suggested mechanism is detailed in Figure 6, so as to show the relevant species at any potential of the voltammetric scan of dimer **2**–**2**. In further contrast to **1**^•^, dimerization of **2**^•^ occurs on a slower time scale than the CV experiment so that peaks **IIIa**/**IIIb** are missing when the anodic scan following reduction of **2**^**+**^ is continued to potentials positive of the **IIIa,b**/**IVa,b** redox couples (cf. Figure S68). The observation of wave **I**/**II** of **2**^**+**^ after prior oxidation of dimer **2**–**2** confirms the proposed dimerization/dissociation pathways and the chemical identity of dimer **2**–**2**. It also attests to full chemical reversibility of the entire reaction cycles shown in Scheme 7 and Figure 6.

## Spectro(electro-)chemistry

**Figure 7:**
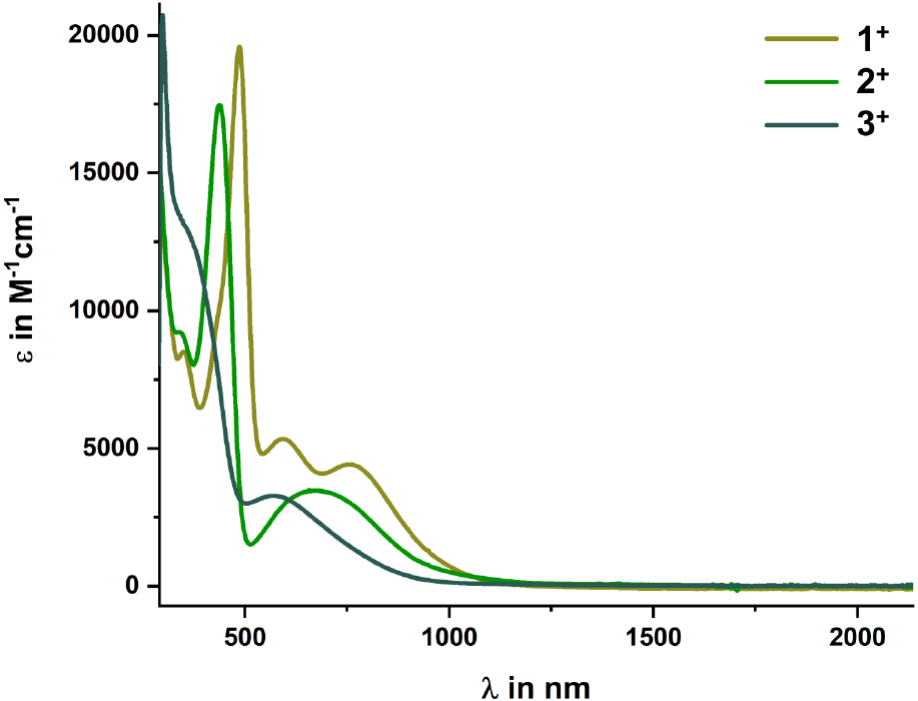
UV/vis/NIR spectra of the cationic complexes **1**^**+**^–**3**^**+**^.

Complexes **1**^**+**^–**3**^**+**^ are intensively colored, providing green ocher solutions for **1**^**+**^, grass green ones for **2**^**+**^, and fir green solutions for **3**^**+**^ (see Figure S46). They concomitantly absorb at low energy in the visible regime of the electronic spectrum. The UV/vis/NIR absorption spectra of the complexes are compared in Figure 7; pertinent data are compiled in Table 3.

**Table 3:** UV/Vis/NIR Data of the Cationic Complexes and Computed HOMO/LUMO Energies^a^

	λ [nm] (ε [×10^3^ L mol^–1^ cm^–1^])^b^	*E*_HOMO_/*E*_LUMO_^c^ [eV]	*E*_LUMO_–*E*_HOMO_/*E* of the *y*_M_ band^c^ [eV]	∠_C4–C7_/∠_C5–C8_
**1**^**2+**^	351 (7.5), 547 (15.3), 596 (9.3), 757 (1.2)	-	n.a.	-
**1**^**+**^	347 (8.9), 429 (9.6), 486 (19.7), 594 (5.7), 761 (4.4)	–6.696/–3.912	2.784/1.629	–1.0°/–29.3°
**1**^•^/**1**–**1**	347 (2.1), 486 (0.3), 596 (0.1)	-	n.a.	-
**2**^**2+**^	359 (5.2), 429 (4.0), 617 (0.7)	-	n.a.	-
**2**^**+**^	339 (4.5), 437 (8.4), 668 (1.4)	–6.883/–4.124	2.759/1.746	–4.5°/–31.7°
**2**^•^/**2**–**2**	339 (1.4), 437 (0.3)	-	n.a.	-
**3**^**2+**^	310 (17.0), 358 (13.6), 573 (1.1)	-	n.a.	-
**3**^**+**^	371 (15.1), 574 (3.5)	–7.289/–4.499	2.790/2.140	–11.5°/–36.6°
**3**^•^/**3**–**3**	359 (3.0), 466 (0.7)	-	n.a.	-

a In 1,2-C_2_H_4_Cl_2_/0.1 M NBu_4_^+^ at *T* = 293(±3) K.

b Absorption coefficients ±100 L mol^–1^ cm^–1^.

c Energies in eV from DFT calculations with the PBE0 functional.

**Figure 8:**
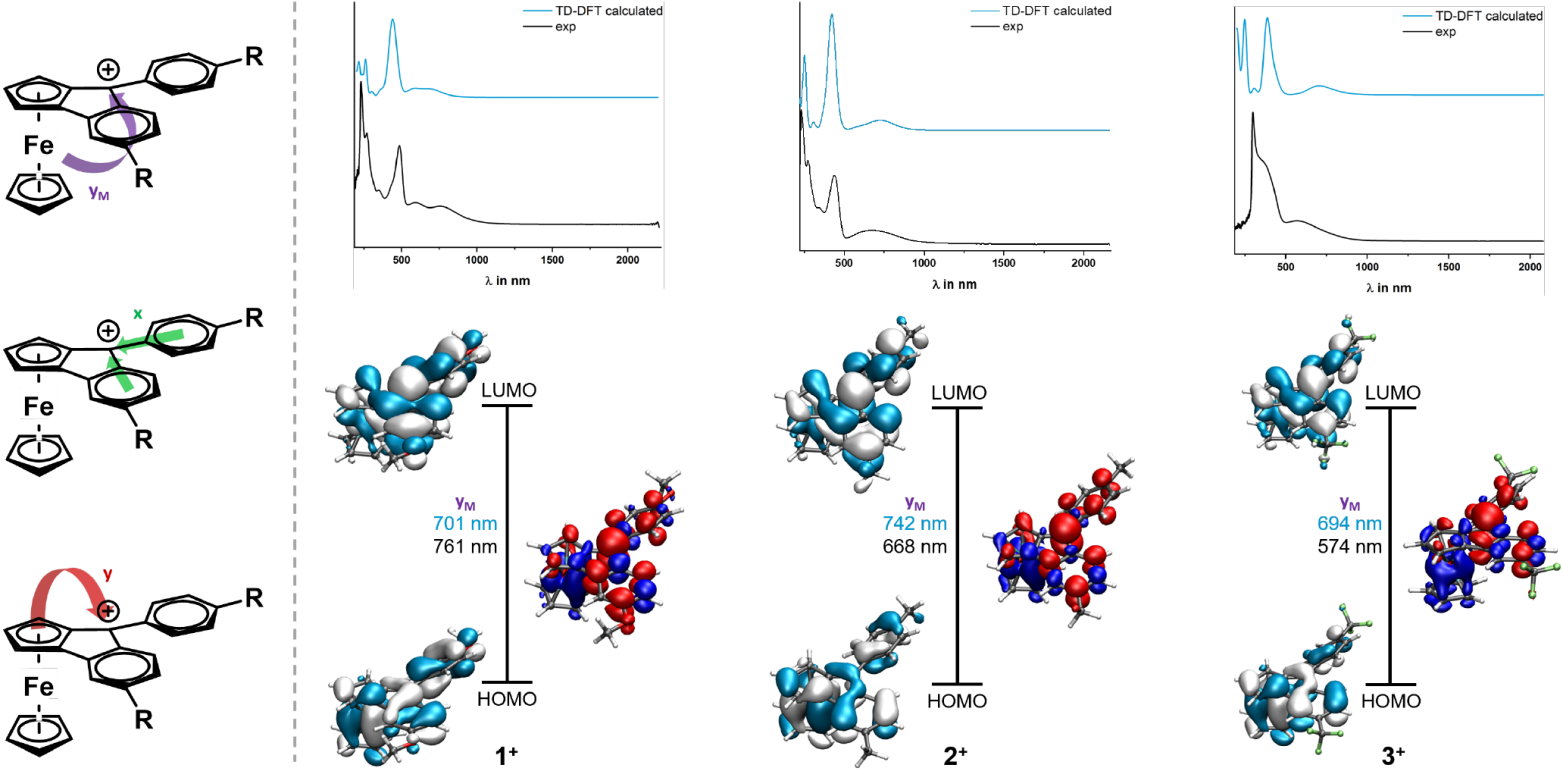
Top: TD-DFT calculated UV/vis/NIR spectra (blue lines) and experimental electronic spectra of **1**^**+**^–**3**^**+**^ (black lines). Bottom: MOs involved in the major transitions and associated electron density difference maps (electron density loss in blue, electron density gain in red color) for the ferrocene-to-fluorenylium charge transfer excitation (the so-called y_M_-transition). The left panel symbolizes the direction of electron flow during the individual electronic transitions.

UV absorptions at λ < 340 nm correspond to π–π* transitions that involve the entire tritylium-type framework, including the annealed Cp deck. The prominent electronic absorptions in the near-UV or the visible region were readily identified by TD-DFT calculations as the so-called x and y bands of tritylium dyes as defined by Duxbury (cf. Figure S69).^54^ They target the lowest unoccupied molecular orbital (LUMO), which is delocalized over the integrated Cp deck and the conjoint aryl rings with a large MO coefficient at the methylium carbon atom. The respective donors are the Cp deck of the ferrocenyl unit for the y-band, and the two phenyl rings for the x-band. Contour plots of the molecular orbitals (MOs) that contribute to the individual transitions of **1**^**+**^–**3**^**+**^ and the respective TD-DFT computed electron density difference maps (EDDMs) are displayed in Figure 8, along with the direction of electron flow concomitant with the different absorption bands (left panel). Figure S69 provides a full overview over all relevant transitions for complex **2**^**+**^.

In agreement with other ferrocenyl-substituted triarylmethylium dyes,^54^ the spectra of **1**^**+**^–**3**^**+**^ feature an additional metal-to-ligand charge transfer (MLCT) band (the so-called y_M_-band), which originates from the Fe-based HOMO and also targets the LUMO.^31−34^ As a consequence of ferrocene incorporation into a π-extended ferroceno[2,3]indenylmethylium chromophore, the HOMO is however not wholly confined to the ferrocenyl unit. The blue shift of the y_M_-band in the ordering **1**^**+**^ < **2**^**+**^ < **3**^**+**^ shows that the phenyl substituents influence the energy of the HOMO more than that of the LUMO (cf. Table 3). Perhaps surprisingly, extinction coefficients of the x-, y- and y_M_-bands are smaller than those of unconstrained Fc-C^+^(C_6_H_4_R)_2_ analogues.^31^ A likely reason is that incorporating the ferrocenyl donor into a rigid, planar molecular backbone attenuates the degree of charge transfer (CT) during these excitations.

**Figure 9:**
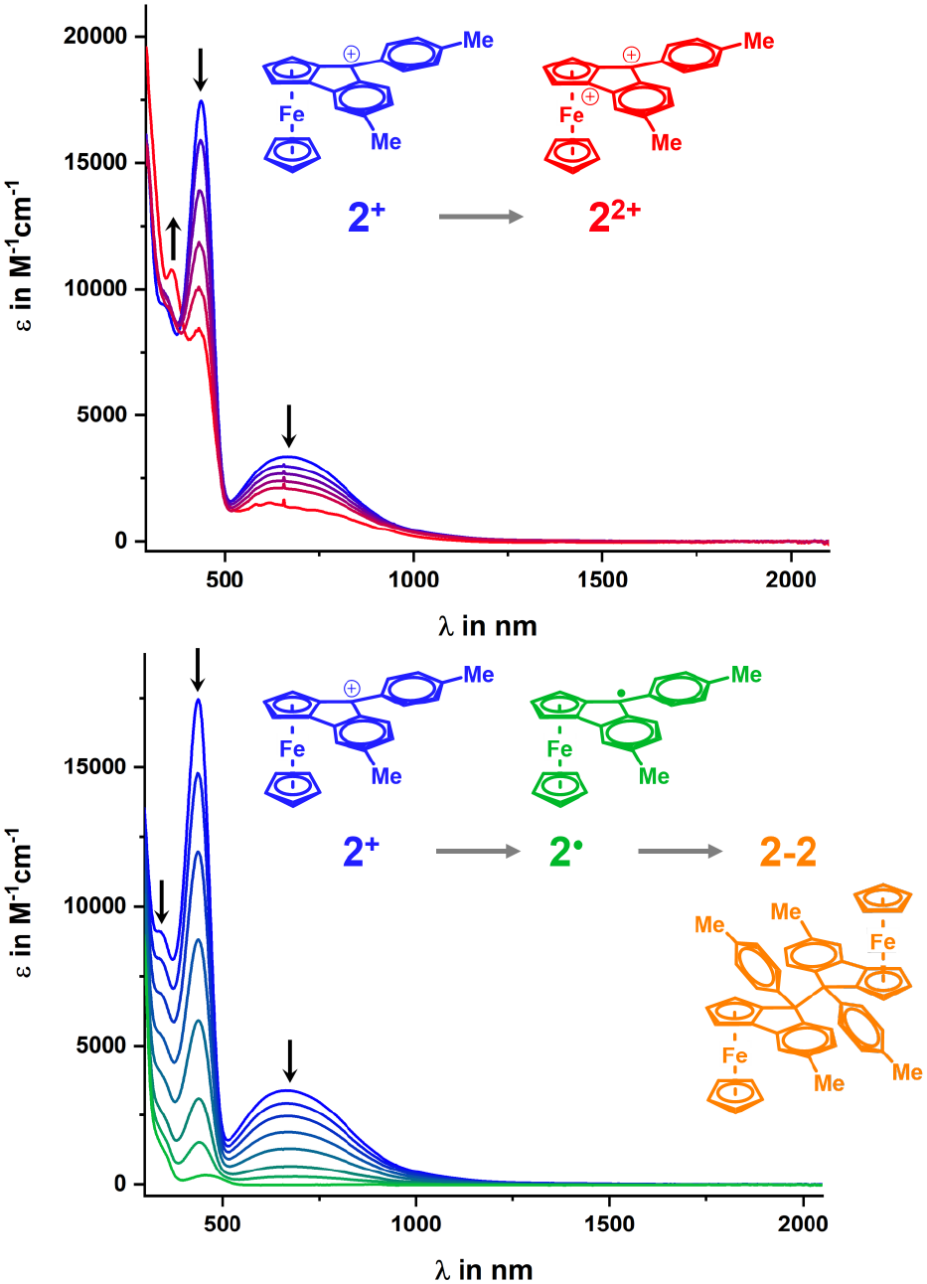
Spectroscopic changes during oxidation (top panel, blue to red curves) and reduction (bottom panel, blue to green curves) of **2**^**+**^ in 1,2-C_2_H_4_Cl_2_ with 0.1 M NBu_4_^+^ as the supporting electrolyte at *T* = 293(±3) K.

The CT character of all vis absorptions of complexes **1**^**+**^–**3**^**+**^ should render their electronic spectra susceptible to redox processes. We hence studied their electrochromic behavior by spectroelectrochemical methods. In these experiments, we were not only able to monitor spectroscopic changes concomitant with their reduction to neutral radicals **1**^•^–**3**^•^, but also those accompanying their oxidation to **1**^**2+**^–**3**^**2+**^. This was possible by applying high positive overpotentials in the electrolysis (cf. Experimental Section). However, the oxidation processes were only partially reversible with sometimes incomplete conversion and only partial recovery of the starting cations on back electrolysis at potentials negative of the +/2+ redox couples. Exemplary results for the methyl-substituted congener **2**^**+**^ are provided in Figure 9; those for the other two complexes are shown in Figure S70.

Oxidation transforms the ferrocenyl donor into a ferrocenium acceptor. The logical consequence is the bleaching of the y- and y_M_-bands, which is complete for **1**^**+**^, but incomplete for **2**^**+**^ and **3**^**+**^, likely as the result of only partial conversion within the optical path length of the spectroelectrochemical cell. In the case of **1**^**2+**^, the remaining anisyl donors are able to partially compensate the loss of the ferrocenyl donor, which results in a red shift of the x band. The same reason underlies the partial bleaching of the prominent band at 486 nm, which originates from a mixed ferrocene-to-diarylmethylium CT and a π−π* transition within the fluorenylium-type residue. For **2**^**2+**^ with the weaker tolyl donors, the position of the x-band remains nearly invariant, while for **3**^**2+**^ with electron-withdrawing trifluoromethyl substituents, a hypsochromic shift ensues.

**Figure 10:**
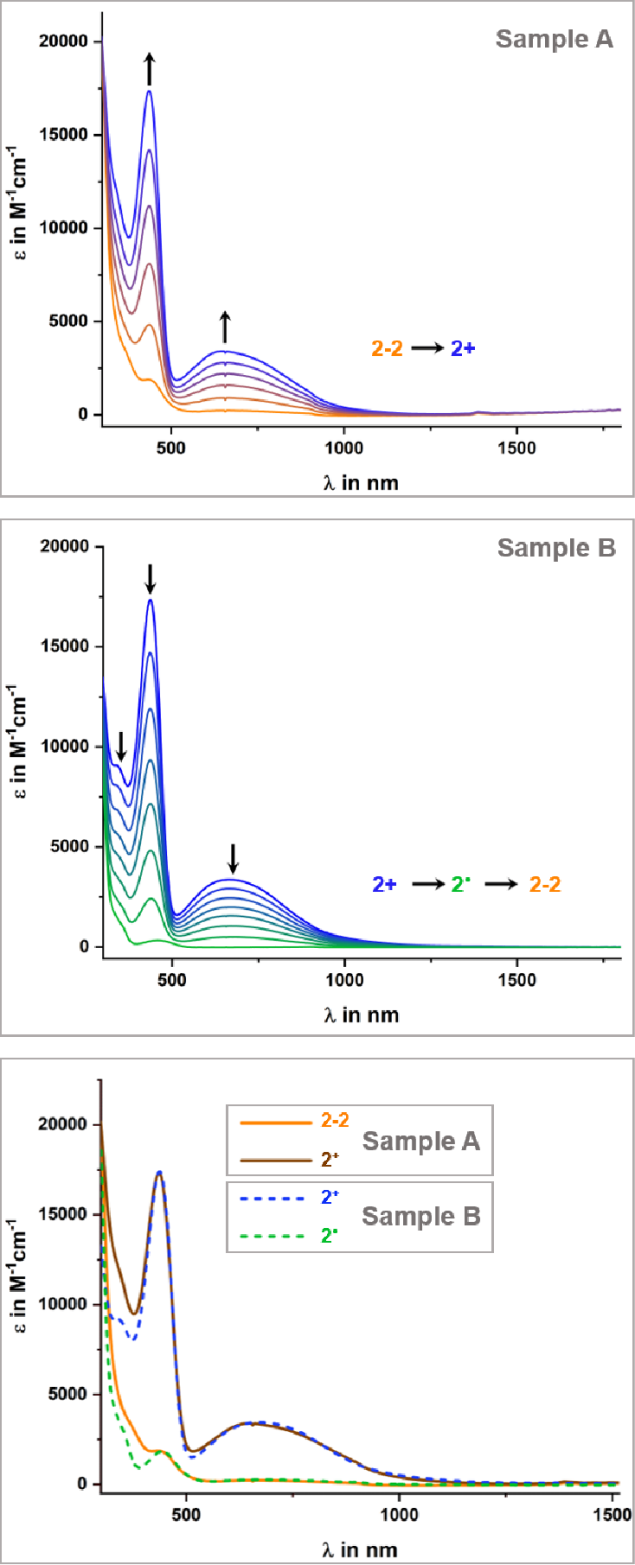
Sample A (top): changes in UV/vis/NIR spectra of the as-synthesized mixture of isomers **2**–**2** during oxidation. Sample B (middle): changes in UV/vis/NIR spectra during reduction of **2**^**+**^ (measurements in 1,2-C_2_H_4_Cl_2_ with the NBu_4_^+^ electrolyte at *T* = 293(±3) K. Bottom: overlay of the spectra of Samples A and B recorded before and after electrolysis.

Conversion of the fluorenylium acceptor to a fluorenyl radical during reduction results in the complete bleaching of all CT bands (cf. Figure 9, bottom, and Figure S70) so that the solution color changes to light yellow. According to the results detailed in the previous sections, the latter species are expected to exist almost exclusively as the corresponding dimers **1**–**1** to **3**–**3**. In order to further probe this contingency, we also subjected the mixture of isomers of dimer **2**–**2** to spectroelectrochemistry. The results of this study are shown in Figure 10 (sample A). In full agreement with the reaction schemes of Figures 6 and Scheme 7, oxidation to [**2**–**2**]**^+^** and then [**2**–**2**]^2+^ results in dissociation of the dimers into monomers **2**^**+**^. We emphasize that the spectral profile of the species formed upon oxidizing **2**–**2** is identical to that of an authentic sample of **2**^**+**^ (cf. Figure 10). Likewise, spectra recorded after exhaustive reduction of **2**^**+**^ (sample B in Figure 10) provide a nearly perfect match to those of as-synthesized **2**–**2**. These findings prove that the dimerization of the cationic complexes upon reduction and the dissociation of the oxidized dimers into monomeric cationic complexes establish a chemically reversible cycle of electron-transfer-induced C–C bond formation and scission.

## Radical Species and Dimerization

**Figure 11:**
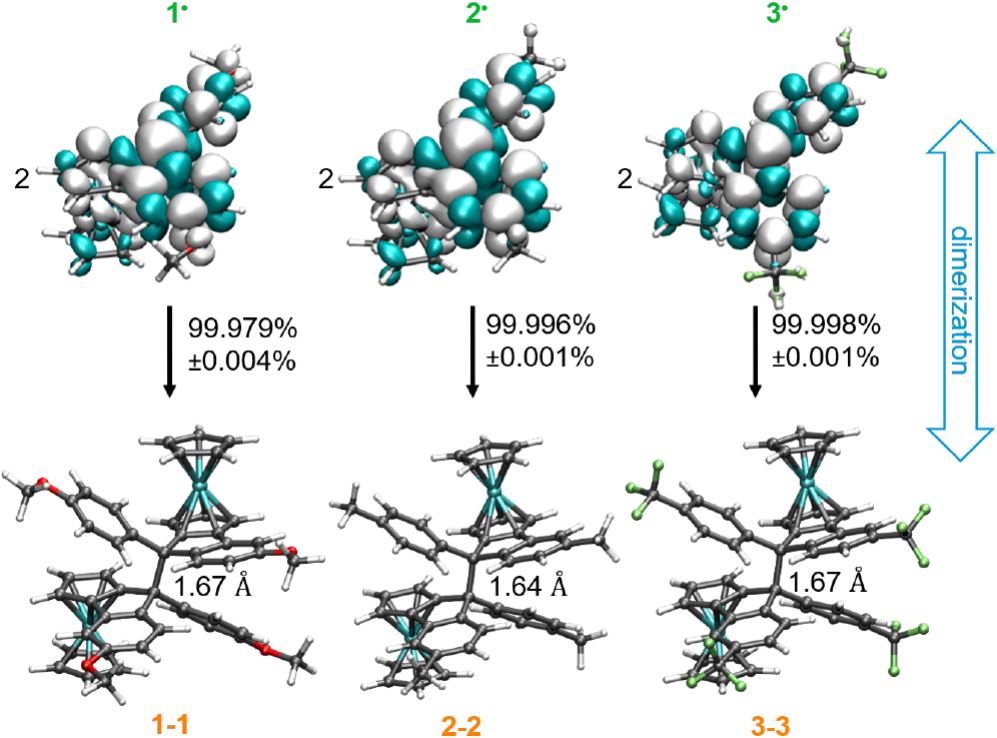
DFT-calculated spin densities of the monomeric radicals **1**^•^–**3**^•^ and geometry-optimized structures of the *Sp*-*exo*-*exo*-*Sp* isomer of their dimers.

As shown above, radicals **1**^•^–**3**^•^ have a large propensity to dimerize so that only a small fraction will exist as monomers. The inherently high sensitivity of EPR spectroscopy might nevertheless render them observable. We were indeed able to record EPR spectra of **1**^•^–**3**^•^, when high sample concentrations of 35 mmol L^–1^ were employed. Samples of **1**^•^–**3**^•^ were generated by chemical reduction of the parent cationic compounds with 1.1 eq. of decamethylferrocene (*E*_1/2_^0/+^ (Cp*_2_Fe) = −0.54 V) for **2**^**+**^ and **3**^**+**^, or cobaltocene (*E*_1/2_^0/+^ (Cp_2_Co) = −1.33 V) for **1**^**+**^ as reductants.^55^ Spectra recorded at different temperatures are provided in Figure S71. DFT-calculated spin densities of the radical monomers **1**^•^–**3**^•^ as well as the DFT-optimized structures of one specific isomer of the dimers (*Sp*-*exo*-*exo*-*Sp* according to Figure S64) are shown in Figure 11.

Even at the high concentrations employed in this study, the observed EPR signals were very weak in intensity. Signal-to-noise ratios remained poor, especially for **3**^•^, even after accumulating 60 scans (cf. Figure S71). We determined the effective spin concentrations by EPR spin counting (cf. Experimental Section) and compared them to the nominal concentrations, which were determined by assuming quantitative reduction of the cations. Comparison indicates an extent of dimerization of ≥99.98% at 20 °C (cf. values in Figure 11). The derived equilibrium constant *K*_dim_ of ca. 1 × 10^6^ L mol^–1^ is consistent with the value of *K*_dim_ of 4 × 10^5^ L mol^–1^ for the equilibrium 2 **1**^•^ ⇄ **1**–**1** derived from our voltammetric studies, in particular, when considering the differences in concentrations used in both kinds of experiments (ca. 1 mmol L^–1^ vs 35 mmol L^–1^).

Any monomer ⇄ dimer equilibrium will shift toward the dimer as *T* is lowered. Indeed, the EPR signal intensity decreases even further when the *T* is changed from 20 °C to −100 °C (cf. Figure S71). *T*-induced changes are reversed on rewarming the solutions, reconfirming the reversibility of the underlying process according to the concept of dynamic covalent chemistry.^26^ We must however mention that at *T* = 20 and 0 °C, two different EPR signals were observed for **1**^•^–**3**^•^ each (cf. Figure S71), but not at lower *T*. The major component, which represents ca. 60–80% of the entire sample, has a *g*-value of ca. 2.005, which is rather close to the free electron value *g*_e_ of 2.0023 (cf. Table S3), whereas the *g*-value of the minor component is larger, ca. 2.030. The reversible disappearance and reappearance of the latter signal on cooling/warming makes it unlikely that it results from partial decomposition or impurities. We are rather inclined to assign them to different diastereoisomers of the radical, arising from the configuration at the methyl C atom with the 9-phenyl substituent in either the *endo*- or *exo*-position (cf. Figure S2). Individual isomers may differ in spin density distributions and *g*-values and equilibrate so that higher proportions of a slightly less stable isomer form at higher *T*. The conjecture of at least two potential radical isomers agrees with our finding that dimer **2**–**2** forms a complex mixture of several species, in different proportions, as indicated by NMR spectroscopy (cf. Figure 3).

Calculated spin densities of the free radicals are delocalized over the molecules as shown in Figure 11 with 48.8% (**1**^•^), 49.9% (**2**^•^), and 50.2% (**3**^•^) of Mulliken spin densities on the methyl C atom. McGlinchey et al. have shown that the 9-ethynylferocenyl-substituted fluorenyl radical forms an HPE-like dimer with a C–C bond length of 1.535(10) Å.^42^ We expect the same kind of HPE structures in the present dimers. DFT-computed geometry-optimized structures of the *Sp*-*exo*-*exo*-*Sp* isomer of each dimer are shown at the bottom of Figure 11. Our calculations provided C–C bond lengths of 1.64 Å for **2**–**2** and 1.67 Å for **1**–**1** and **3**–**3**, which agrees well with the values of 1.608(3) to 1.645(6) Å for macrocyclic 9-aryl-substituted spirobifluorenes.^56,57^

## Conclusions

We present three new cationic triarylmethylium dyes **1**^**+**^–**3**^**+**^ with a common ferroceno[2,3]indenylmethylium skeleton with different *para*-substituents at the phenyl rings. Spectroscopic data as well as the results of DFT calculations indicate that the positive charge is delocalized over the entire π-conjugated skeleton, including the Cp deck of the incorporated ferrocenyl unit. Electronic absorption spectra reveal systematic shifts of all charge transfer bands to higher energies as the *para-*substituents become weaker donors/stronger acceptors, i.e., in the order OMe < Me < CF_3_. The CT character of all bands makes the electronic spectra of complexes **1**^**+**^–**3**^**+**^ responsive to redox transformations. Thus, one-electron oxidation selectively bleaches excitations originating from the incorporated ferrocene donor, whereas one-electron reduction causes all CT bands to vanish.

A key point of the present work was to provide detailed insights into the chemically reversible cycle that interconnects the cationic complexes, the one-electron reduced radicals and their corresponding dimers. For this purpose, the underlying reaction scheme was scrutinized by several analytical methods. Dimerization of **1**^•^, i.e., the one-electron reduced form of **1**^**+**^, occurs at a rate faster than the voltammetric time scale, while being much slower for the other two congeners. Digital simulation of cyclic voltammograms yielded an equilibrium constant *K*_dim_ of 4 × 10^5^ L mol^–1^ and a rate constant in the order of 1.7 × 10^5^ s^–1^ for dimerization. The opposite process, i.e., the splitting of the neutral dimers on stepwise oxidation to their corresponding dications, was studied at the example of dimer **2**–**2**. The latter was found to exist as a complex mixture of isomers, resulting from the presence of *Rp* and *Sp* enantiomers of the planar chiral, unsymmetrically 1,2-disubstituted ferrocene as well as an *exo-* or *endo*-positioning of the 9-phenyl substituent at the methyl carbon atom. The chemical reversibility of the process 2 **2**^**+**^ ⇄ 2 **2**^•^ ⇄ **2**–**2** was further probed by spectroelectrochemistry. Changes in the electronic spectra observed during the reduction of **2**^**+**^ are the exact opposite of those, when dimer **2**–**2** is oxidized. The large displacement of the monomer ⇄ dimer equilibria to the side of the dimers requires highly concentrated solutions of the one-electron reduced species in order to render the free radicals observable by EPR spectroscopy. Comparison between the free radical concentrations as determined via spin counting and the nominal sample concentrations indicates that ≥99.98% of their neutral forms are trapped in the dimers, providing an estimate of the equilibrium constants *K*_dim_ of the order of 1 × 10^6^ L mol^–1^, which is fully consistent with the results of our voltammetric studies on complex **1**^**+**^.

## Experimental Section

All syntheses and other manipulations (e.g., reductions and oxidations) were carried out under an atmosphere of purified nitrogen with dry, distilled, and nitrogen-saturated solvents. Standard Schlenk and glovebox techniques were applied. All reagents were purchased from commercial suppliers and used without further purification. The supporting electrolyte NBu_4_^+^ [B{C_6_H_3_(CF_3_)_2_-3,5}_4_]^−^ was synthesized in a two-step procedure starting from 3,5-bis(trifluoromethyl)bromobenzene to first give Na^+^ [B{C_6_H_3_(CF_3_)_2_-3,5}_4_]^−^,^58^ which was subsequently subjected to cation exchange.^59^ Brookhart’s acid was synthesized following a published protocol.^44^

^1^H NMR (400 MHz), ^19^F NMR (376 MHz), and ^13^C NMR (101 MHz) spectra were recorded on a Bruker Avance III 400 spectrometer in CD_2_Cl_2_. The spectra were referenced to the residual protonated solvent (^1^H) or the solvent signal (^13^C). ^19^F NMR spectra were referenced to the external reference of the spectrometer (δ(^19^F) = 0 ppm for CFCl_3_). The FT-IR spectrum of dimer **2**–**2** was recorded on a Bruker Tensor III instrument in a range between 1000 and 11 500 cm^–1^. MS spectra were recorded on a Pierce LTQ Velos ESI-calibrated LTQ Orbitrap Velos Spectrometer with the ESI method at a spray voltage of 4.0 kV. Detection was done in the positive-ion mode using dichloromethane as the solvent. A positive calibration solution was used prior to every measurement. Elemental analyses (EA) were performed on a C,H,N-analyzer (model Elementar vira MICRO Cube) by *Heraeus*.

For X-ray crystallography, a STOE IPDS-II diffractometer equipped with a graphite-monochromated radiation source (λ[Mo–K_α_]) and an image plate detection system was used. Data collection was performed at 100 K. The X-Area software package was used for data processing (e.g., selection, integration, averaging procedure of the measured reflection intensities, determination of unit cell dimensions, data reduction, LP correction, space group determination). A semiempirical absorption correction was performed, and the structure was solved by the heavy-atom method. Structure solution was completed with difference Fourier syntheses and full-matrix least-squares refinements using SHELX-2018/3 in combination with OLEX2,^60−62^ minimizing ω(*F*_o_^2^ – *F*_c_^2^)^2^. The weighted *R* factor (w*R*^2^) and the goodness-of-fit GOOF are based on *F*^2^. All nonhydrogen atoms were refined with anisotropic displacement parameters, and hydrogen atoms were introduced in a riding model.

All electrochemical experiments were conducted under argon atmosphere. Samples of the cationic complexes **1**^**+**^–**3**^**+**^ were freshly generated (*in situ*) using 4 Å pore-size molecular sieves. The data was acquired with a computer-controlled *BASi* Epsilon potentiostat. A custom-built cylindrical, vacuum-tight one-compartment cell equipped with a platinum working electrode was used. A spiral-shaped Pt wire was introduced as a counter and a Ag wire as a (*pseudo*) reference electrode. These electrodes are sealed into glass capillaries and fixed by Quickfit screws. The working electrode was polished with first 1 μm and then 0.25 μm diamond pastes prior to measurements. The working electrode is introduced into the top port of the one-compartment cell that was attached to a conventional Schlenk line by a side arm through a Teflon screw cap with a suitable fitting. NBu_4_^+^ [B{C_6_H_3_(CF_3_)_2_-3,5}_4_]^−^ was used as the supporting electrolyte in dichloromethane as the solvent. Oxygen was removed by bubbling argon via a cannula for 2 min prior to measurement. After data acquisition was complete, roughly equimolar amounts of decamethylferrocene (Cp*_2_Fe), ferrocene (Cp_2_Fe), or cobaltocenium hexafluorophosphate ([Cp_2_Co]^+^ [PF_6_]^−^) were added as internal standards for referencing. Final referencing was done, after repetition of every measurement with the added standard, against the ferrocene/ferrocenium (Cp_2_Fe^0/+^) redox couple with *E*_1/2_(Cp*_2_Fe^0/+^) = −540 mV and *E*_1/2_(Cp_2_Co^+/0^) = −1330 mV vs *E*_1/2_(Cp_2_Fe^0/+^).^55^

Simulations of cyclic voltammograms in this work were performed using the program DigiSim.^63^ By loading cyclic voltammograms acquired over a range of scan rates of 50–2000 mV/s, the program was free to change the chosen input parameters in an interactive manner to optimally replicate the experimental CV shapes. The initial parameters were set as follows: Initial concentration = 0.0023 M (based on the concentration of the carbinol precursor, and assuming complete conversion of the carbinol to the cationic complex), electrode surface area = 0.0256 cm^2^, *T* = 298 K, Cdl = 3 × 10^–7^ F, planar electrode geometry, electron transfer coefficient α = 0.5; electron transfer rate *k*_s_ = 0.01 cm s^–1^. The diffusion coefficient of **1**^**+**^ was determined by CV simulation of the current of the cathodic forward peak I in Figure 5.

UV/vis/NIR spectra were recorded on a *TIDAS* fiber optic diode array spectrometer, which consists of a combination of MCS UV/NIR and PGS NIR instruments from *J&M*. For the determination of extinction coefficients, *Hellma* quartz cuvettes with 0.1, 0.5, and 1.0 cm optical path lengths were used (filled under nitrogen atmosphere inside a glovebox). A custom-built optically transparent thin-layer electrochemical (OTTLE) cell (Pt-mesh working and counter electrodes, a thin silver wire as a *pseudo* reference electrode, CaF_2_ windows) manufactured according to Hartl’s design,^64^ was used for spectroelectrochemical measurements, using 1,2-C_2_H_4_Cl_2_ as the solvent.

Electron paramagnetic resonance (EPR) studies were conducted on an *X*-band spectrometer MiniScope MS5000 by Magnettech GmbH in combination with the program ESR Studio 1.63.0 in the temperature range from 20 °C to −150 °C.^65^ The liquid nitrogen-cooled thermostat was used in combination with the temperature controller HO3. EPR samples in dichloromethane were prepared and sealed inside a glovebox. All experiments were performed with identical measurement parameters: *B* = 330–345 mT, sweep time = 60 s, modulation = 0.3 mT, power = 6.3096 mW. The same glass tubes (3 mm outer and 2 mm inner diameter) were used with the same filling height of 60 mm to ensure the comparability of quantitative measurements. DPPH^•^ was used as a calibrating agent. It was checked for purity by titration with hydroquinone using a UV/vis/NIR probe.^66^ The quantitative evaluation of dimerization via EPR spectroscopy using the diphenylpicrylhydrazyl (DPPH^•^) regression line follows a published method for spin counting.^33^ Simulation of the measured EPR spectra was performed using the *Matlab* Easyspin program “*garlic*”.^67^

Density functional theory (DFT) calculations were conducted using the Gaussian 16 program package.^68^ Geometry optimization followed by vibrational analysis were performed in 1,2-dichloroethane as the solvent, applying the polarizable continuum model (PCM) to eliminate imaginary frequencies.^69^ Triplet states were tested for wave function instability and reoptimized, if necessary. Electronic spectra were calculated at the optimized ground-state structures by the TD-DFT method. A Wachter basis set^70^ was used for the Fe atom, and a 6-316(d)^71^ polarized double-ξ basis set with PBE0^72,73^ as correlation functional was used for the remaining atoms (e.g., C, H, O, S, F). The GaussSum,^74^ Avogadro, GNU Parallel, and vmd program packages were used in combination with POV-Ray for data processing^75^ and graphical representations.^75,76^

The synthetic procedures toward **1-CO**^**R**^**-PhI**–**3-CO**^**R**^**-PhI** (*R* = OMe, Me, CF_3_) and ketones **1′**–**3′** are provided in the Supporting Information.

### **General Synthesis of Carbinols 1-OH**–**3-OH**

The conversion of the ketones to the carbinols is based on a literature method for related compounds.^37^ Magnesium turnings (1.71 equiv) were suspended in 10 mL of dry, degassed diethyl ether (**1-OH** and **2-OH**) or THF (**3-OH**). Two drops of 1,2-dibromoethane were added to the suspension. A solution of the respective 1-bromo-4-*R*-benzene derivative (1.46 equiv) in dry degassed diethyl ether/THF (30 mL) was added while heating the mixture for the Grignard reaction to start. Then, the mixture was heated to reflux (oil bath) for 30 min until the magnesium turnings were completely dissolved. A solution of the respective ferroceno[2,3]-inden-1-one derivative **1′**–**3′** (1.0 equiv) in dry, degassed diethyl ether/THF (30 mL) was added dropwise. The mixture was heated to reflux for 40 min, then quenched with water (50 mL). The aqueous and organic phases were separated, and the aqueous phase was extracted with diethyl ether (3 × 100 mL). The organic phases were combined and dried over Na_2_SO_4_, and the solvent was removed under reduced pressure. The orange to brownish residue was purified by gradient column chromatography on silica gel (PE/EE 1:0–20:1) to yield two isomers (*Sp* and *Rp*) of *endo***1-OH**–**3-OH** as orange solids.

#### 9-(4-Methoxy)phenylferrocene(1,2,3,3*a*,8*a*-)1,8-dihydrocyclo-pent[*a*]inden-8-ol (**1-OH**)

A yield of 30 mg (70 μmol, 18%) of orange, microcrystalline **1-OH** was obtained for a batch size of 0.40 mmol (1 equiv of **1′**).^37^**^1^H NMR** (400 MHz, CD_2_Cl_2_) δ [ppm] = 7.20 (d, ^3^*J*_HH_ = 9.0 Hz, 2H, H-8), 7.06 (d, ^3^*J*_HH_ = 8.3 Hz, 1H, H-13), 6.90 (d, ^4^*J*_HH_ = 2.4 Hz, 1H, H-17), 6.73 (d, ^3^*J*_HH_ = 9.0 Hz, 2H, H-9), 6.65 (dd, ^3^*J*_HH_ = 8.3 Hz, ^4^*J*_HH_ = 2.4 Hz, 1H, H-14), 4.66 (d, ^3^*J*_HH_ = 3.1 Hz, 1H, H-2/H-4), 4.34 (vt, ^3^*J*_HH_ = 3.1 Hz, ^3^*J*_HH_ = 2.4 Hz, 1H, H-3), 4.25 (d, ^3^*J*_HH_ = 2.4 Hz, 1H, H-2/H-4), 4.14 (s, 5H, H-1), 3.82 (s, 3H, H-16), 3.73 (s, 3H, H-11), 2.96 (s, 1H, H-20). ^**13**^**C{**^**1**^**H} NMR** (101 MHz, CD_2_Cl_2_) δ [ppm] = 160.6 (C-15), 159.0 (C-10), 147.5 (C-12), 141.1 (C-18), 137.1 (C-7), 126.9 (C-8), 125.4 (C-13), 113.7 (C-9), 111.4 (C-14), 106.8 (C-17), 104.8 (C-5), 89.8 (C-19), 79.1 (C-6), 70.9 (C-3), 70.1 (C-1), 62.4 (C-2/C-4), 60.7 (C-2/C-4), 55.8 (C-16), 55.6 (C-11). **HRMS (ESI/ion trap)***m*/*z*: M^+^ calcd for C_25_H_22_FeO_3_ 426.0918; found 426.0915.

#### 9-(4-Methyl)phenylferrocene(1,2,3,3*a*,8*a*-)1,8-dihydrocyclo-pent[*a*]inden-8-ol (**2-OH**)

A yield of 275 mg (697 μmol, 70%) of orange, single crystals of **2-OH** suitable for SCXRD analysis were obtained by slow vapor diffusion of *n*-pentane into a concentrated solution of **2-OH** in dichloromethane for a batch size of 0.99 mmol (1 equiv of **2′**).^37^**^1^H NMR** (400 MHz, CD_2_Cl_2_) δ [ppm] = 7.17 (s, 1H, H-17), 7.15 (d, ^3^*J*_HH_ = 8.3 Hz, 2H, H-8), 7.03 (d, ^3^*J*_HH_ = 6.8 Hz, 1H, H-13), 7.01 (d, ^3^*J*_HH_ = 8.3 Hz, 2H, H-9), 6.93 (d, ^3^*J*_HH_ = 6.8 Hz, 1H, H-14), 4.65 (d, ^3^*J*_HH_ = 2.3 Hz, 1H, H-2/H-4), 4.33 (br s, 1H, H-3), 4.22 (d, ^3^*J*_HH_ = 2.4 Hz, 1H, H-2/H-4), 4.13 (s, 5H, H-1), 2.97 (s, 1H, H-20), 2.35 (s, 3H, H-16), 2.26 (s, 3H, H-11). ^**13**^**C{**^**1**^**H} NMR** (101 MHz, CD_2_Cl_2_) δ [ppm] = 152.6 (C-18), 141.9 (C-7), 139.5 (C-12), 138.5 (C-15), 137.0 (C-10), 129.1 (C-9), 127.2 (C-14), 125.6 (C-8), 124.5 (C-13), 121.5 (C-17), 104.2 (C-5), 90.5 (C-19), 79.5 (C-6), 70.8 (C-3), 70.1 (C-1), 62.3 (C-2/H-4), 60.7 (C-2/H-4), 21.6 (C-16), 21.1 (C-11). **HRMS (ESI/ion trap)***m*/*z*: M^+^ calcd for C_25_H_22_FeO 394.1015; found 394.0995.

#### 9-(4-Trifluoromethyl)phenylferrocene(1,2,3,3*a*,8*a*-)1,8-dihydrocyclo-pent[*a*]inden-8-ol (**3-OH**)

A yield of 84 mg (168 μmol, 65%) of orange, crystalline **3-OH** was obtained for a batch size of 0.26 mmol (1 equiv of **3′**).^37^**^1^H NMR** (400 MHz, CDCl_3_) δ [ppm] = 7.62 (s, 1H, H-17), 7.49 (d, ^3^*J*_HH_ = 8.4 Hz, 2H, H-9), 7.42 (d, ^3^*J*_HH_ = 8.4 Hz, 2H, H-8), 7.40 (d, ^3^*J*_HH_ = 7.9 Hz, 1H, H-14), 7.25 (d, ^3^*J*_HH_ = 7.9 Hz, 1H, H-13), 4.80 (d, ^3^*J*_HH_ = 2.1 Hz, 1H, H-2), 4.46 (dd, ^3^*J*_HH_ = 2.3 Hz, ^3^*J*_HH_ = 2.1 Hz, 1H, H-3), 4.31 (d, ^3^*J*_HH_ = 2.3 Hz, 1H, H-4), 4.18 (s, 5H, H-1), 3.16 (s, 1H, H-20). ^**13**^**C{**^**1**^**H} NMR** (101 MHz, CDCl_3_) δ [ppm] = 158.2 (C-12), 147.9 (C-7), 141.0 (C-18), 131.2 (d, ^2^*J*_CF_ = 32 Hz, C-15), 129.6 (d, ^2^*J*_CF_ = 32 Hz, C-10), 126.2 (C-8), 125.5 (q, ^3^*J*_CF_ = 4 Hz, C-9), 125.2 (C-13), 124.7 (d, ^1^*J*_CF_ = 273 Hz, C-11), 124.8 (d, ^1^*J*_CF_ = 274 Hz, C-16), 123.7 (q, ^3^*J*_CF_ = 4 Hz, C-14), 117.5 (q, ^3^*J*_CF_ = 4 Hz, C-17), 103.3 (C-5), 88.8 (C-19), 79.3 (C-6), 72.0 (C-3), 70.4 (C-1), 63.0 (C-2/C-4), 61.7 (C-2/C-4). ^**19**^**F{**^**1**^**H} NMR** (376 MHz, CDCl_3_) δ [ppm] = −62.7 (CF_3_-11), −62.8 (CF_3_-16). **HRMS (ESI/ion trap)***m*/*z*: M^+^ calcd for C_25_H_16_F_6_FeO 502.0455; found 502.0455.

### General Synthesis of Cationic Complexes 1^+^–3^+^

Brookhart’s acid ([H(OEt_2_)_2_]^+^ [B{C_6_H_3_(CF_3_)_2_-3,5}_4_]^−^ = , 1.0 equiv) was added to a dry, degassed dichloromethane solution of the respective carbinol precursor **1-OH**–**3-OH** in the presence of molecular sieves (4 Å). This caused an immediate, intense colorization of the solution to different shades of green. Quantitative removal of the solvent afforded the  salts of the cationic dyes as intensely colored, amorphous solids. Due to gradual decomposition in solution and in the solid state when stored at r.t., the complexes were freshly synthesized before every measurement in order to obtain reproducible results. Yet, the cationic complexes can be stored with only minor degradation inside a nitrogen-filled glovebox in a fridge. *In situ* synthesis and consecutive direct measurements are also possible despite residual diethyl ether remaining from the applied Brookhart’s acid.

#### 9-(4-Methoxy)phenylferrocene(1,2,3,3*a*,8*a*-)1,8-dihydrocyclo-pent[*a*]indenyl Methylium (**1**^**+**^)

The product was obtained following the “general synthesis of cationic complexes” as a dark green solid. ^**1**^**H NMR** (400 MHz, CD_2_Cl_2_) δ [ppm] = 7.97 (d, ^3^*J*_HH_ = 9.1 Hz, 2H, H-9), 7.53 (d, ^3^*J*_HH_ = 8.9 Hz, 1H, H-13), 6.95 (d, ^3^*J*_HH_ = 9.1 Hz, 2H, H-8), 6.75 (vt, ^3^*J*_HH_ = 2.6 Hz, 1H, H-3), 6.41 (d, ^4^*J*_HH_ = 2.4 Hz, 1H, H-17), 6.18 (dd, ^3^*J*_HH_ = 8.9 Hz, ^4^*J*_HH_ = 2.4 Hz, 1H, H-14), 6.17–6.14 (m, 1H, H-2/H-4), 4.59 (s, 5H, H-1), 3.87 (s, 3H, H-11), 3.74 (s, 3H, H-16). ^**13**^**C{**^**1**^**H} NMR** (101 MHz, CD_2_Cl_2_) δ [ppm] = 165.3 (C-10), 164.2 (C-15), 163.5 (C-7), 145.3 (C-18), 143.7 (C-12), 137.4 (C-6), 130.7 (C-9), 128.1 (C-13), 118.4 (C-8), 118.2 (C-14), 111.1 (C-17), 91.5 (C-3), 89.4 (C-5), 86.0 (C-1), 81.4 (C-19), 79.2 (C-2/C-4), 76.9 (C-2/C-4), 57.1 (C-16), 56.9 (C-11). **HRMS (ESI/ion trap)***m*/*z*: M^+^ calcd for C_25_H_21_FeO_2_ 409.0885; found 409.0862. Anal. calcd for C_57_H_33_BF_24_FeO_2_: C, 53.80; H, 2.61. Found: C, 53.86; H, 3.55.

#### 9-(4-Methyl)phenylferrocene(1,2,3,3*a*,8*a*-)1,8-dihydrocyclo-pent[*a*]indenyl Methylium (**2**^**+**^)

The product was obtained following the “general synthesis of cationic complexes” as a dark green solid. ^**1**^**H NMR** (400 MHz, CD_2_Cl_2_) δ [ppm] = 7.91 (d, ^3^*J*_HH_ = 8.4 Hz, 2H, H-8), 7.46 (d, ^3^*J*_HH_ = 8.0 Hz, 1H, H-13), 7.19 (d, ^3^*J*_HH_ = 8.4 Hz, 2H, H-9), 6.95 (vt, ^3^*J*_HH_ = 2.7 Hz, 1H, H-3), 6.47 (br s, 1H, H-17), 6.42 (d, ^3^*J*_HH_ = 8.0 Hz, 1H, H-14), 6.39 (d, ^3^*J*_HH_ = 2.7 Hz, 1H, H-2/H-4), 6.28 (d, ^3^*J*_HH_ = 2.8 Hz, 1H, H-2/H-4), 4.67 (s, 5H, H-1), 1.93 (s, 3H, H-11), 1.52 (s, 3H, H-16). ^**13**^**C{**^**1**^**H} NMR** (101 MHz, CD_2_Cl_2_) δ [ppm] = 150.8 (C-6) 146.6 (C-10), 143.7 (C-15), 139.4 (C-18), 134.5 (C-14), 133.9 (C-9), 126.7 (C-8), 126.4 (C-17), 125.7 (C-7), 123.7 (C-14), 121.0 (C-12), 111.9 (C-5), 95.2 (C-3), 91.8 (C-19), 88.7 (C-1), 84.4 (C-2/C-4), 77.1 (C-2/C-4), 23.4 (C-11), 23.3 (C-16). **HRMS(ESI/ion trap)***m*/*z*: M^+^ calcd for C_25_H_21_Fe 377.0987; found 377.0985. Anal. calcd for C_57_H_33_BF_24_Fe: C, 55.19; H, 2.68. Found: C, 55.19; H, 4.56.

#### 9-(4-Trifluoromethyl)phenylferrocene(1,2,3,3*a*,8*a*-)1,8-dihydrocyclo-pent[*a*]indenyl Methylium (**3**^**+**^)

The product was obtained following the “general synthesis of cationic complexes” as a dark green solid. ^**1**^**H NMR** (400 MHz, CD_2_Cl_2_) δ [ppm] = 8.13 (d, ^3^*J*_HH_ = 8.1 Hz, 2H, H-9), 7.66 (d, ^3^*J*_HH_ = 8.2 Hz, 2H, H-8), 7.63 (d, ^3^*J*_HH_ = 8.1 Hz, 1H, H-13), 7.05 (vt, ^3^*J*_HH_ = 2.7 Hz, 1H, H-3), 6.98 (d, ^3^*J*_HH_ = 8.1 Hz, 1H, H-14), 6.84 (s, 1H, H-17), 6.63 (d, ^3^*J*_HH_ = 2.7 Hz, 1H, H-2), 6.48 (d, ^3^*J*_HH_ = 2.7 Hz, 1H, H-4), 4.88 (s, 5H, H-1). ^**13**^**C{**^**1**^**H} NMR** (101 MHz, CD_2_Cl_2_) δ [ppm] = 156.7 (C-6), 139.8 (C-10), 136.4 (C-15), 135.0 (C-7), 133.9 (C-12), 130.3 (C-14), 130.1 (C-8), 126.1 (C-9), 125.5 (C-11), 124.6 (C-5), 123.1 (C-16), 122.8 (C-18), 121.8 (C-17), 121.0 (C-13), 99.3 (C-3), 92.4 (C-19), 91.5 (C-1), 88.1 (C-4), 77.5 (C-2). ^**19**^**F{**^**1**^**H} NMR** (376 MHz, CD_2_Cl_2_) δ [ppm] = −64.5 (CF_3_), –65.7 (CF_3_). **HRMS (ESI/ion trap)***m*/*z*: M^+^ calcd for C_25_H_15_F_6_Fe 485.0422; found 485.0431. Anal. calcd for C_57_H_27_BF_30_Fe: C, 50.77; H, 2.02. Found: C, 50.45; H, 3.68.

#### Bis-9-(4-methyl)phenylferrocene(1,2,3,3a,8a-)1,8-dihydrocyclo-pent[a]indenyl Dimer (**2**–**2**)

**2-OH** (30 mg, 64 μmol, 1.0 equiv) was dissolved in dry, degassed dichloromethane (5 mL) under inert gas conditions. Addition of Brookhart’s acid (256 mg, 64 μmol, 1.0 equiv) caused an immediate change of solution color from orange to dark green. Decamethylferrocene (20 mg, 64 μmol, 1.0 equiv) reductant was added to the solution, and the mixture was stirred inside a glovebox for 64 h at r.t. under inert conditions. The solvent was removed *in vacuo* and the dark turquoise residue (the color results from the decamethylferrocenium byproduct) was purified by column chromatography on silica gel under ambient atmosphere (*n*-pentane/ethyl acetate 1:0–1:4) to yield **2**–**2** in 80% yield as a light orange, microcrystalline solid (16 mg, 24 μmol, 0.80 equiv). ^**1**^**H NMR** (400 MHz, CD_2_Cl_2_) δ [ppm] = 7.75–6.93 (m, 14H, H_Ph_, H_FLO-6-Ring_), 4.66–4.21 (m, 6H, H_Fc-FLO_), 3.59–3.44 (m, 10H, H_Cp_), 2.49–2.35 (m, 12H, H_Me_). **HRMS (ESI/ion trap)***m*/*z*: M^+^ Calcd for C_25_H_21_Fe 377.0987; found 377.0995.

## Data Availability

The data underlying this study are available in the published article and the accompanying Supporting Information.

## Supporting Information Available

Synthesis of precursors **1-CO-PhI**–**3-CO-PhI** and **1′**–**3′**, additional characterization data (ESI-MS and NMR spectra), scheme of potential enantiomers (*endo*, *exo*, *Rp*, *Sp*), crystallographic data compilation from literature and crystal data for **2-OH**, optical impression of dye solutions, schematic structures of all possible isomers of dimers **2**–**2**, comparison of DFT-calculated and recorded IR spectra of **2**–**2**, Hammett plots for the different redox processes, TD-DFT computed vis/NIR spectra of the complexes and charge density difference plots for relevant electronic transitions, UV/vis/NIR spectroscopic changes during the oxidation and reduction of **1**^**+**^ and **3**^**+**^, EPR spectroscopic data of reduced **1**^•^–**3**^•^, and DFT-computed energies and xyz-coordinates of the geometry-optimized structures (PDF)
