# Supplementary material for: Dimerization of 9-Phenyl-ferroceno[2,3]indenylmethyl Radicals: Electrochemical and Spectroelectrochemical Studies
Source: ACS Org Inorg Au. 2024 Apr 19;4(4):395–409. doi: 10.1021/acsorginorgau.3c00070 (PMC11311460; doi:10.1021/acsorginorgau.3c00070)
Supplement: Supplementary file 1 — gg3c00070_si_001.pdf [file gg3c00070_si_001.pdf]

Supporting Information for:

On the Dimerization of 9-Phenyl-ferroceno[2,3]indenylmethyl Radicals: Electrochemical and Spectroelectrochemical Studies

Larissa A. Casper, Katharina L. Deuter, Anja Rehse, Rainer F. Winter*

*Department of Chemistry, Universität Konstanz, 78457 Konstanz, Germany
Email: rainer.winter@uni-konstanz.de

Experimental Section

*(2-Iodo-4-methoxybenzoyl)ferrocene (*1-CO-^{MeO}PhI*)*

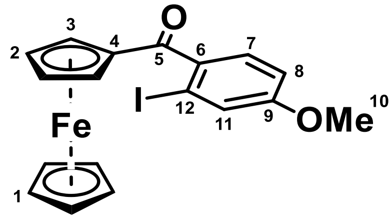

Under inert gas atmosphere, 2-iodo-4-methoxybenzoic acid[1] (0.28 g, 1.0 mmol, 1.0 eq.) and DMF (0.1 mL) were dissolved in dry, degassed dichloromethane (3 mL) and cooled to 0 °C. Oxalyl chloride (0.13 mL, 0.19 g, 1.5 mmol, 1.5 eq.) was added and the resulting green solution was stirred at r.t. for one hour. The solvent was removed *in vacuo* to obtain the acid chloride. The residue was redissolved in dry, degassed dichloromethane (2 mL) and added dropwise to a solution of ferrocene (0.29 g, 1.5 mmol, 1.5 eq.) and $AlCl_3$ (0.32 g, 2.4 mmol, 2.0 eq.) in dry, degassed dichloromethane (10 mL). The reaction mixture was stirred for 16 hours. During this time, the solution turned dark red. The reaction mixture was quenched by addition of ice, and the organic and aqueous phases were separated. The aqueous phase was extracted with dichloromethane (2 × 50 mL), the organic phases were combined, washed with water, and dried over $MgSO_4$. The solvent was removed under reduced pressure and the remaining dark red oil was purified by gradient column chromatography on silica gel (*n*-pentane/ethyl acetate 10:1 – 6:1) to yield **1-CO-^{MeO}PhI** as a dark red solid in 21% yield (98 mg, 0.21 mmol, 0.21 eq.).[2] **^{1}H-NMR** (400 MHz, $CDCl_3$) δ [ppm] = 7.47 (d, $^4J_{HH}$ = 2.5 Hz, 1H, H-11), 7.45 (d, $^3J_{HH}$ = 8.5 Hz, 1H, H-7), 6.92 (dd, $^3J_{HH}$ = 8.5 Hz, $^4J_{HH}$ = 2.5 Hz, 1H, H-8), 4.75 (vt, $^3J_{HH}$ = 2.0 Hz, 2H, H-2/3), 4.58 (vt, $^3J_{HH}$ = 2.0 Hz, 2H, H-2/3), 4.30 (s, 5H, H-1), 3.85 (s, 3H, H-10). **^{13}C{^{1}H}-NMR** (101 MHz, $CDCl_3$) δ [ppm] = 200.1 (C-5), 160.8 (C-9), 136.9 (C-6), 129.9 (C-7), 125.9 (C-11), 113.2 (C-8), 93.7 (C-12), 78.4 (C-4), 72.8 (C-2/3), 71.7 (C-2/3), 70.2 (C-1), 55.8 (C-10). **HRMS (ESI/ion trap)** m/z: $[M+Na]^+$ Calcd for $C_{18}H_{15}FeIO_2Na$ 468.9358; Found 468.9366.

*(2-Iodo-4-methylbenzoyl)ferrocene (*2-CO-^{Me}PhI*)*

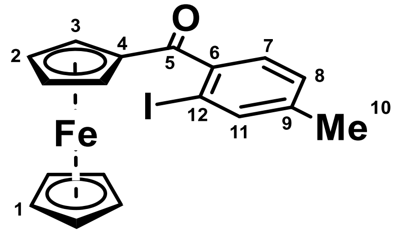

Under inert gas atmosphere, 2-iodo-4-methylbenzoic acid[1] (1.72 g, 6.6 mmol, 1.0 eq.) and DMF (0.1 mL) were dissolved in dry, degassed dichloromethane (40 mL) and cooled in an ice bath to 0 °C. Oxalyl chloride (1.2 mL, 1.78 g, 14.0 mmol, 2.2 eq.) was added and the resulting yellow solution was stirred at r.t. for one hour. The solvent was removed *in vacuo* to obtain the acid chloride. The residue was redissolved in dry, degassed dichloromethane (40 mL) and added dropwise to a solution of ferrocene (1.83 g, 9.9 mmol, 1.5 eq.) and $AlCl_3$ (2.28 g, 17.0 mmol, 2.6 eq.) in dry, degassed dichloromethane (120 mL). The reaction mixture was stirred for 16 hours, during which time it turned dark blue. The reaction mixture was quenched by the addition of ice, and the organic and aqueous phases were separated. The aqueous phase was extracted with dichloromethane (3 × 50 mL), the organic phases were combined, washed with water, and dried over Na_2SO_4. The solvent was removed under reduced pressure and the remaining dark red oil was purified by column chromatography on silica gel (*n*-pentane/ethyl acetate 4/1) to yield **2-CO-^{Me}PhI** as a dark red solid in 61% yield (1.71 g, 4 mmol, 0.61 eq.).[2] **^{1}H-NMR** (400 MHz, $CDCl_3$) δ [ppm] = 7.77 (s, 1H, H-11), 7.38 (d, $^3J_{HH}$ = 7.7 Hz, 1H, H-7), 7.21 (d, $^3J_{HH}$ = 7.7 Hz, 1H, H-8), 4.73 (vt, $^3J_{HH}$ = 2.0 Hz, 2H, H-2/3), 4.58 (vt, $^3J_{HH}$ = 2.0 Hz, 2H, H-2/3), 4.30 (s, 5H, H-1), 2.37 (s, 3H, H-10). **^{13}C{^{1}H}-NMR** (101 MHz, $CDCl_3$) δ [ppm] = 201.0 (C-5), 141.9 (C-6), 141.7 (C-9), 140.8 (C-11), 128.5 (C-7), 128.3 (C-8), 92.9 (C-12), 78.2 (C-4), 72.9 (C-2/3), 71.6 (C-2/3), 70.2 (C-1), 20.9 (C-10). **HRMS (ESI/ion trap)** m/z: $[M+Na]^+$ Calcd for $C_{18}H_{15}FeIONa$ 452.9409; Found 452.9411.

*(2-Iodo-4-trifluoromethylbenzoyl)ferrocene (*3-CO-^{CF3}PhI*)*

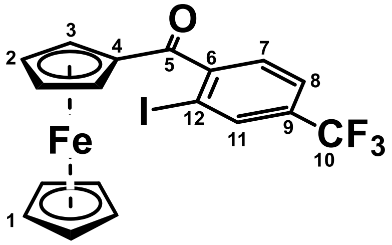

Thionyl chloride (3.25 mL, 5.30 g, 44.5 mmol, 13.7 eq.) and one drop of DMF were added to 2-Iodo-4-trifluoromethylbenzoic acid (1.03 g, 3.25 mmol, 1.0 eq.) and the reaction mixture was heated to reflux for 4 h. After cooling to r.t., excess thionyl chloride was removed *in vacuo* to give the acid chloride as a pale yellow oil in almost quantitative yield. The acid chloride was then dissolved in dry DCM (5 mL) and added dropwise to a mixture of ferrocene (0.91 g, 4.88 mmol, 1.5 eq.) and $AlCl_3$ (0.87 g, 6.5 mmol, 2.0 eq.) in 30 mL dry DCM. The reaction mixture was stirred at r.t. for 16 h, during which time it turned dark blue. The reaction mixture was poured onto ice water (50 mL), acidified with 1 mL conc. HCl and the phases were separated. The aqueous phase was extracted with DCM (3 × 20 mL). The combined organic phases were washed with water (2 × 30 mL) and dried over Na_2SO_4. The solvent was removed under reduced pressure and the dark red residue was purified by column chromatography on silica gel (light petroleum/ethyl acetate 9:1–4:1) to give **3-CO-^{CF3}PhI** as a dark red solid in 22% yield (339 mg, 0.70 mmol, 0.22 eq.).[2] **^{1}H-NMR** (400 MHz, $CDCl_3$) δ [ppm] = 8.16 (br s, 1H, H-11), 7.70 (d, $^3J_{HH}$ = 7.7 Hz, 1H, H-8), 7.59 (d, $^3J_{HH}$ = 7.7 Hz, 1H, H-7), 4.71 (vt, $^3J_{HH}$ = 1.8 Hz, 2H, C-3), 4.65 (vt, $^3J_{HH}$ = 1.8 Hz, 2H, C-2), 4.31 (s, 5H, H-1). **^{19}F{^{1}H}-NMR** (376 MHz, $CDCl_3$) δ [ppm] = −62.9. **^{13}C{^{1}H}-NMR** (101 MHz, $CDCl_3$) δ [ppm] = 200.3 (C-5), 148.2 (C-6), 137.0 (q, $^3J_{CF}$ = 4 Hz, C-11), 132.8 (q, $^2J_{CF}$ = 33 Hz, C-9), 128.4 (C-7), 124.7 (q, $^3J_{CF}$ = 4 Hz, C-8), 122.7 (q, $^1J_{CF}$ = 273 Hz, C-10), 92.4 (C-12), 77.4 (C-4), 73.5 (C-2), 71.5(C-3), 70.4 (C-1). **HRMS (ESI/ion trap)** m/z: M^+ Calcd for $C_{18}H_{12}FeIOF_3$ 483.9229; Found 483.9222.

*Methoxy-ferroceno[2,3]-inden-1-one (*1'*)*

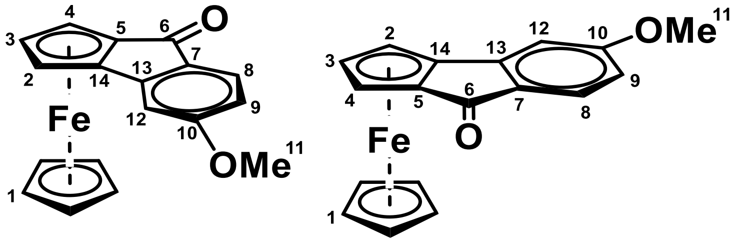

1-CO-^{MeO}PhI (197 mg, 0.44 mmol, 1.0 eq.), Cs_2CO_3 (293 mg, 0.90 mmol, 2.0 eq.), $Pd(OAc)_2$ (10 mg, 45 µmol, 0.10 eq.) and *rac*-BINAP (36 mg, 58 µmol, 0.13 eq.) were suspended in dry degassed toluene (15 mL). The mixture was heated to 100 °C (oil bath) for 20 hours. Then, the solvent was removed *in vacuo* and the remaining dark red residue was purified by gradient column chromatography on silica gel (*n*-pentane/ethyl acetate 10:1 – 2:1). **1'** was obtained as a dark red solid as a mixture of indistinguishable *Rp* and *Sp* enantiomers in 68% yield (96 mg, 0.30 mmol, 0.68 eq.).[2] **^{1}H-NMR** (400 MHz, $CDCl_3$) δ [ppm] = 7.44 (d, $^3J_{HH}$ = 8.3 Hz, 1H, H-8), 6.71 (d, $^4J_{HH}$ = 2.3 Hz, 1H, H-12), 6.57 (dd, $^3J_{HH}$ = 8.3 Hz, $^4J_{HH}$ = 2.3 Hz, 1H, H-9), 4.93 (d, $^3J_{HH}$ = 2.4 Hz, 1H, H-2), 4.82 (d, $^3J_{HH}$ = 2.4 Hz, 1H, H-4), 4.79 (vt, $^3J_{HH}$ = 2.4 Hz, 1H, H-3), 4.12 (s, 5H, H-1), 3.86 (s, 3H, H-11). **^{13}C{^{1}H}-NMR** (101 MHz, $CDCl_3$) δ [ppm] = 194.2 (C-6), 164.4 (C-10), 146.1 (C-13), 133.4 (C-7), 124.8 (C-8), 110.5 (C-9), 107.4 (C-12), 89.2 (C-14), 79.4 (C-5), 74.9 (C-3), 73.0 (C-1), 66.3 (C-2), 66.1 (C-4), 55.7 (C-11). **HRMS (ESI/ion trap)** m/z: $[M+Na]^+$ Calcd for $C_{18}H_{14}FeO_2Na$ 341.0235; Found 341.0238.

*Methyl-ferroceno[2,3]-inden-1-one (*2'*)*

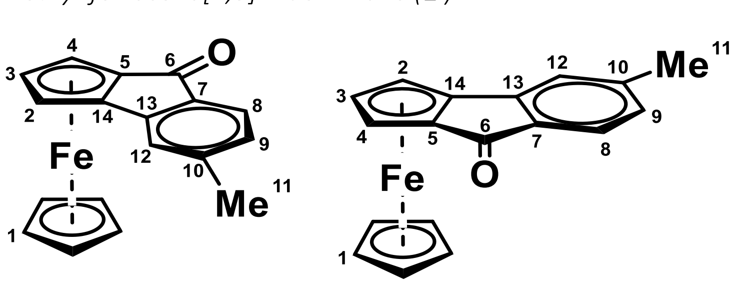

2-CO-^{Me}PhI (1.0 g, 2.33 mmol, 1.0 eq.), Cs_2CO_3 (1.52 g, 4.66 mmol, 2.00 eq.), $Pd(OAc)_2$ (30 mg, 0.12 mmol, 0.05 eq.) and *rac*-BINAP (90 mg, 0.15 mmol, 0.07 eq.) were suspended in dry degassed toluene (100 mL) and heated to 100 °C for 20 hours with the aid of an aluminum heating block. The solvent was removed *in vacuo* and the remaining dark red residue was purified by column chromatography on silica gel (*n*-pentane/ethyl acetate 4:1). **2'** was obtained as a racemic mixture of *Rp* and *Sp* enantiomers as a dark red solid in 83% yield

(581 mg, 1.92 mmol, 0.83 eq.).[2] **^{1}H-NMR** (400 MHz, $CDCl_3$) δ [ppm] = 7.38 (d, $^3J_{HH}$ = 7.4 Hz, 1H, H-8), 7.00 (s, 1H, H-12), 6.90 (d, $^3J_{HH}$ = 7.4 Hz, 1H, H-9), 4.95 (s, 1H, H-4), 4.83 (s, 2H, H-2/3), 4.12 (s, 5H, H-1), 2.33 (s, 3H, H-11). **^{13}C{^{1}H}-NMR** (101 MHz, $CDCl_3$) δ [ppm] = 195.3 (C-6), 169.2 (C-7), 145.7 (C-10), 144.1 (C-13), 127.3 (C-9), 123.0 (C-12), 121.4 (C-8), 90.3 (C-14), 78.9 (C-5), 75.2 (C-2/4), 73.0 (C-1), 66.3 (C-4), 66.2 (C-2/3), 22.2 (C-11). **HRMS (ESI/ion trap)** m/z: $[M+H]^+$ Calcd for $C_{18}H_{15}FeO$ 303.0467; Found 303.0466.

*Trifluoromethyl-ferroceno[2,3]-inden-1-one (*3'*)*

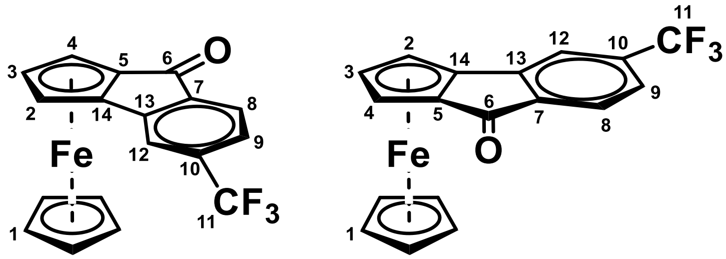

3-CO-^{CF3}PhI (253 mg, 523 µmol, 1.00 eq.), Cs_2CO_3 (341 mg, 1.05 mmol, 2.00 eq.), $Pd(OAc)_2$ (18 mg, 78 µmol, 0.15 eq.) and *rac*-BINAP (65 mg, 0.10 mmol, 0.20 eq.) were suspended in dry degassed toluene (100 mL) and heated in an oil bath to 100 °C for 20 hours. The solvent was removed *in vacuo* and the remaining dark red residue was purified by gradient column chromatography on silica gel (*n*-pentane/ethyl acetate 1:0 – 1:4). **3'** was obtained as a racemic mixture of *Rp* and *Sp* enantiomers as a violet solid in 49% yield (92 mg, 258 µmol, 0.49 eq.).[2] **^{1}H-NMR** (400 MHz, $CDCl_3$) δ [ppm] = 7.58 (d, $^3J_{HH}$ = 7.7 Hz, 1H, H-8), 7.40 (d, $^3J_{HH}$ = 7.7 Hz, 1H, H-9), 7.39 (s, 1H, H-12), 5.08 (s, 1H, H-3), 4.98 (s, 1H, H-2/H-4), 4.97 (s, 1H, H-2/H-4), 4.17 (s, 5H, H-1). **^{19}F{^{1}H}-NMR** (376 MHz, $CDCl_3$) δ [ppm] = −63.2. **^{13}C{^{1}H}-NMR** (101 MHz, $CDCl_3$) δ [ppm] = 193.6 (C-6), 144.4 (C-13/C-10), 143.3 (C-7), 134.8 (C-13/C-10), 123.8 (C-9), 123.2 (C-8), 116.9 (C-12), 89.1 (C-14), 78.7 (C-5), 76.4 (C-2/C-4), 73.4 (C-1), 67.3 (C-2/C-4), 67.1 (C-3). **HRMS (ESI/ion trap)** m/z: M^+ Calcd for $C_{18}H_{12}F_3FeIO$ 483.9229; Found 483.9225.

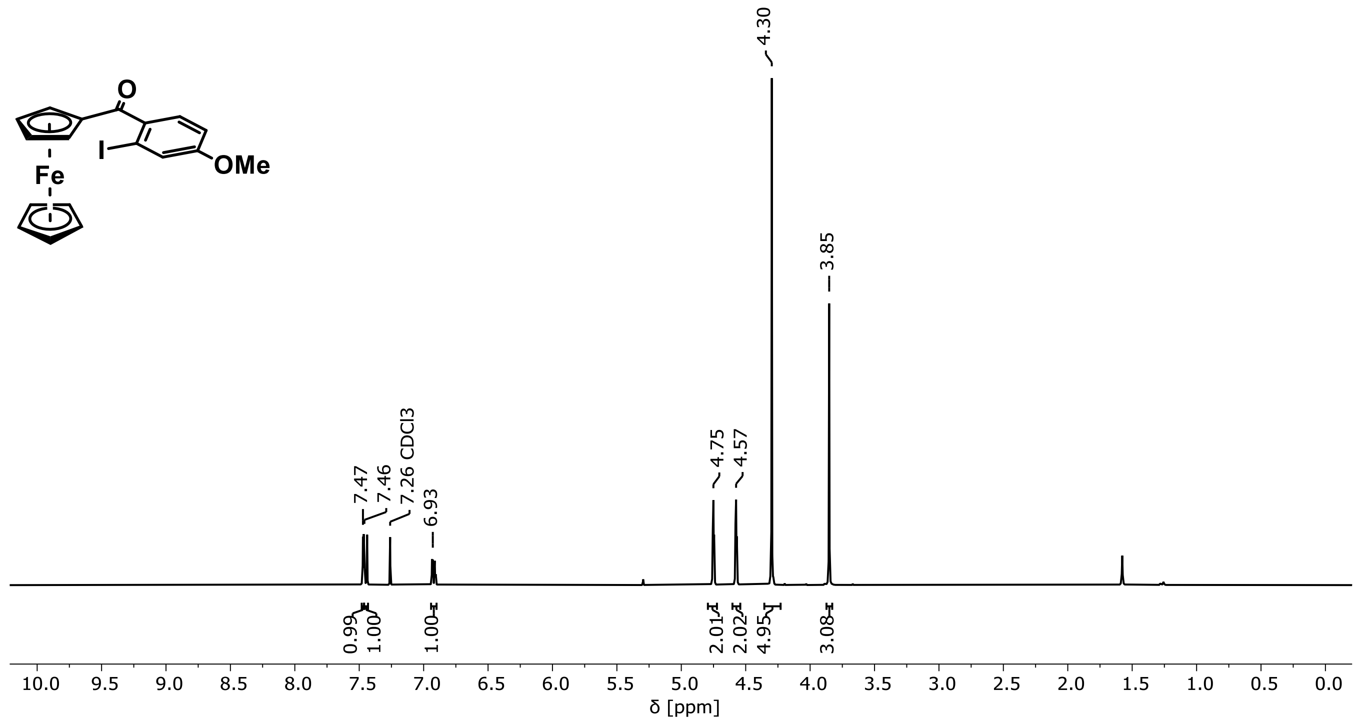

Figure S1: ^{1}H-NMR (400 MHz, $CDCl_3$) spectrum of **1-CO^{OMe}PhI**.

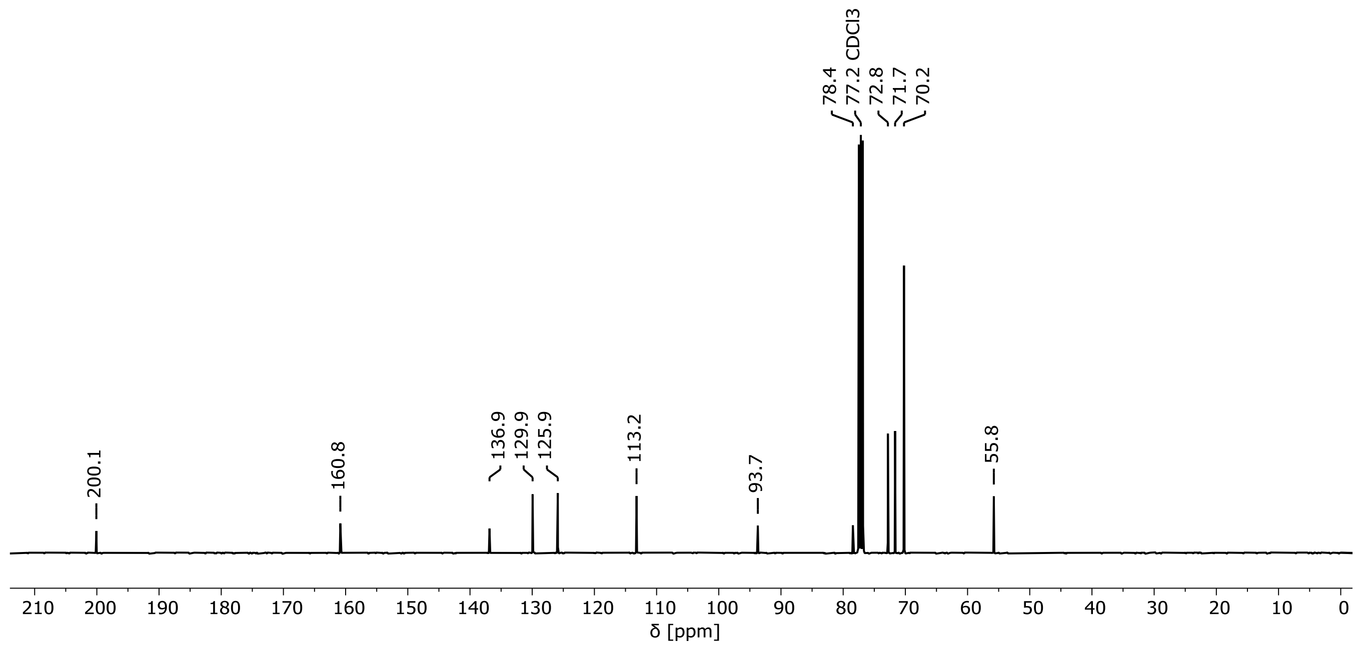

Figure S2: ^{13}C{^{1}H}-NMR (101 MHz, $CDCl_3$) spectrum of **1-CO^{OMe}PhI**.

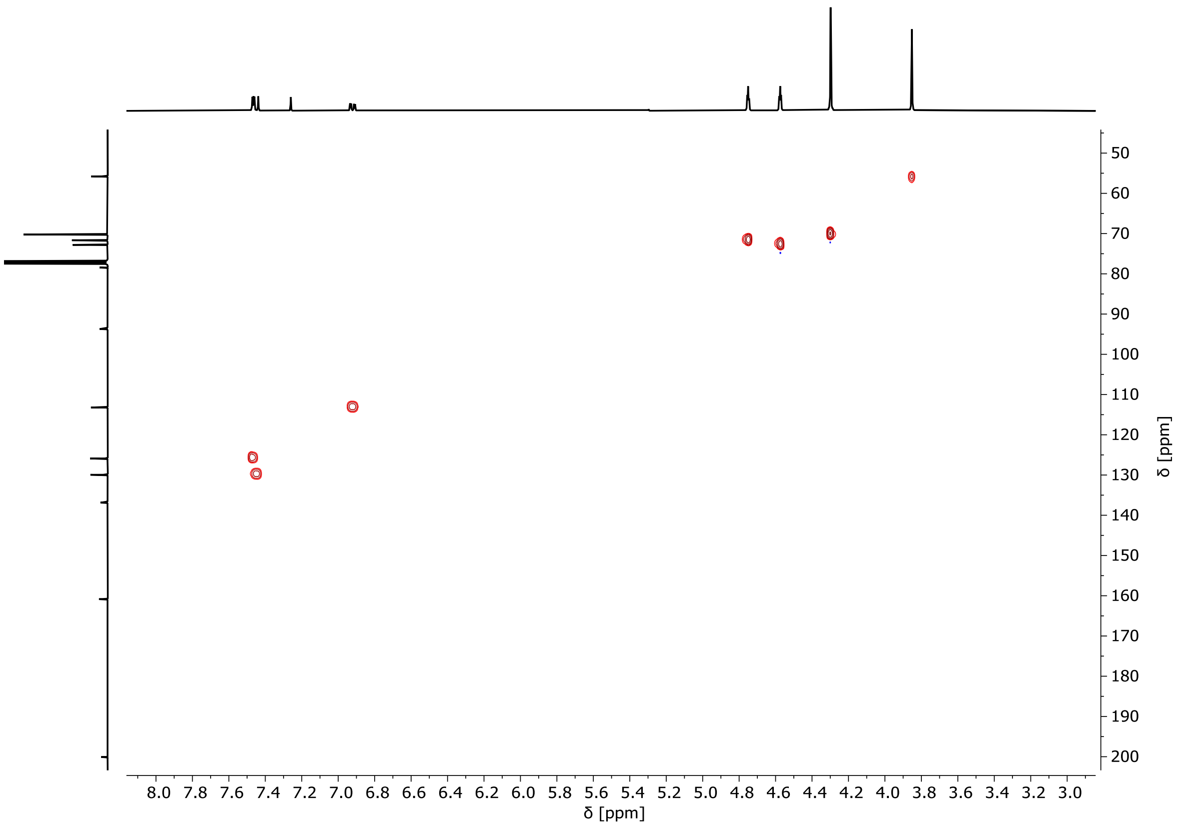

Figure S3: HSQC NMR spectrum of **1-CO^{OMe}PhI**.

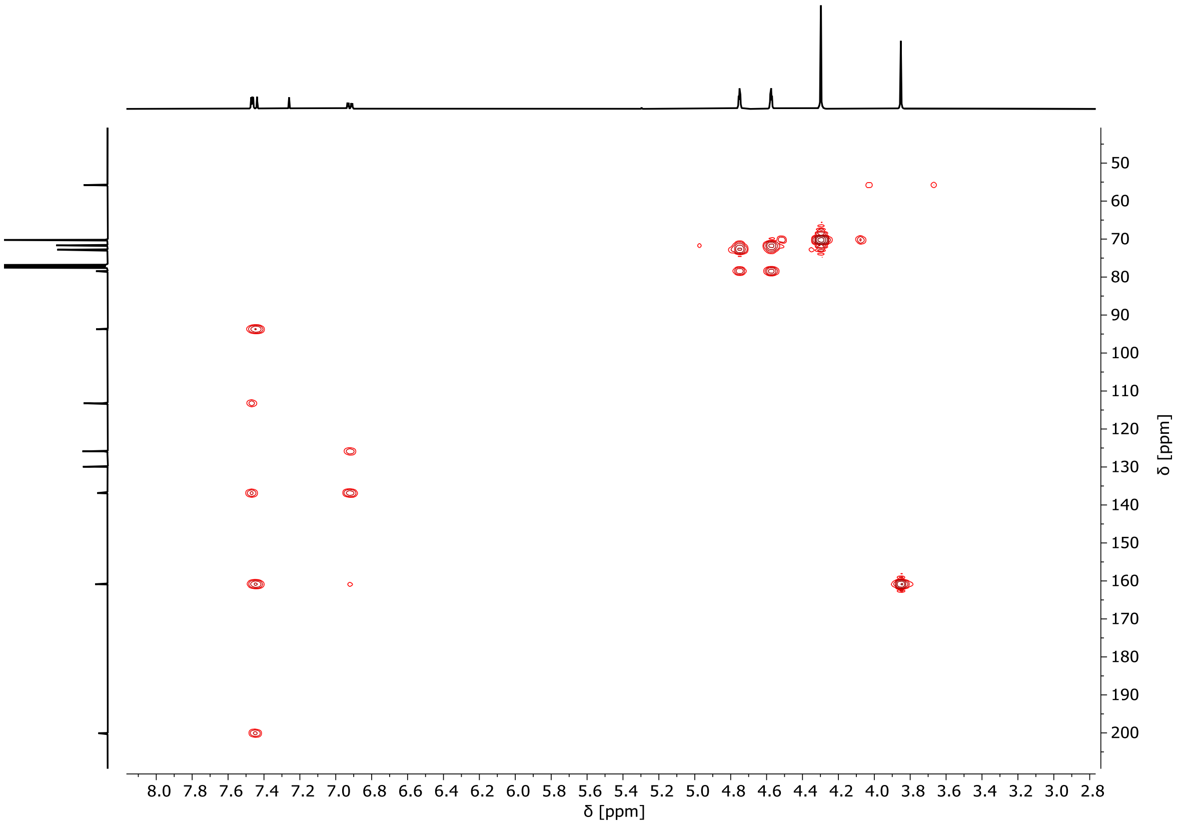

Figure S4: HMBC NMR spectrum of **1-CO^{OMe}PhI**.

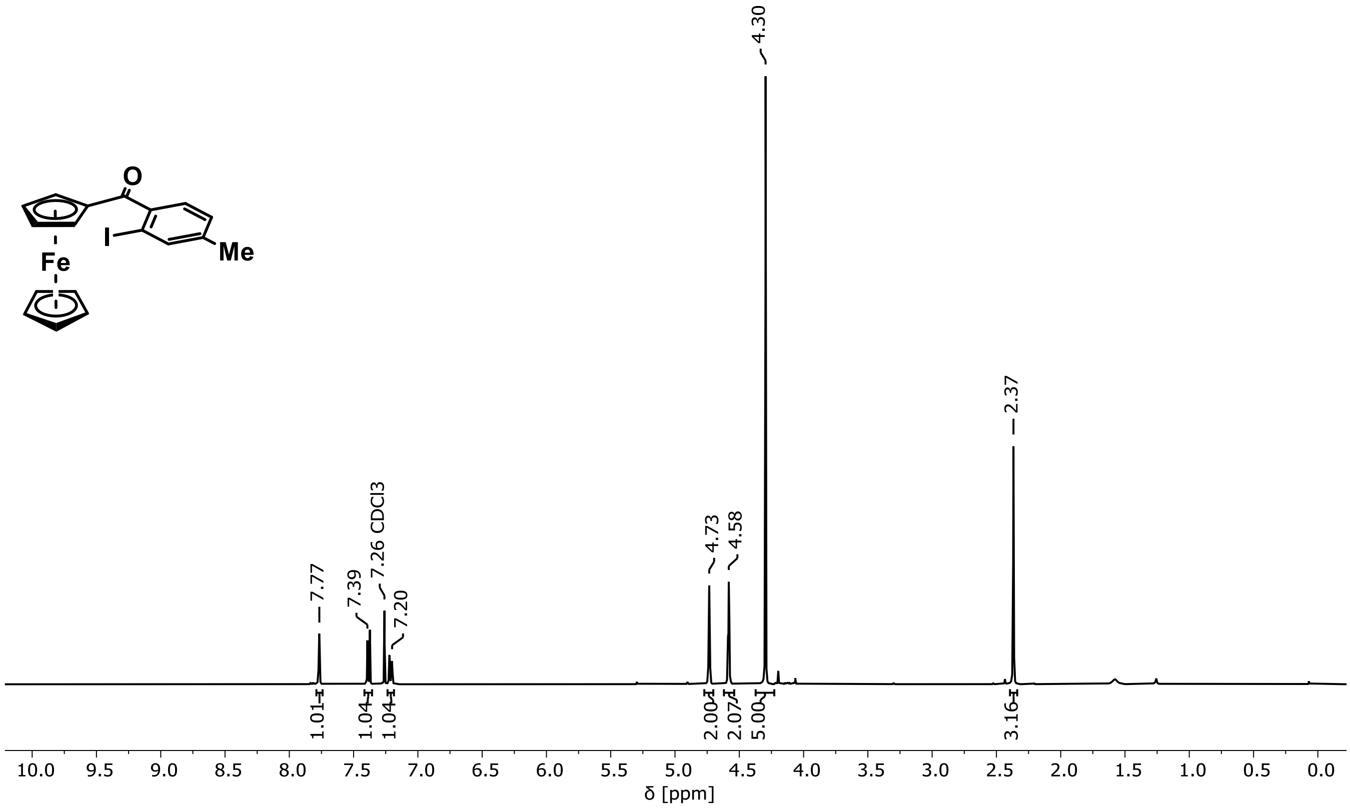

Figure S5: ^{1}H-NMR (400 MHz, $CDCl_3$) spectrum of **2-CO^{Me}PhI**.

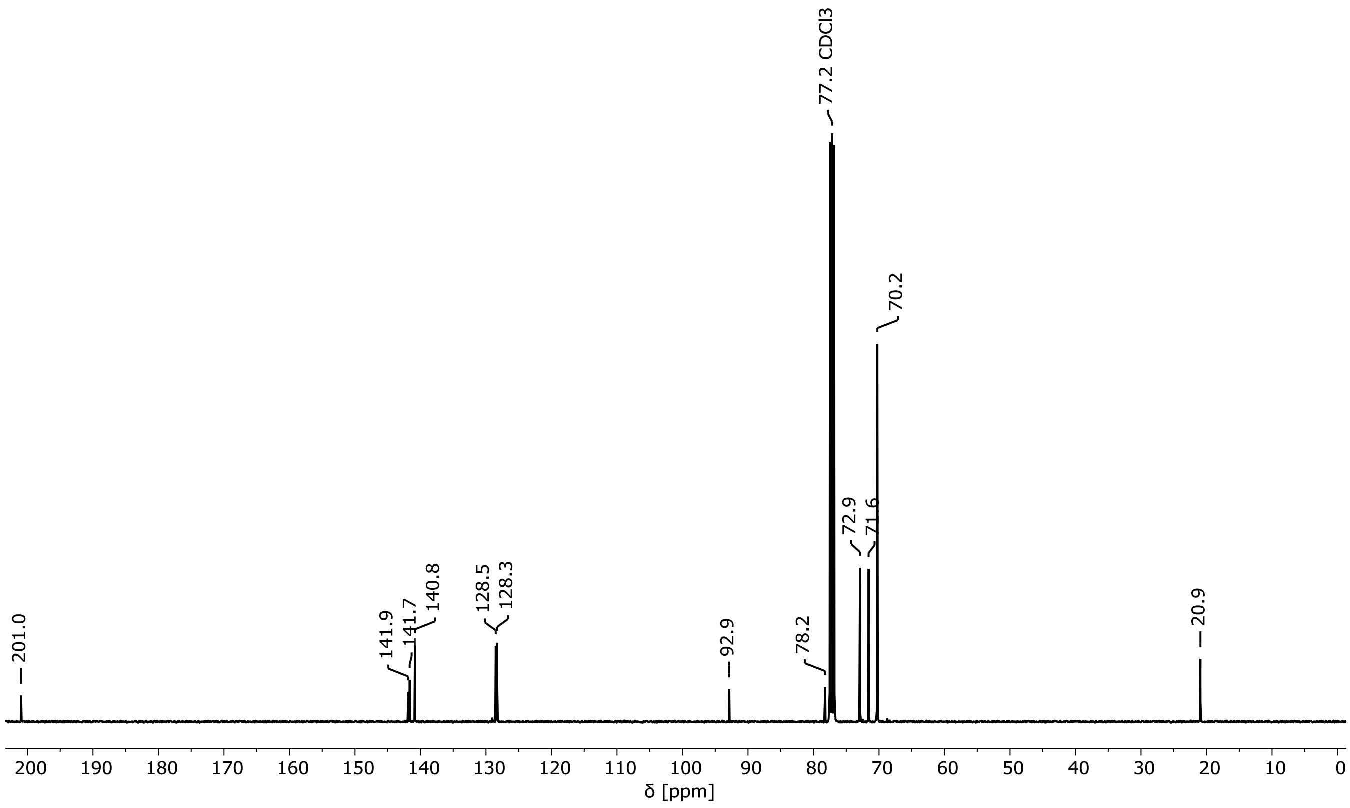

Figure S6: $^{13}C\{^{1}H\}$-NMR (101 MHz, $CDCl_3$) spectrum of **2-CO^{Me}PhI**.

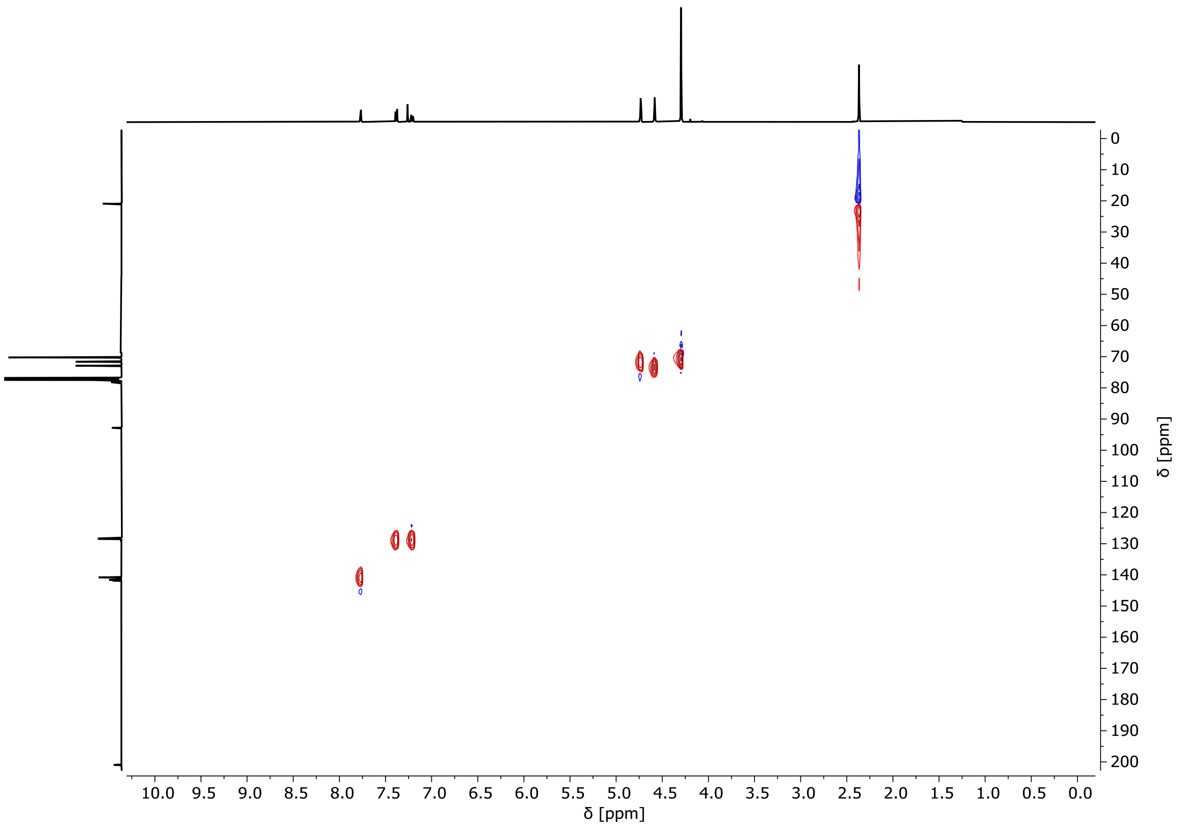

Figure S7: HSQC NMR spectrum of **2-CO^{Me}PhI**.

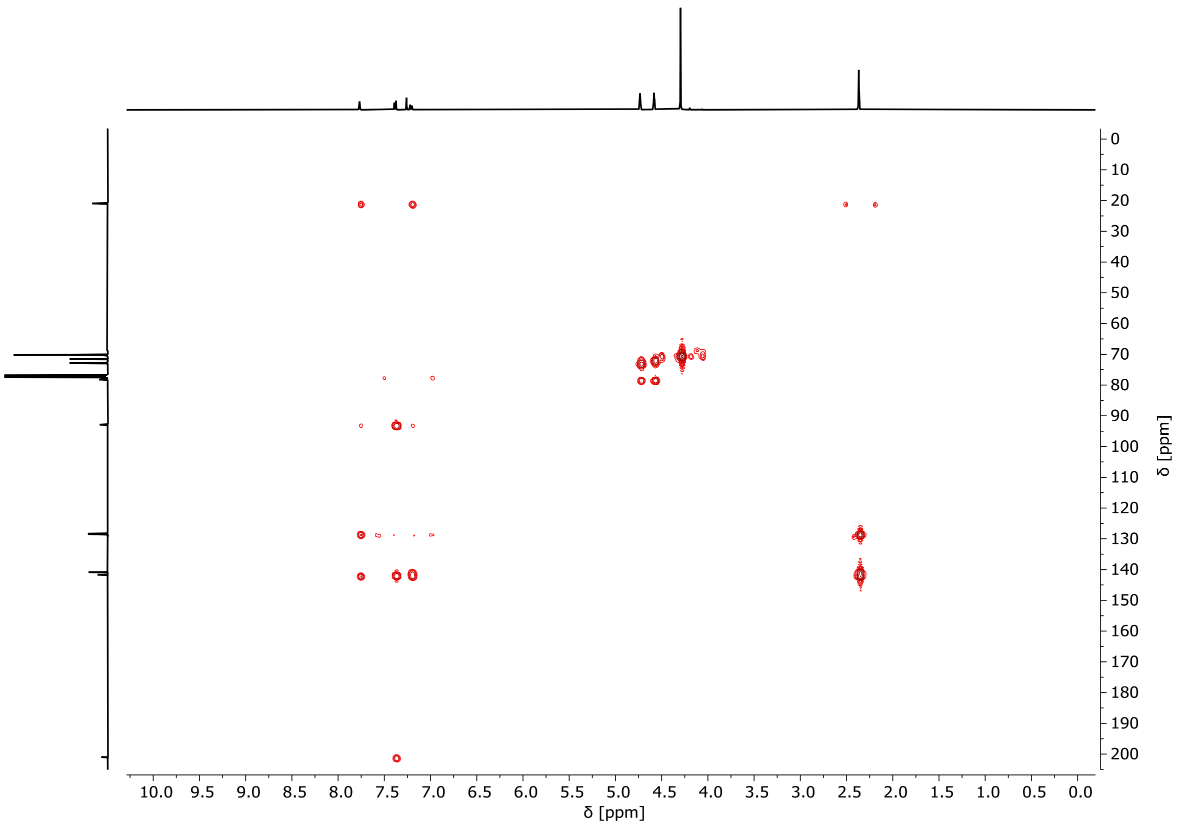

Figure S8: HMBC NMR spectrum of **2-CO^{Me}PhI**.

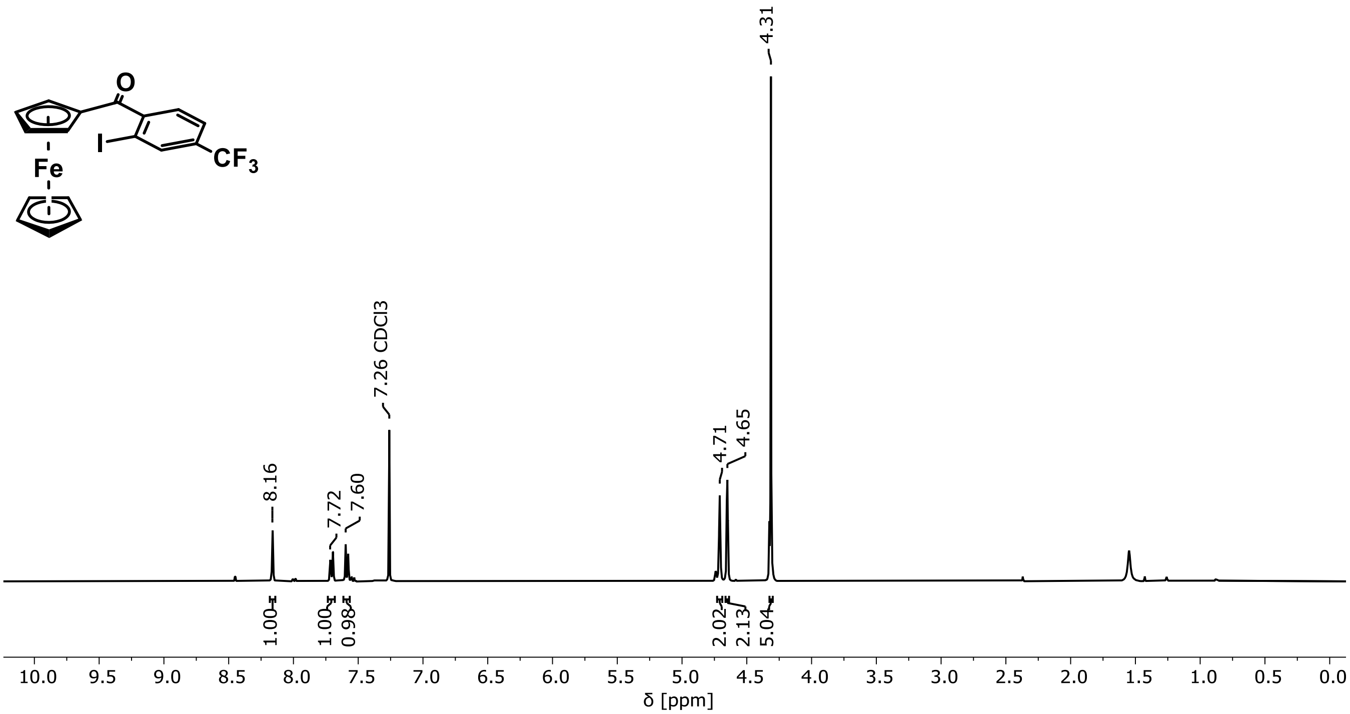

Figure S9: ^{1}H-NMR (400 MHz, $CDCl_3$) spectrum of **3-CO^{CF3}PhI**.

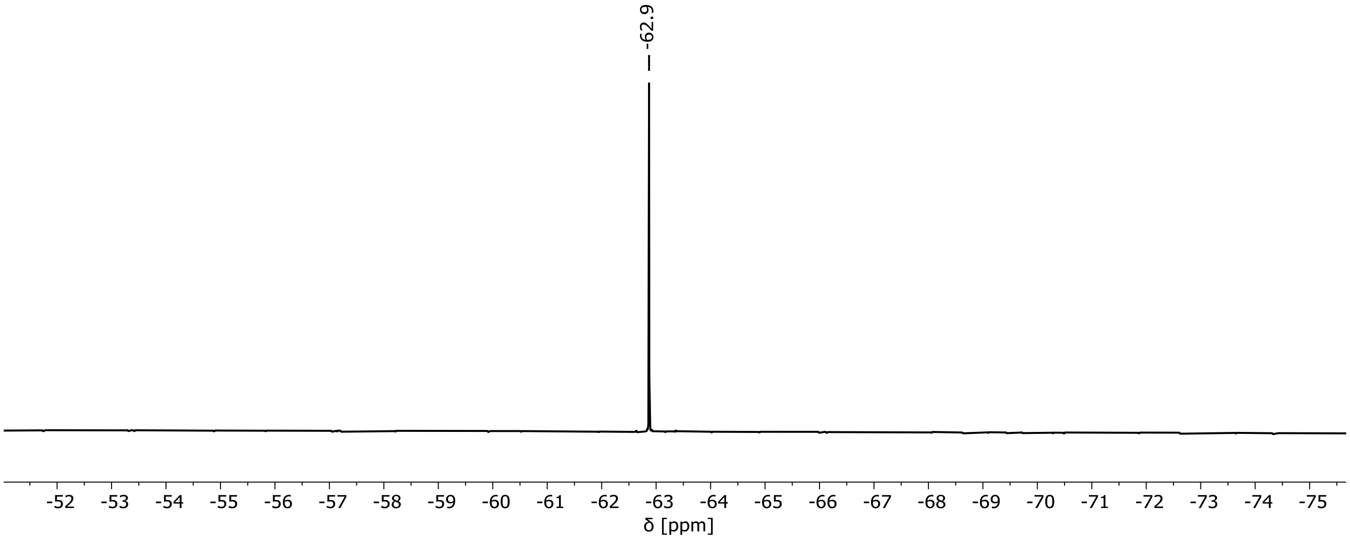

Figure S10: ^{19}F{^{1}H}-NMR (376 MHz, $CDCl_3$) spectrum of **3-CO^{CF3}PhI**.

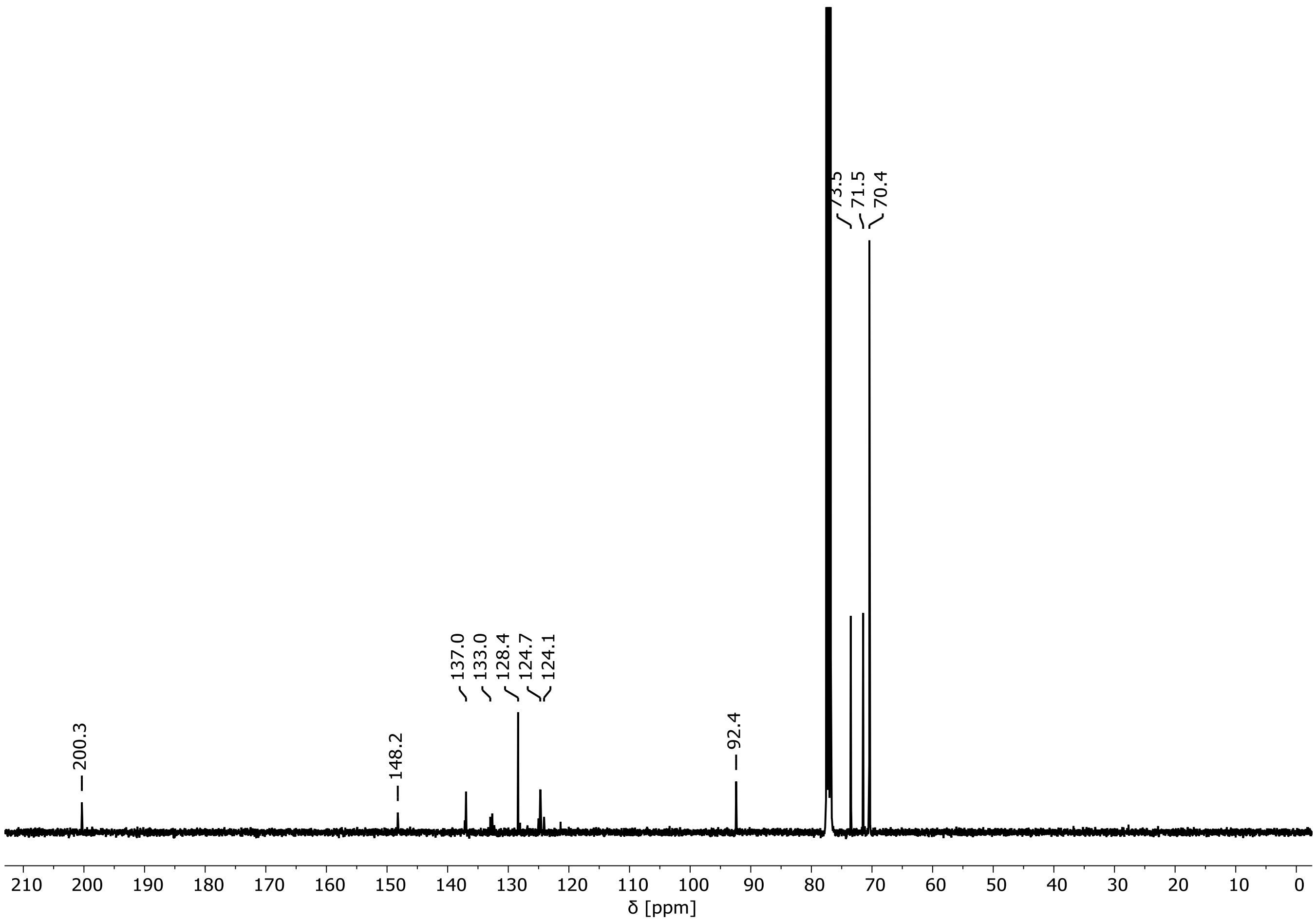

Figure S11: $^{13}C\{^{1}H\}$-NMR (101 MHz, $CDCl_3$) spectrum of **3-CO^{CF3}PhI**.

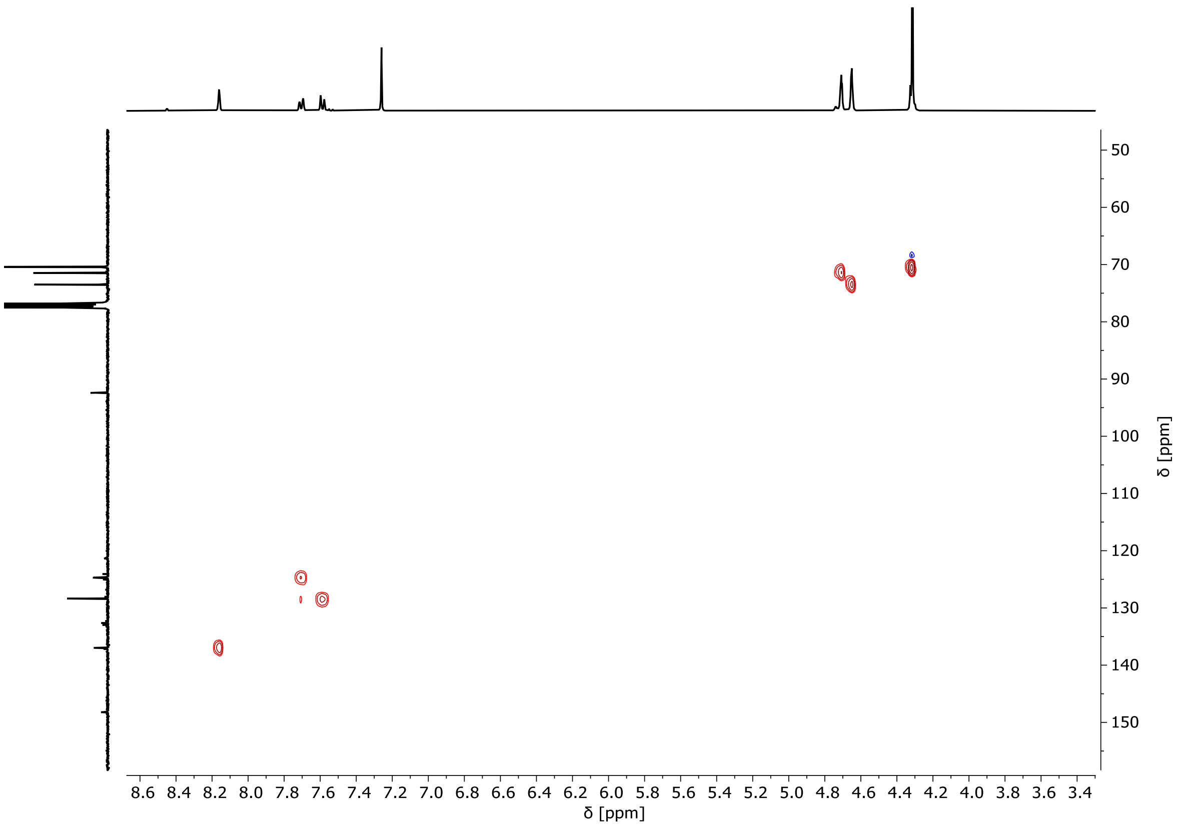

Figure S12: HSQC NMR spectrum of **3-CO^{CF3}PhI**.

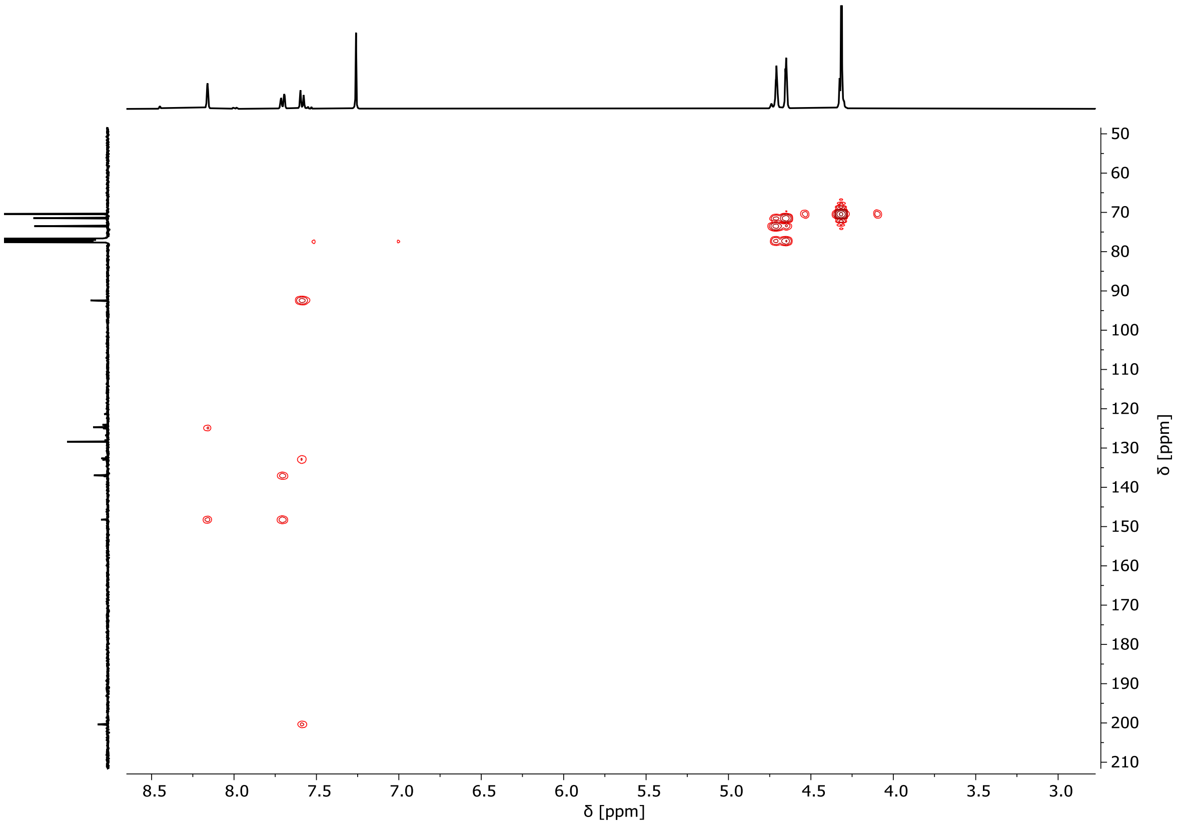

Figure S13: HMBC NMR spectrum of **3-CO^{CF3}PhI**.

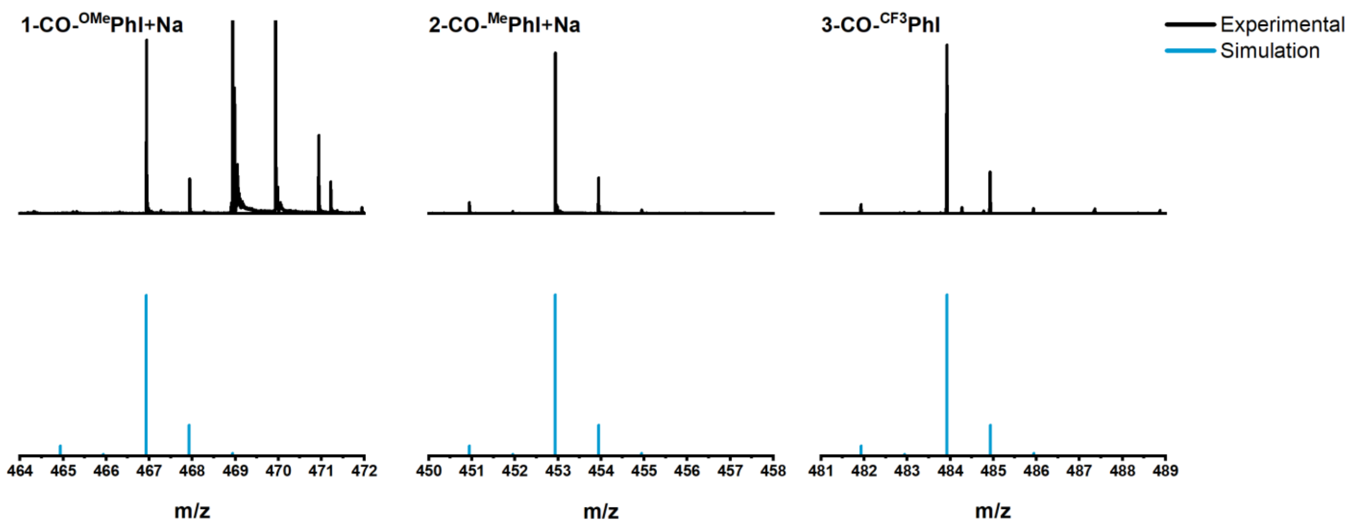

Figure S14: Experimental mass spectra of **1-CO^{OMe}PhI** – **3-CO^{CF3}PhI** (black lines) with the simulated spectra shown as blue lines at the bottom.

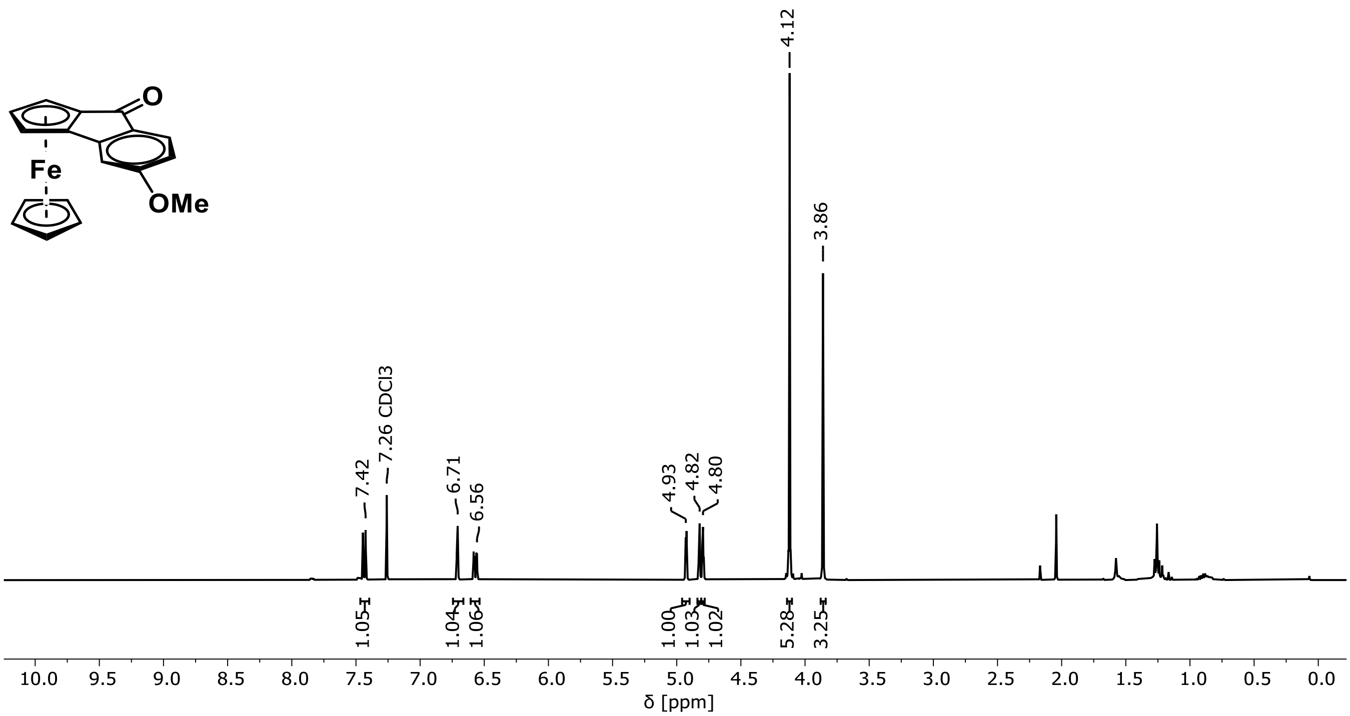

Figure S15: ^{1}H-NMR (400 MHz, $CDCl_3$) spectrum of ketone **1’**.

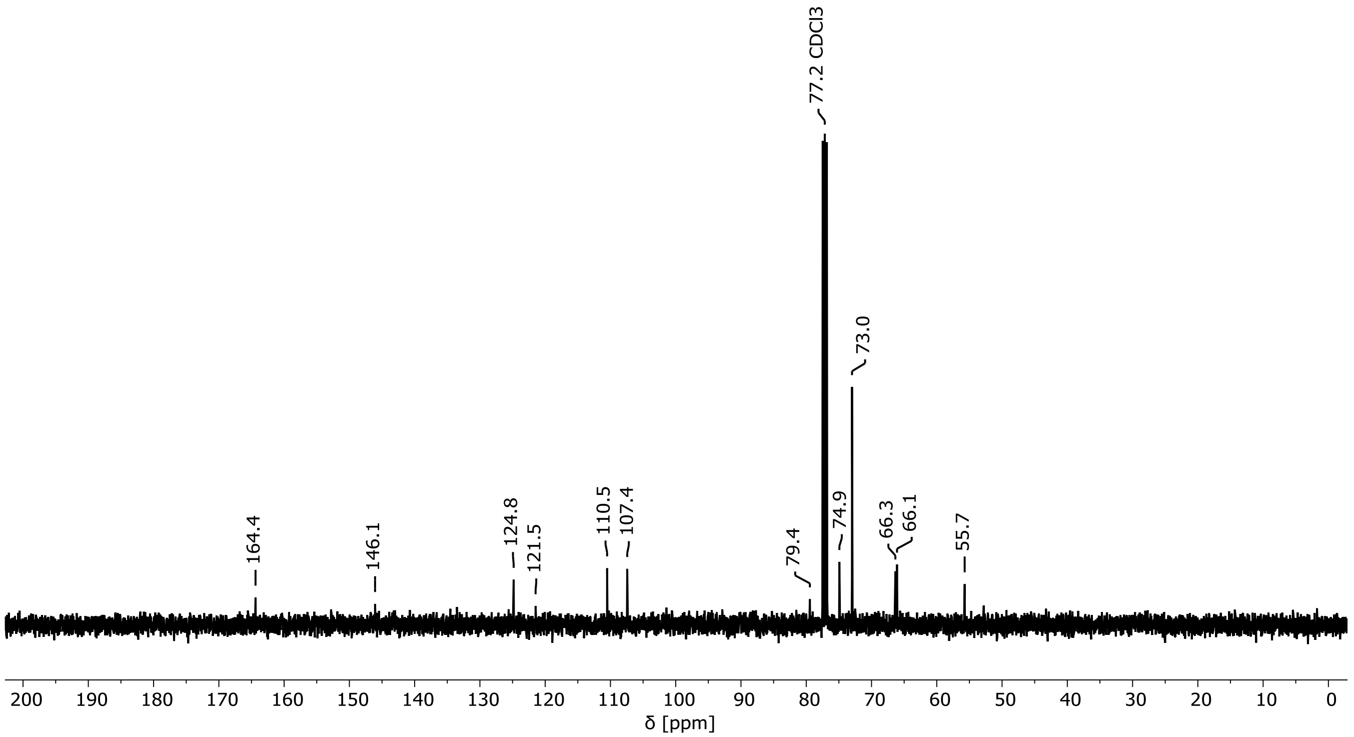

Figure S16: $^{13}C\{^{1}H\}$-NMR (101 MHz, $CDCl_3$) spectrum of ketone **1’**.

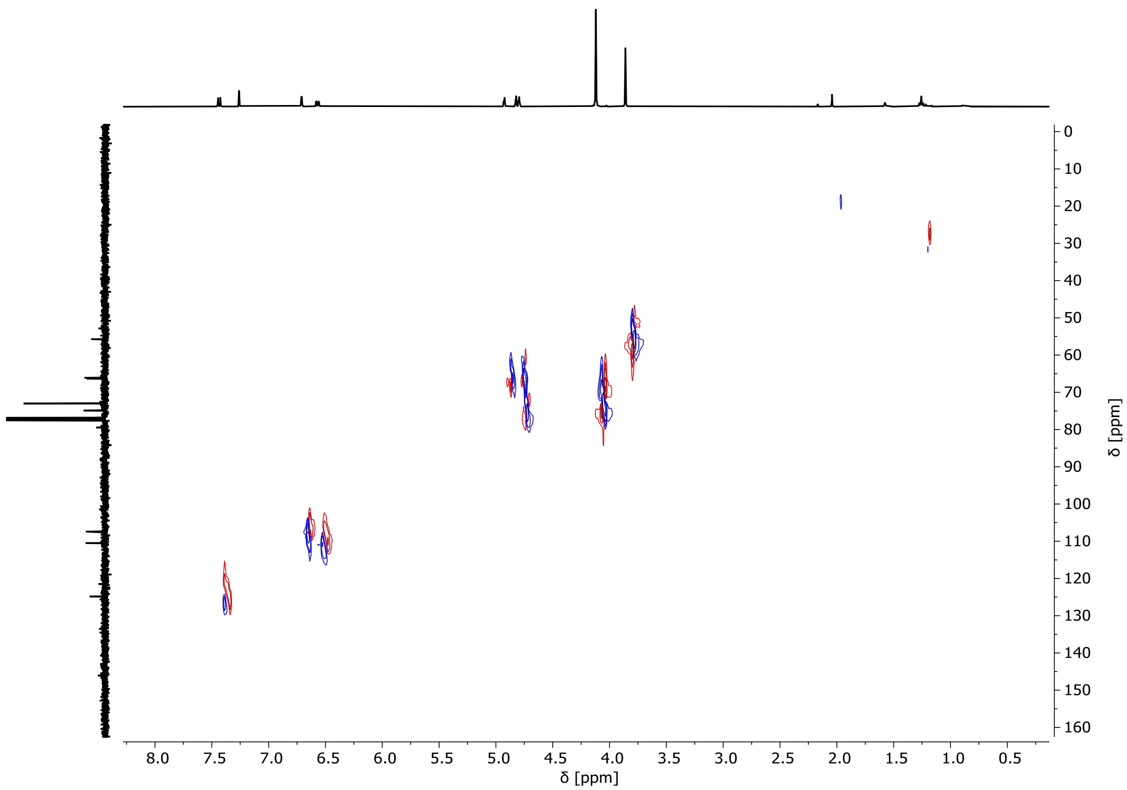

Figure S17: HSQC NMR spectrum of ketone **1’**.

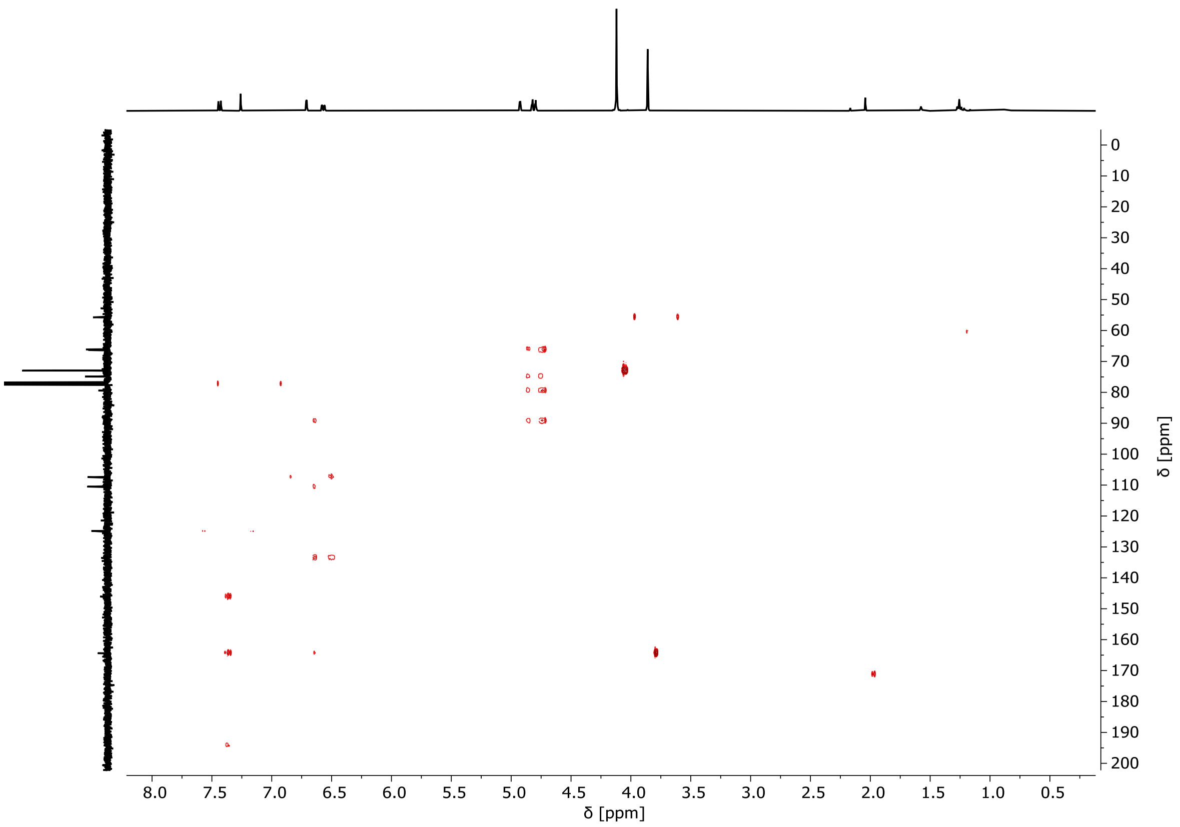

Figure S18: HMBC NMR spectrum of ketone **1’**.

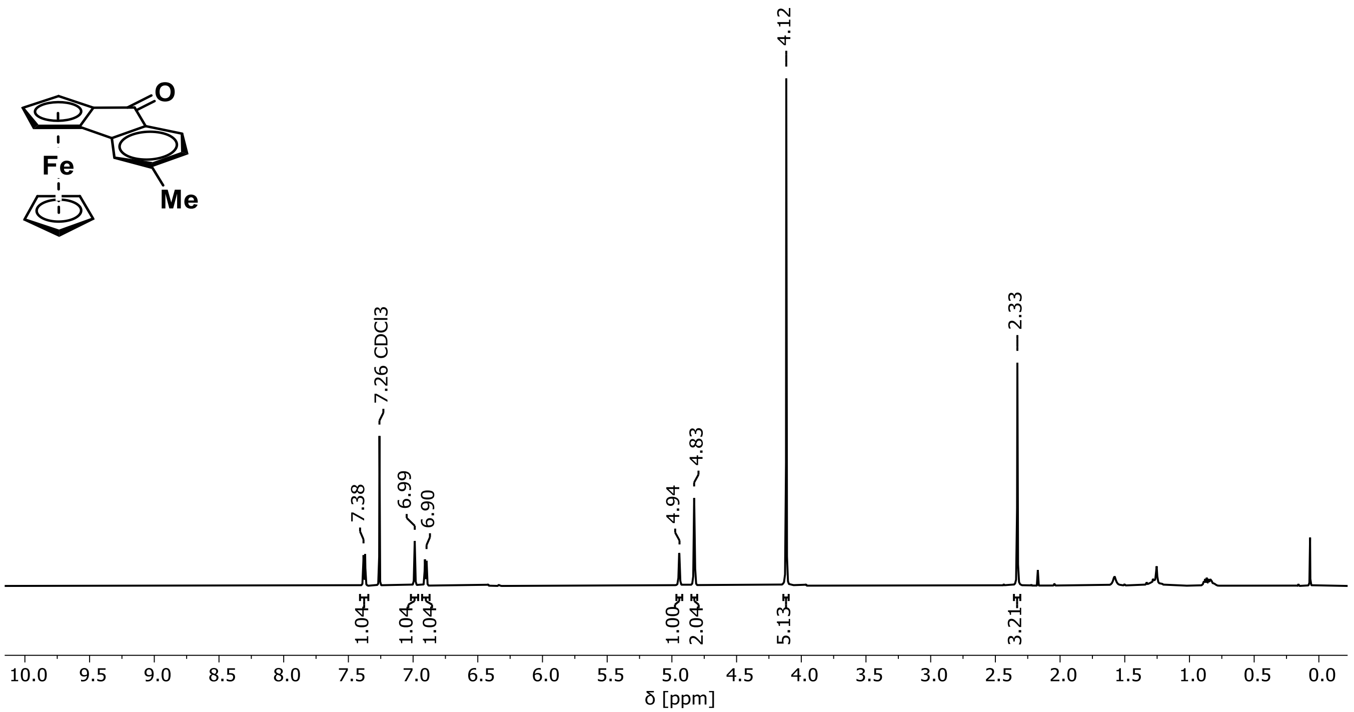

Figure S19: ^{1}H-NMR (400 MHz, $CDCl_3$) spectrum of ketone **2’**.

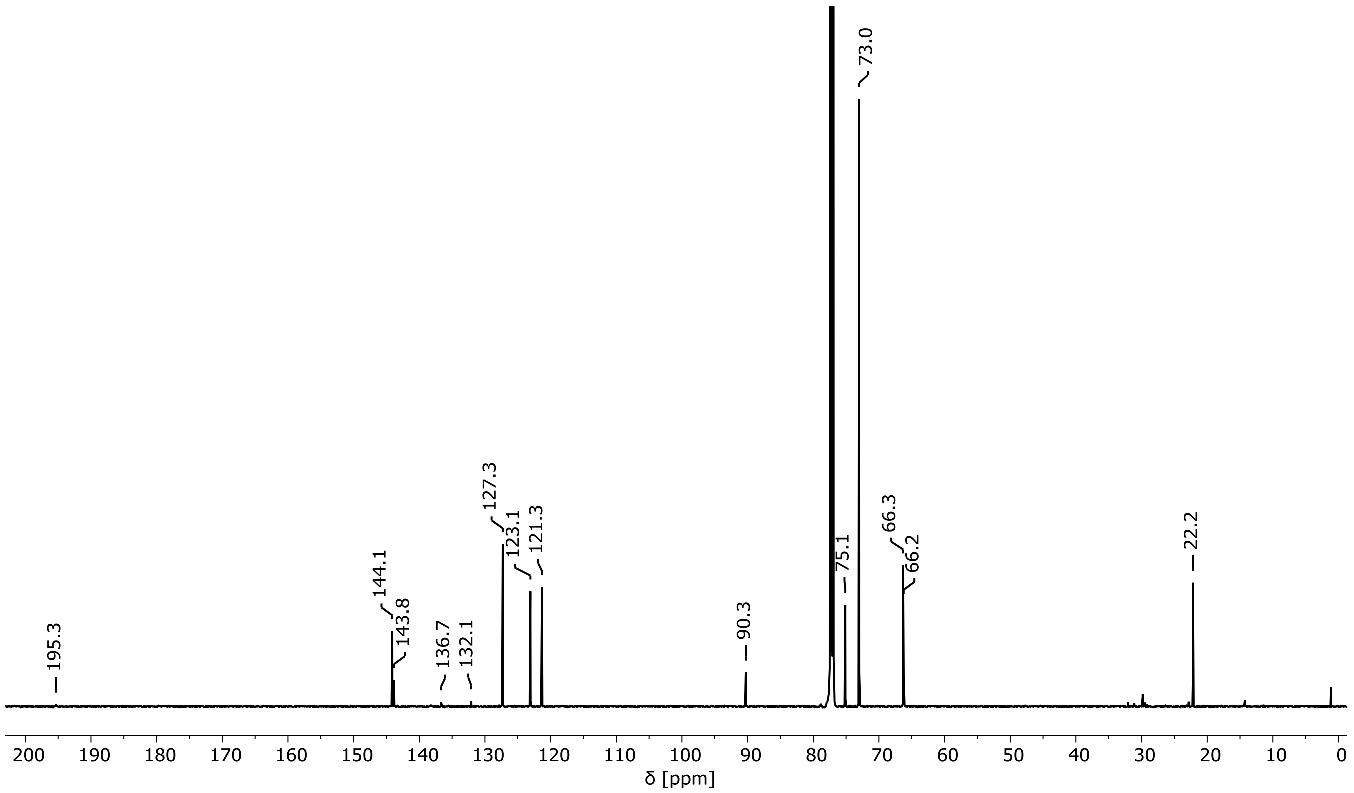

Figure S20: $^{13}C\{^{1}H\}$-NMR (101 MHz, $CDCl_3$) spectrum of ketone **2’**.

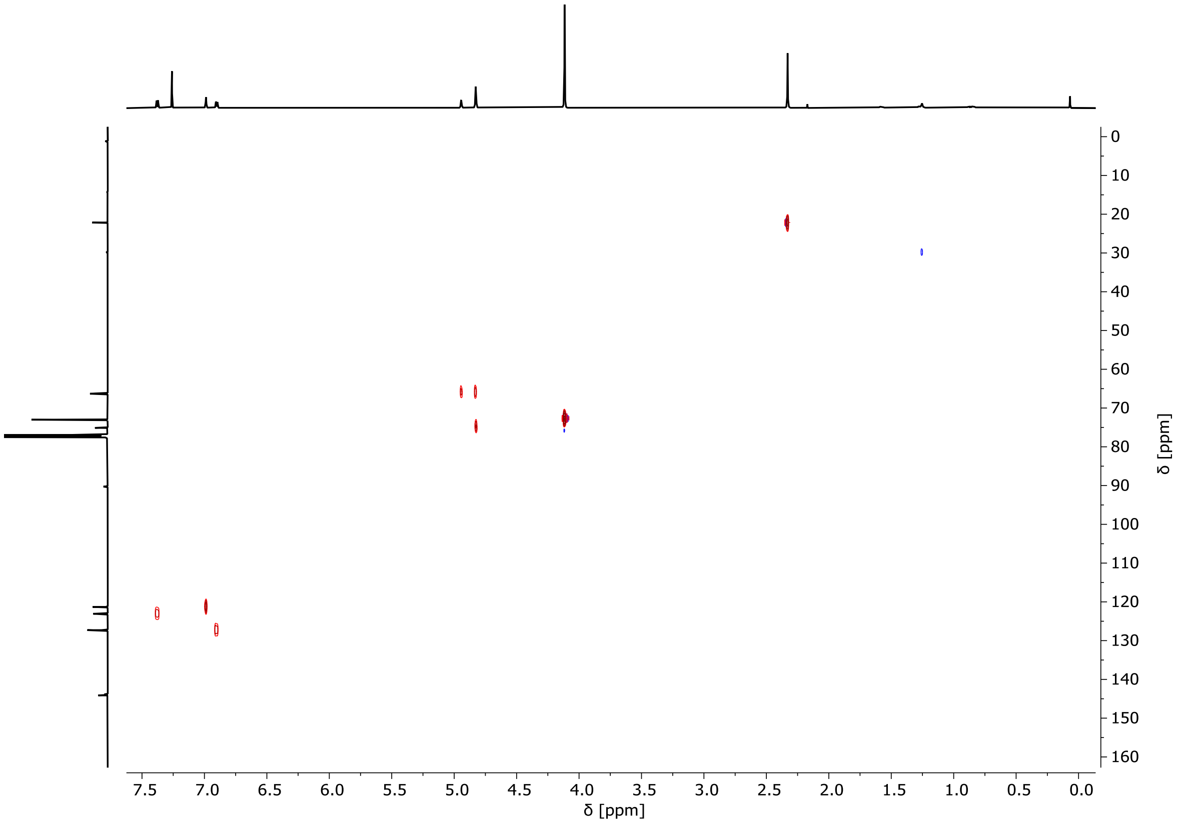

Figure S21: HSQC NMR spectrum of ketone **2’**.

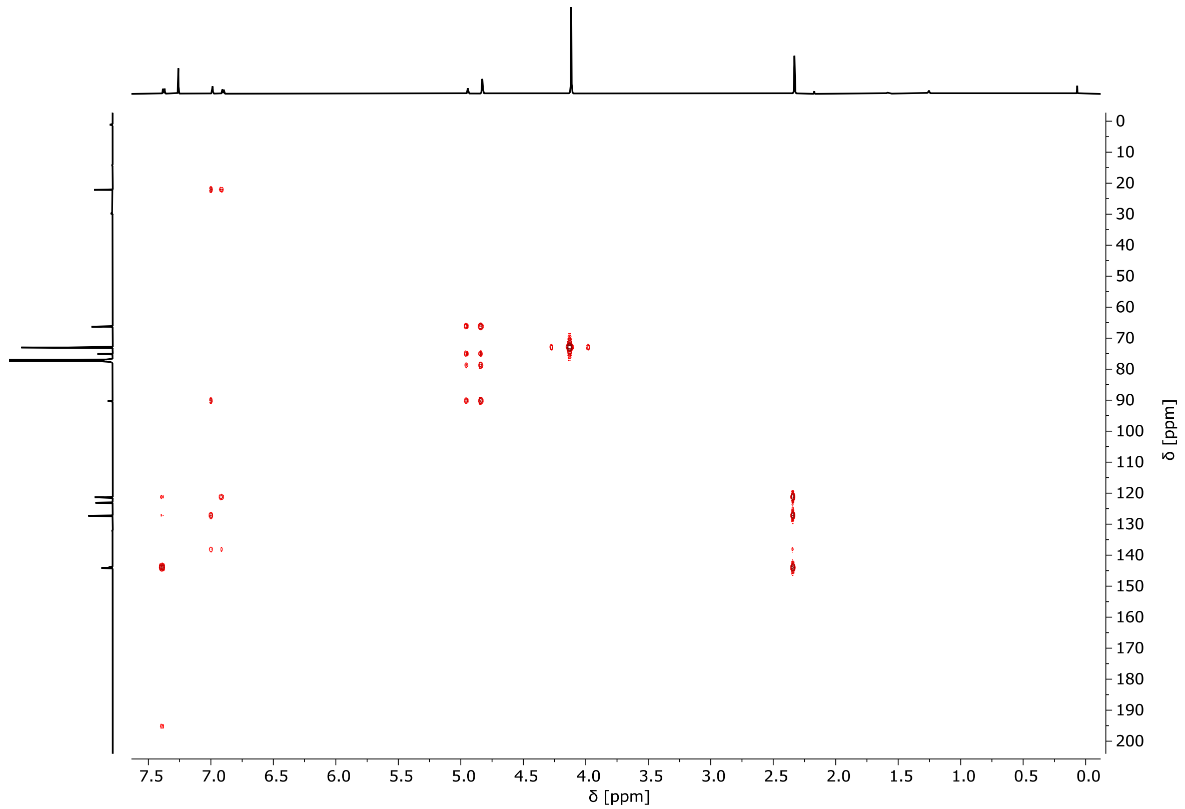

Figure S22: HMBC NMR spectrum of ketone **2’**.

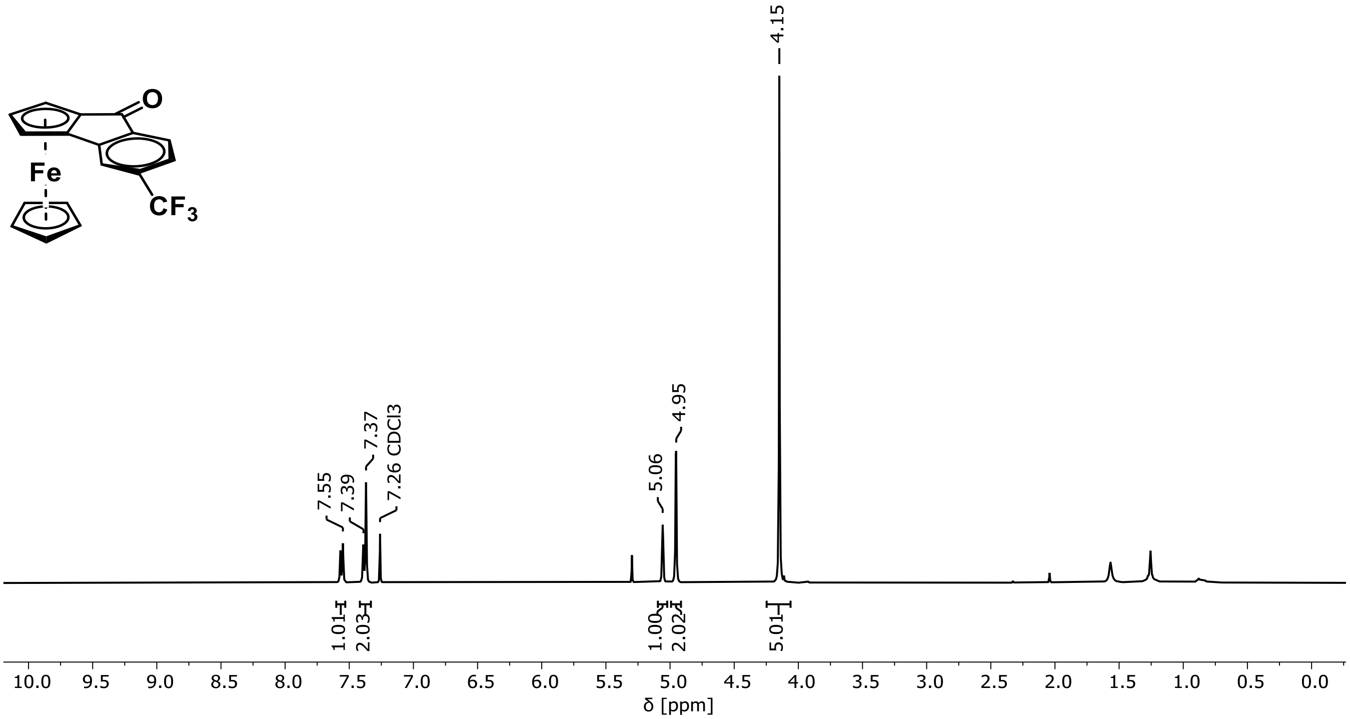

Figure S23: ^{1}H-NMR (400 MHz, $CDCl_3$) spectrum of ketone **3’**.

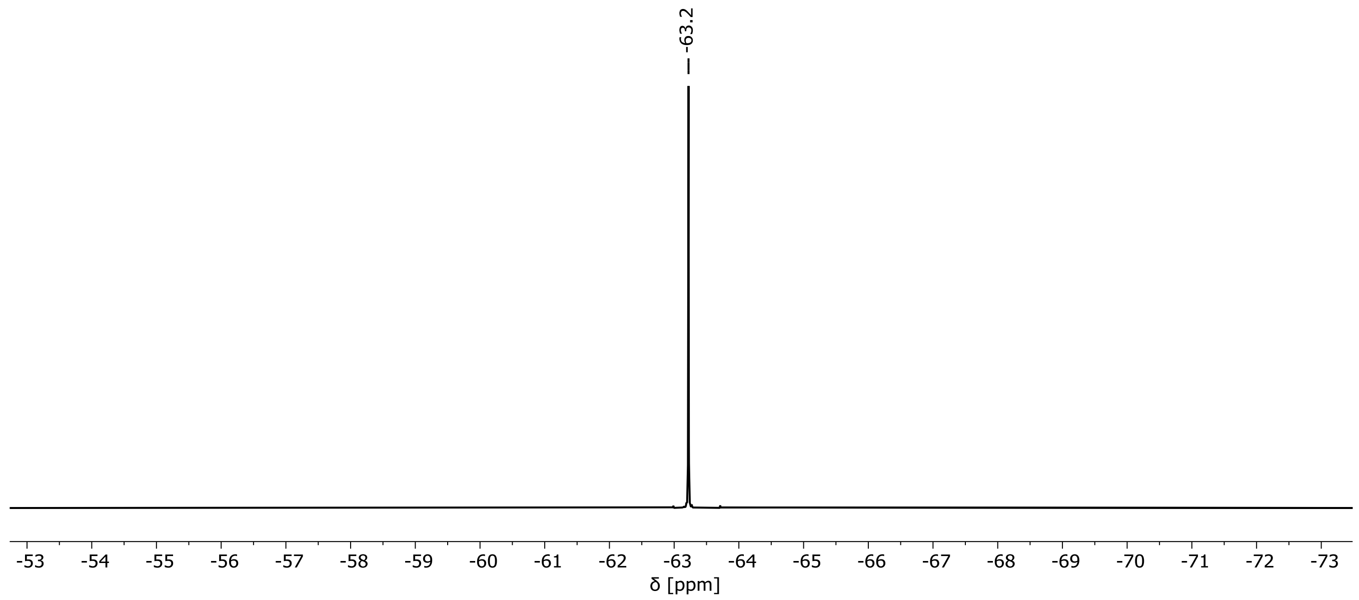

Figure S24: $^{19}F\{^{1}H\}$-NMR (376 MHz, $CDCl_3$) spectrum of ketone **3'**.

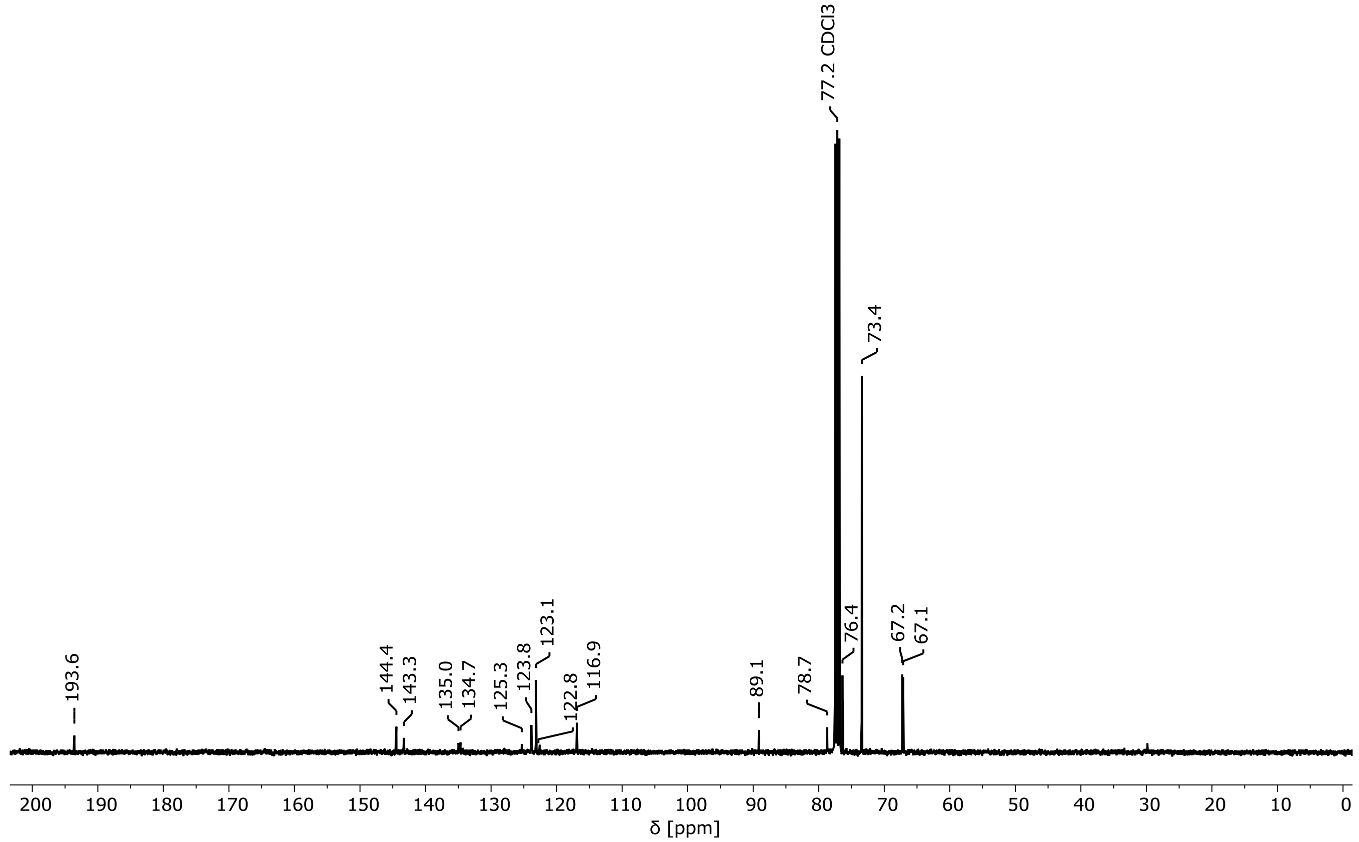

Figure S25: $^{13}C\{^{1}H\}$-NMR (101 MHz, $CDCl_3$) spectrum of ketone **3'**.

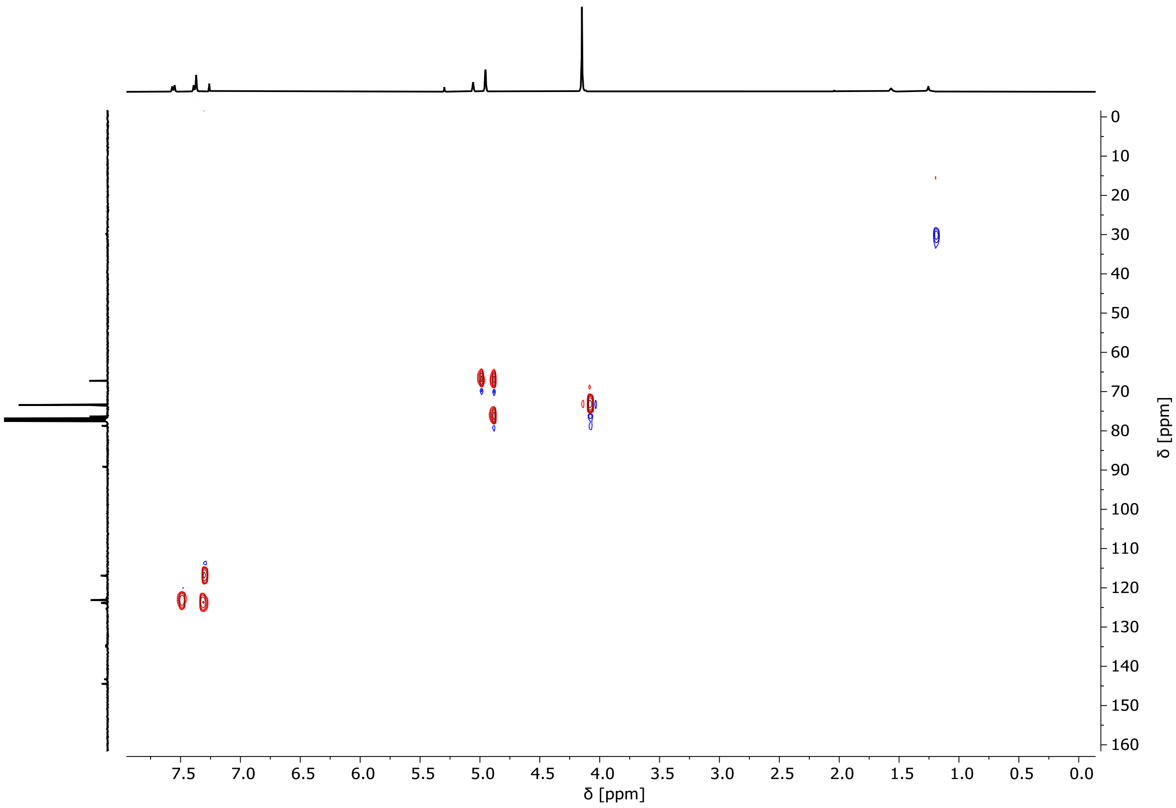

Figure S26: HSQC NMR spectrum of ketone **3’**.

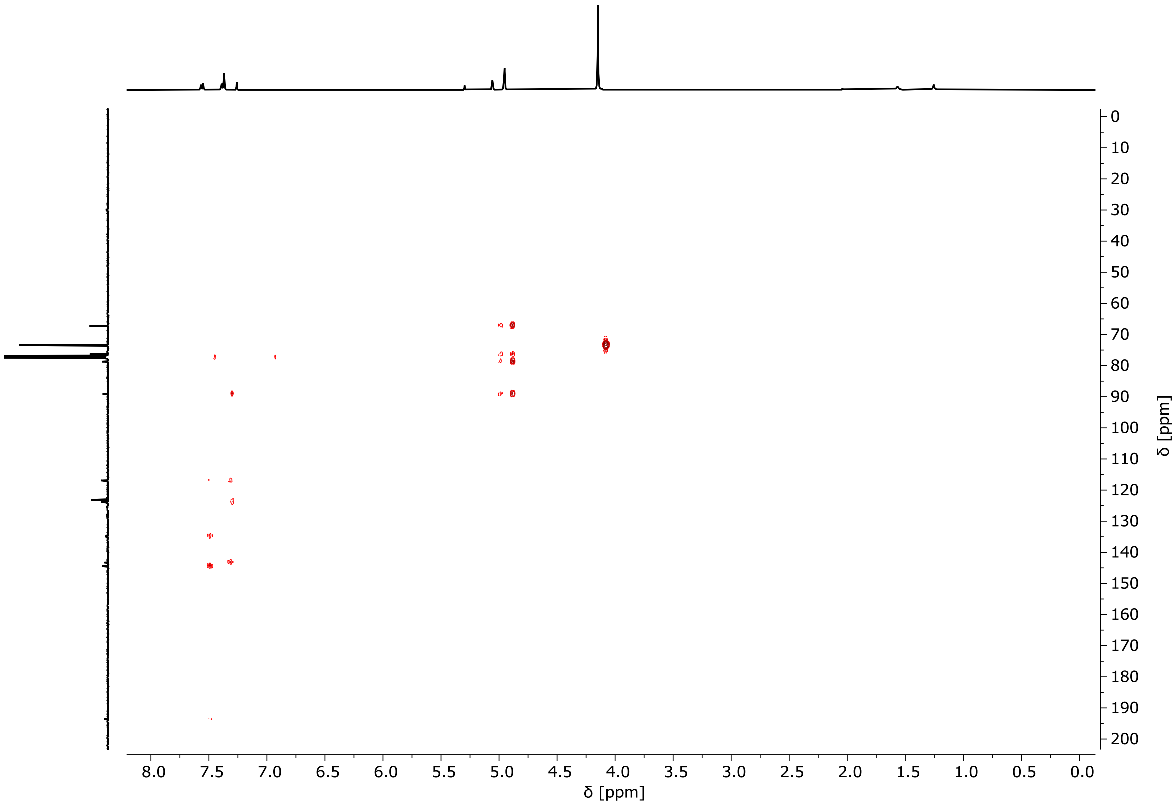

Figure S27: HMBC NMR spectrum of ketone **3’**.

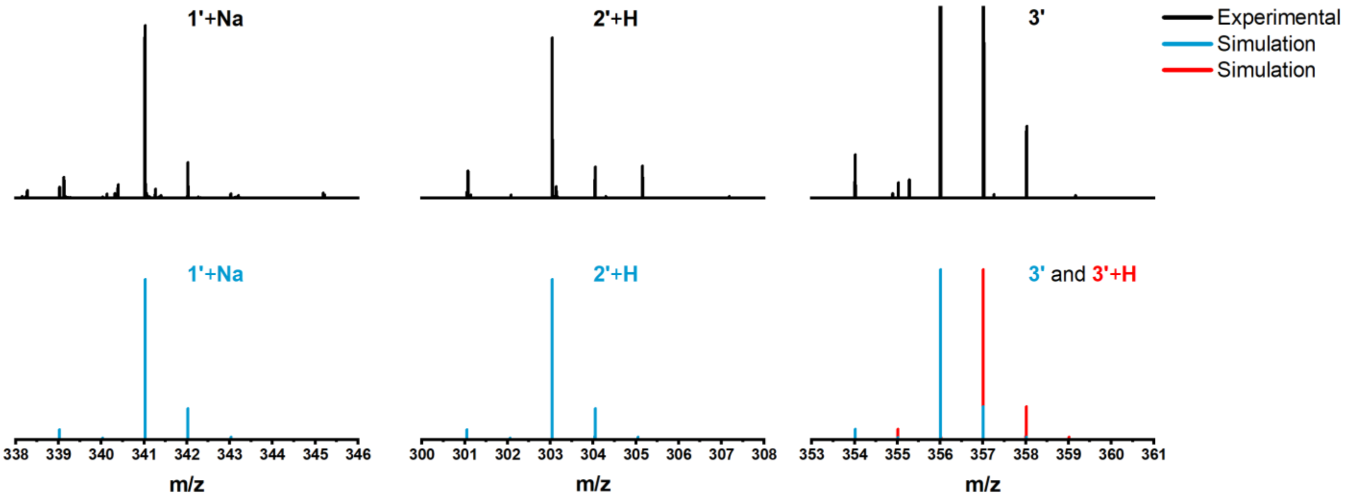

Figure S28: Experimental mass spectra of ferrocenoindenones **1' – 3'** (top, black lines) with the simulated spectra (bottom, red and blue lines).

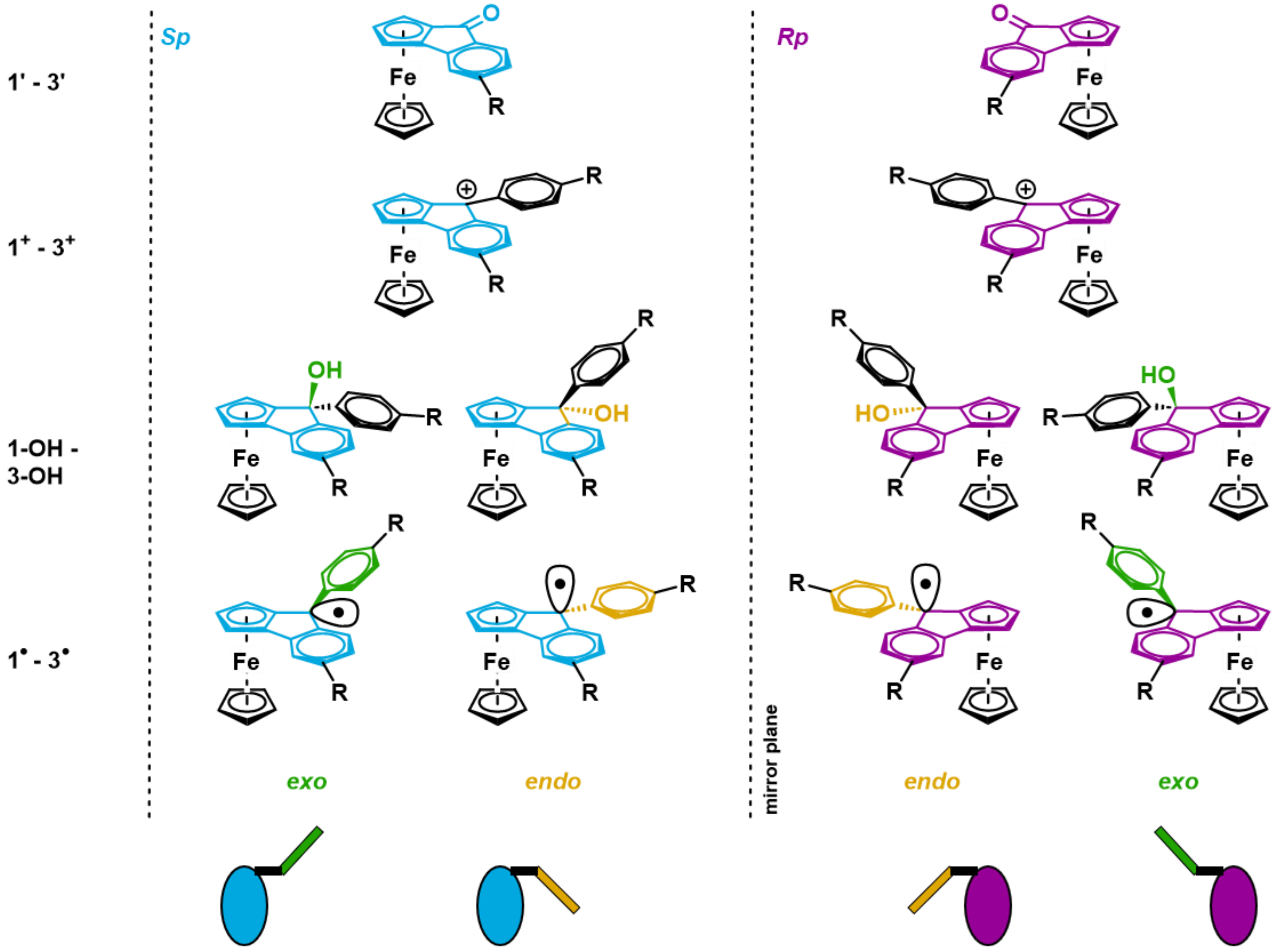

Figure S29: Target complexes of this work and the possible stereoisomers of the corresponding ketones, carbinols and radical species.

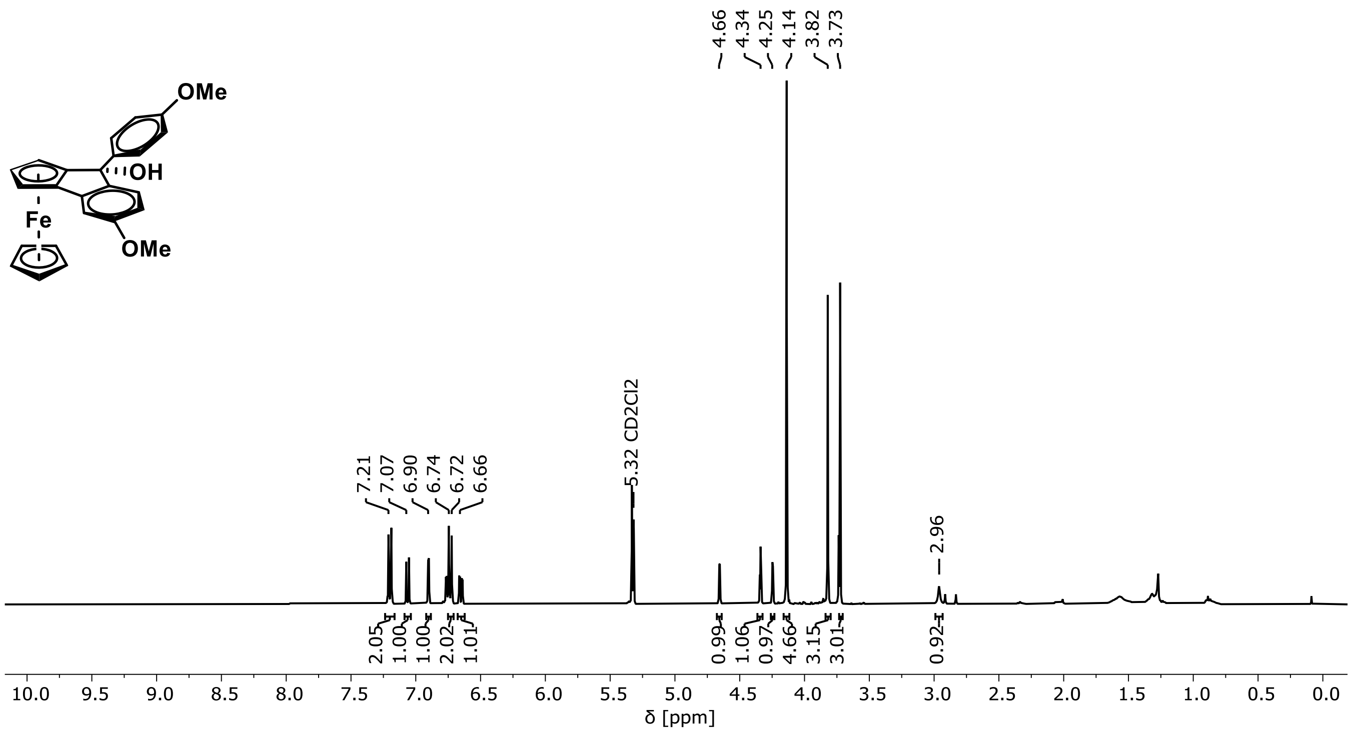

Figure S30: ^{1}H-NMR (400 MHz, CD_2Cl_2) spectrum of carbinol **1-OH**.

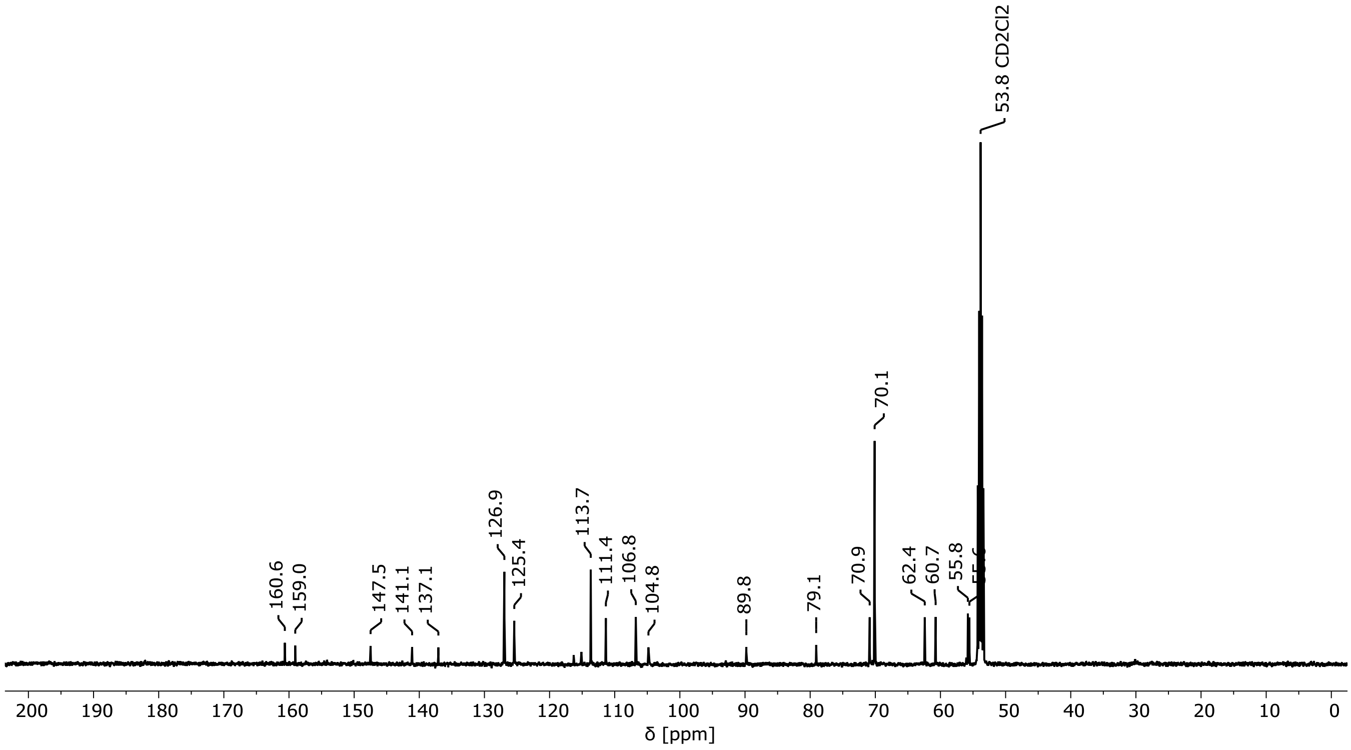

Figure S31: ^{13}C{^{1}H}-NMR (101 MHz, CD_2Cl_2) spectrum of carbinol **1-OH**.

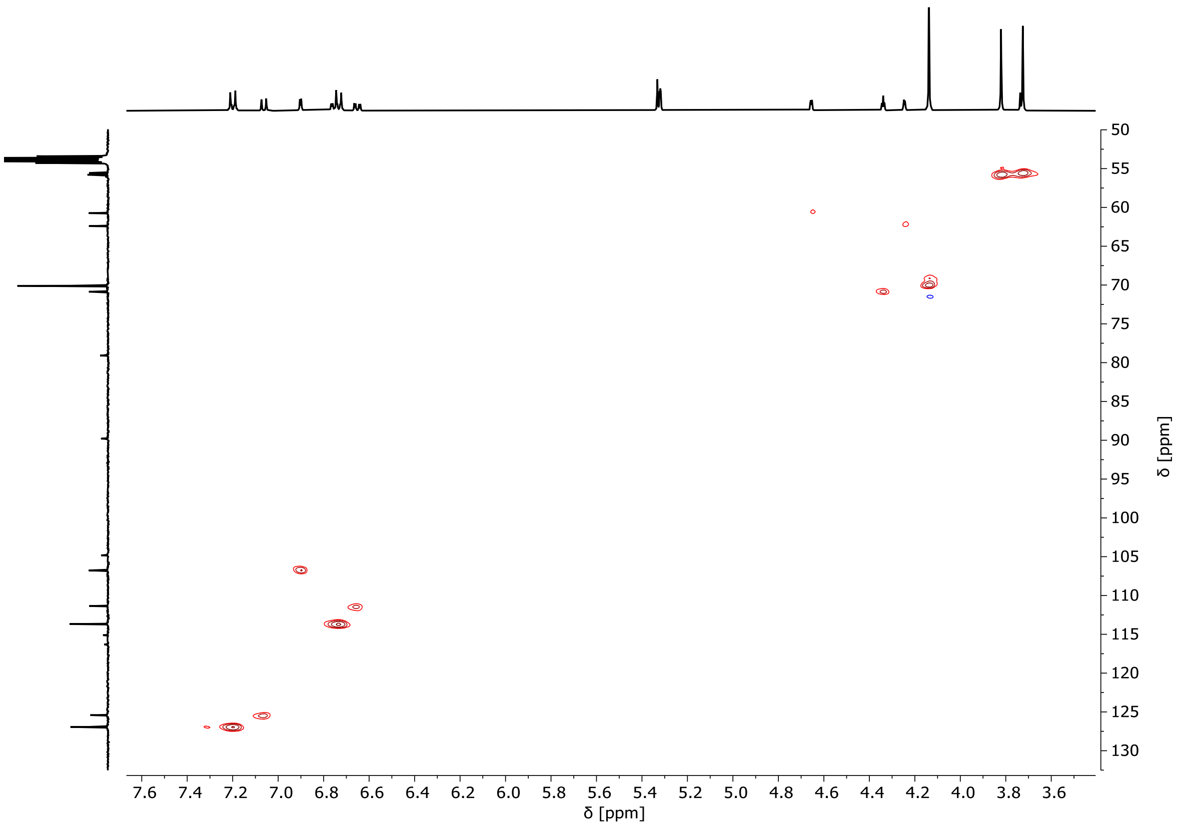

Figure S32: HSQC NMR spectrum of carbinol **1-OH**.

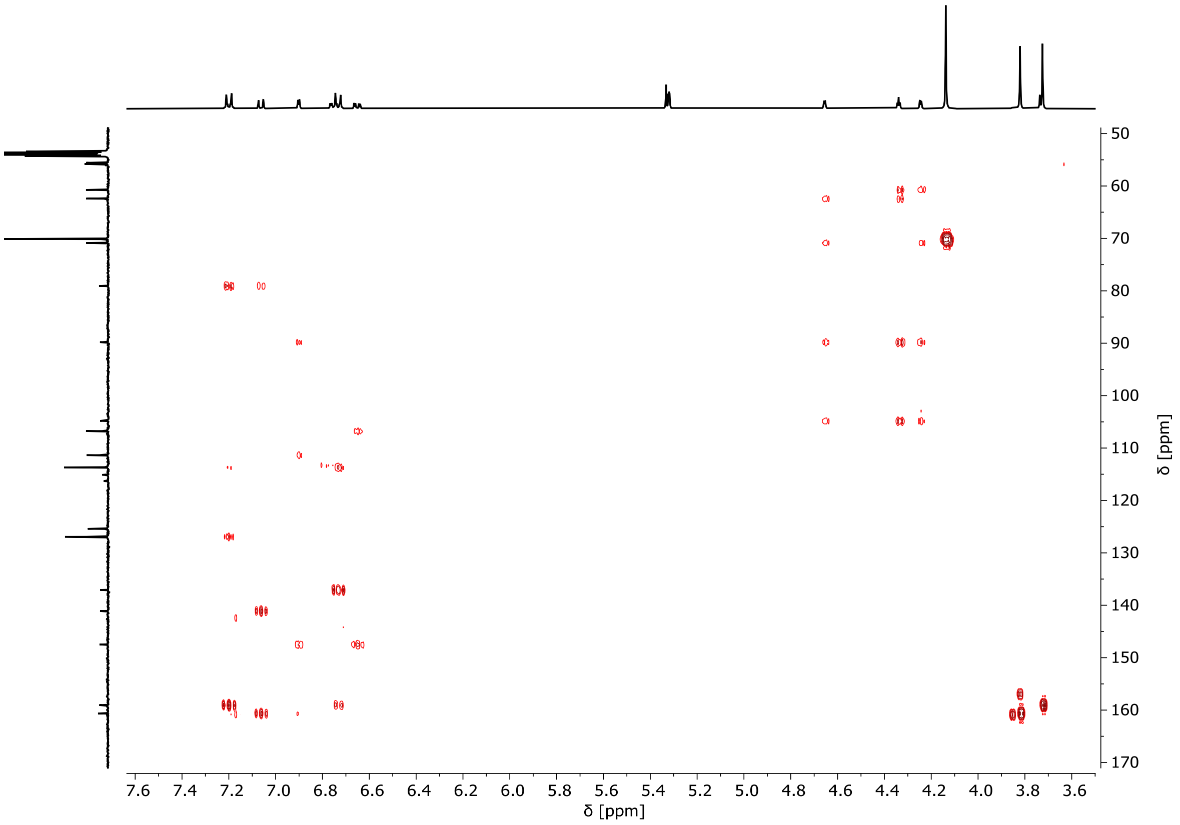

Figure S33: HMBC NMR spectrum of carbinol **1-OH**.

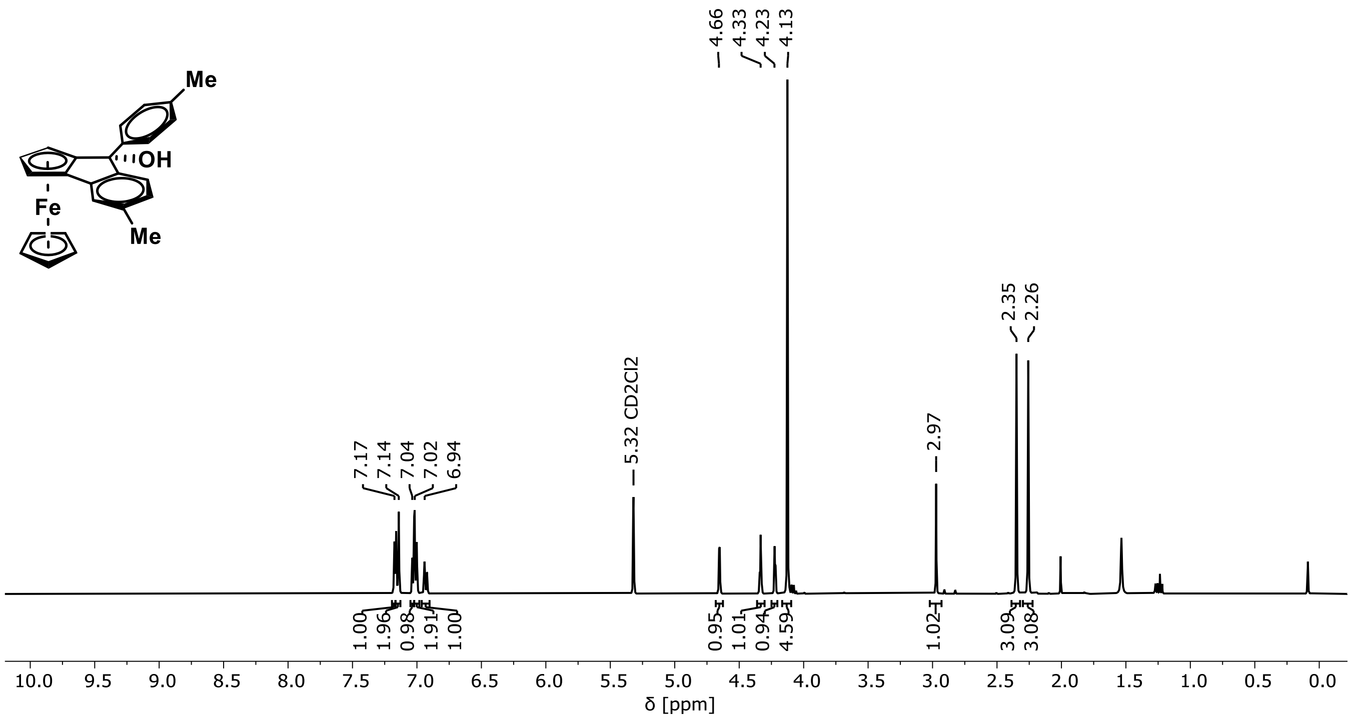

Figure S34: ^{1}H-NMR (400 MHz, CD_2Cl_2) spectrum of carbinol **2-OH**.

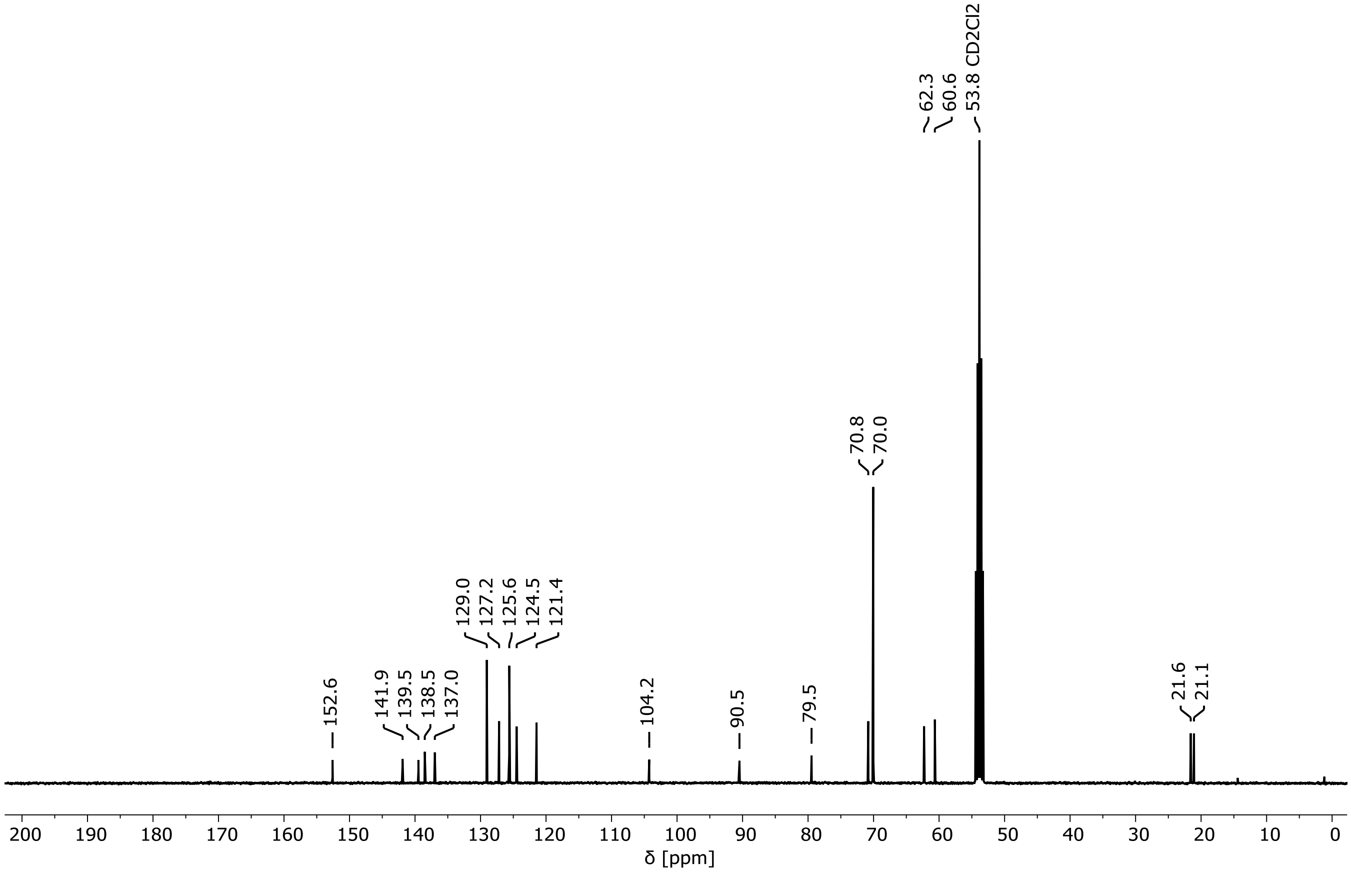

Figure S35: $^{13}C\{^{1}H\}$-NMR (101 MHz, CD_2Cl_2) spectrum of carbinol **2-OH**.

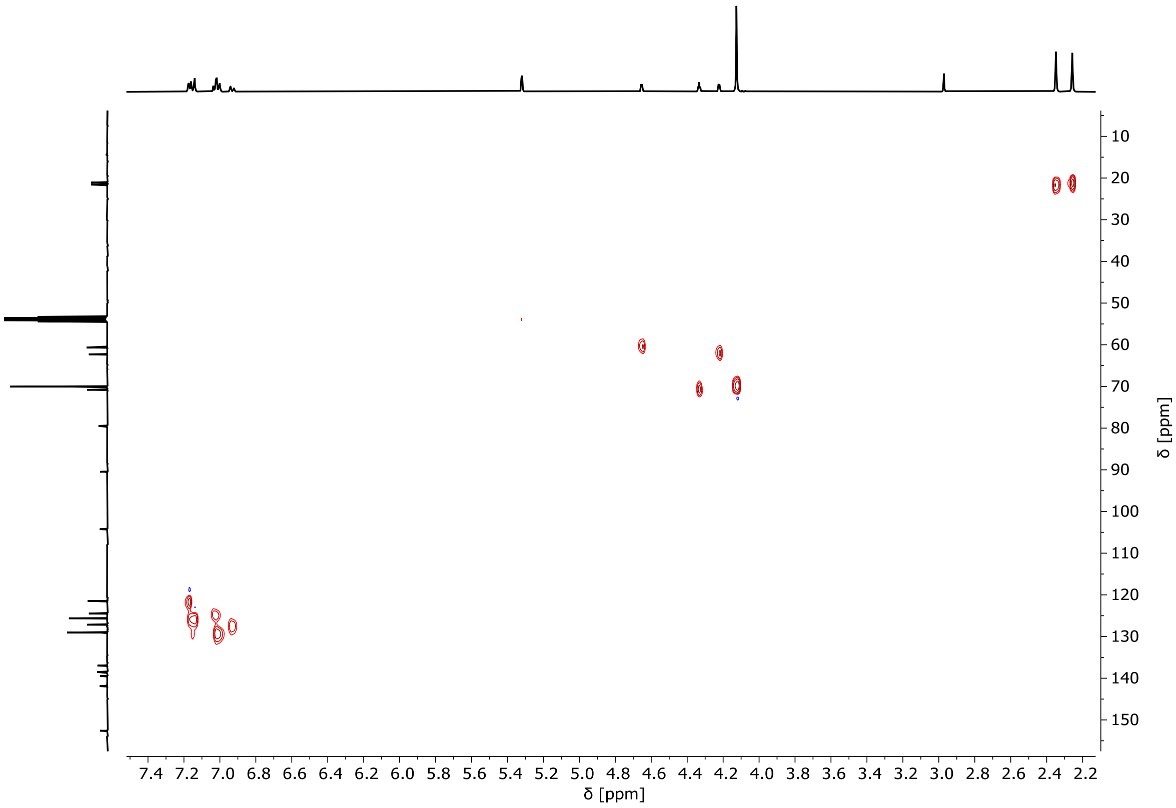

Figure S36: HSQC NMR spectrum of carbinol **2-OH**.

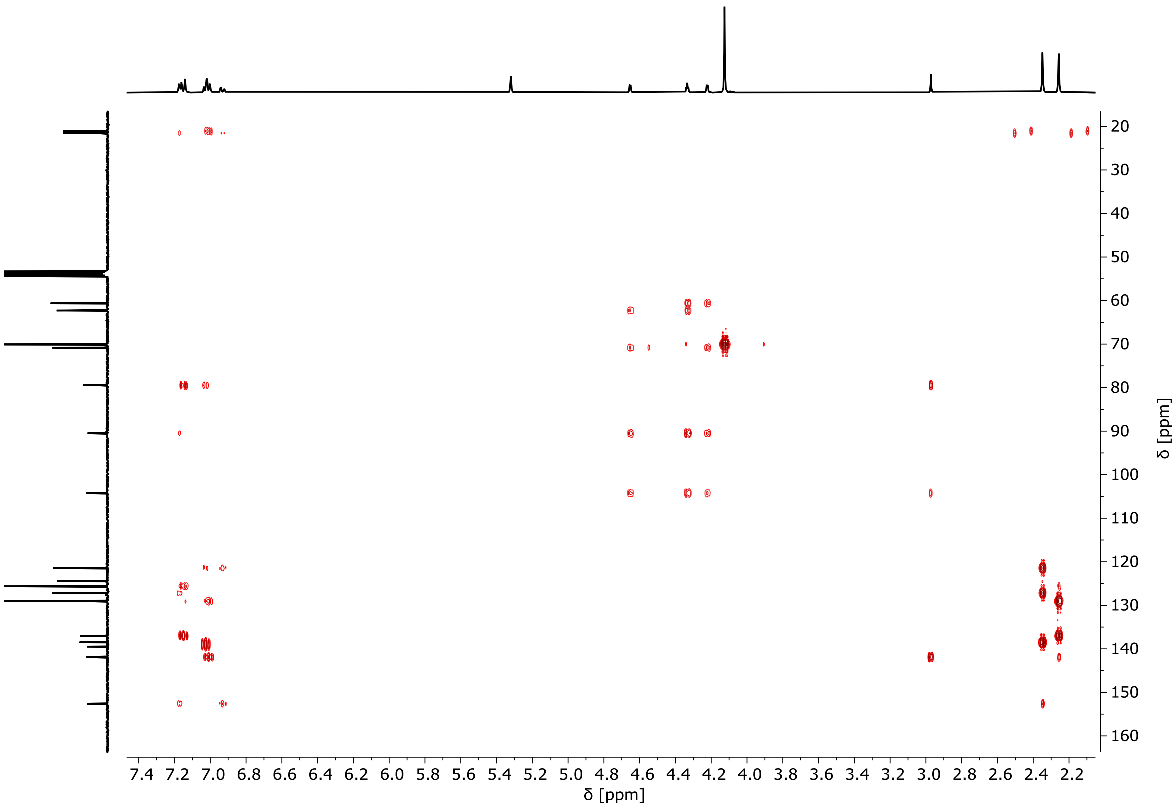

Figure S37: HMBC NMR spectrum of carbinol **2-OH**.

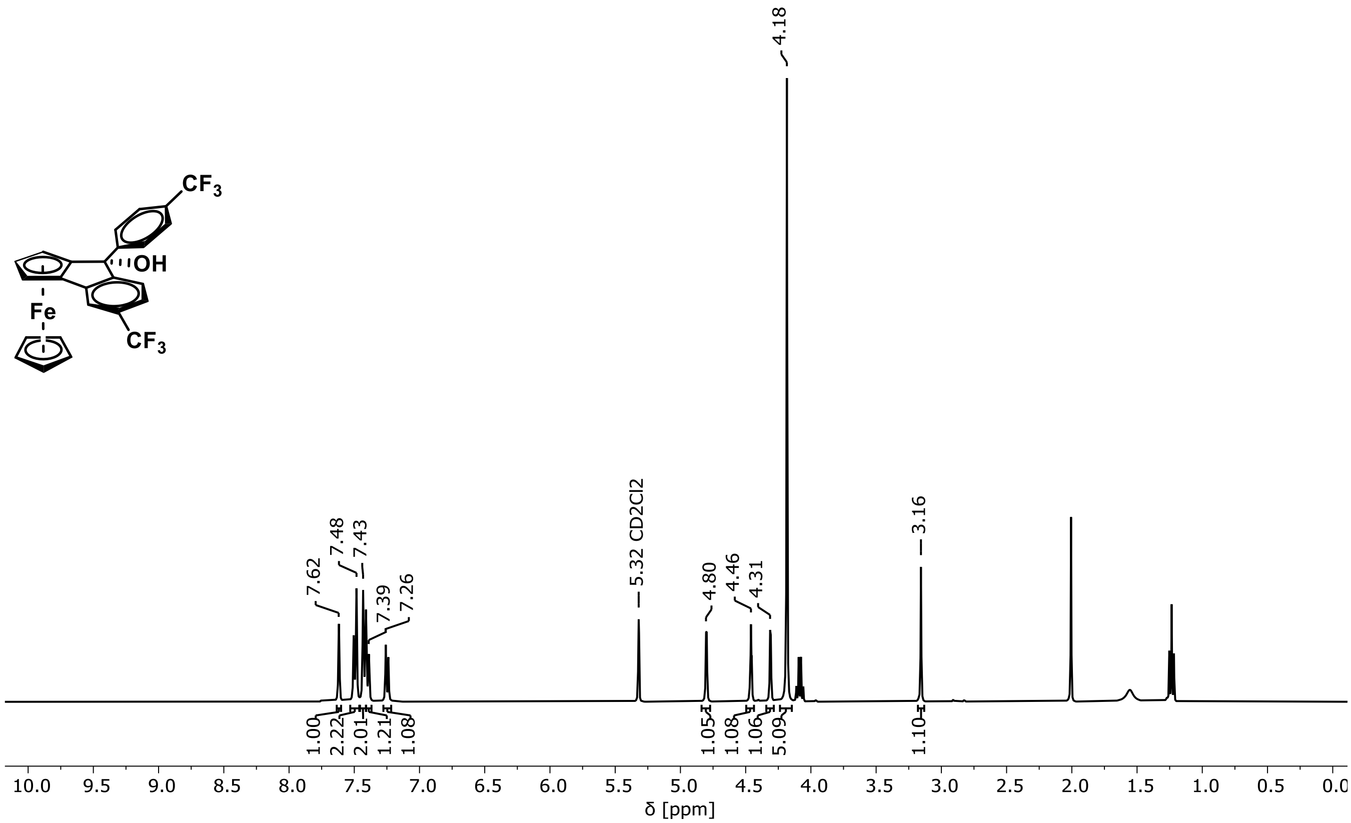

Figure S38: ^{1}H-NMR (400 MHz, CD_2Cl_2) spectrum of carbinol **3-OH**.

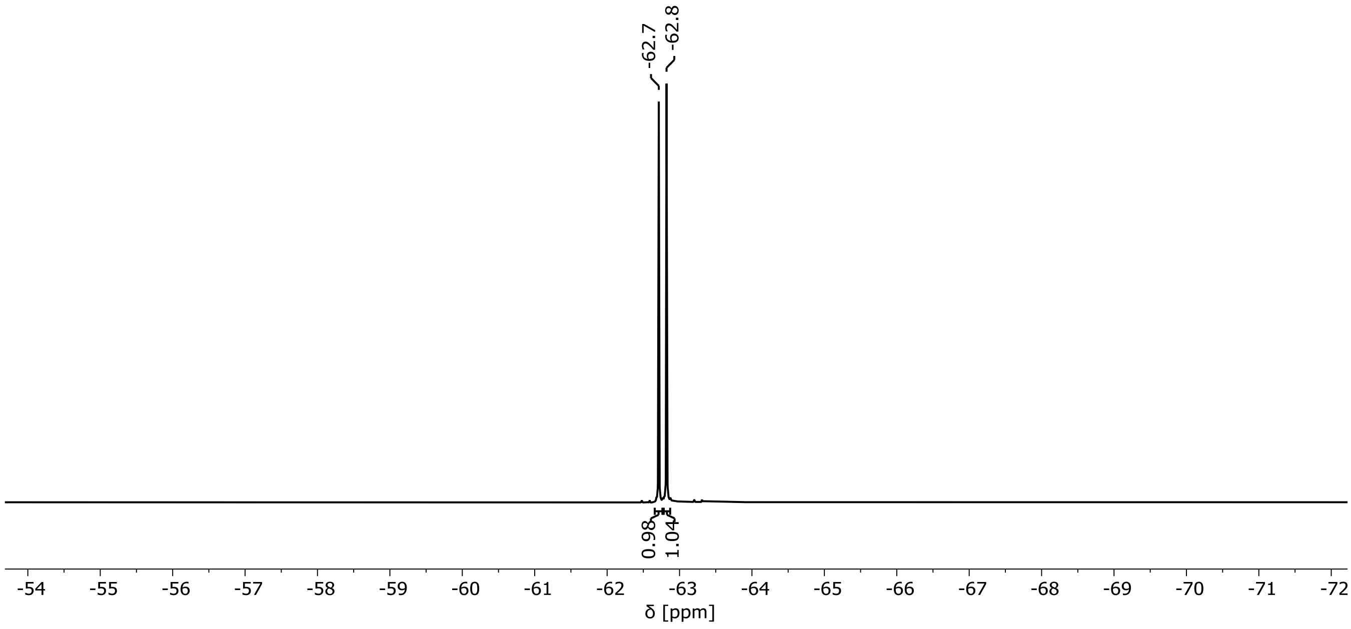

Figure S39: ^{19}F{^{1}H}-NMR (376 MHz, CD_2Cl_2) spectrum of carbinol **3-OH**.

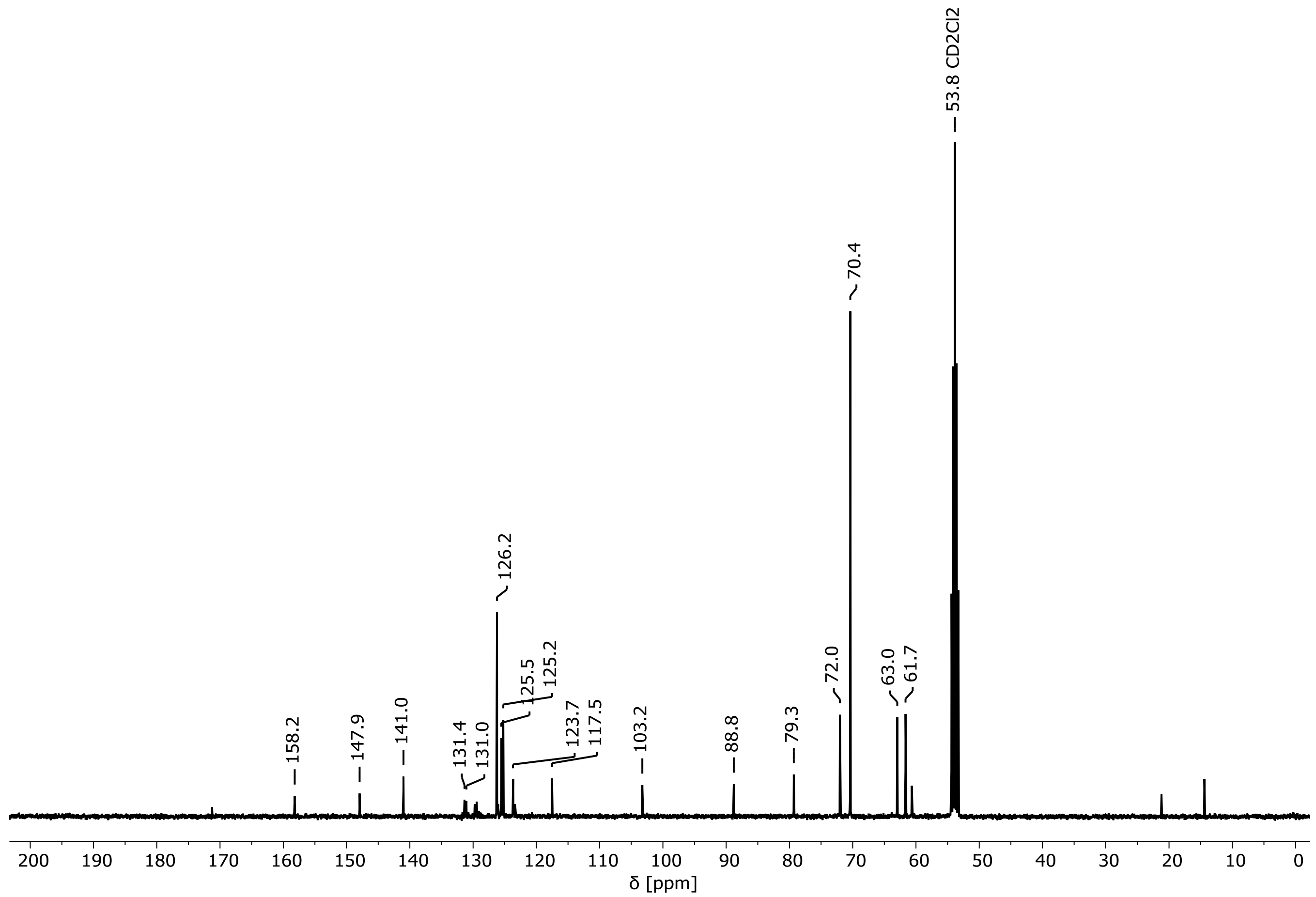

Figure S40: $^{13}C\{^{1}H\}$-NMR (101 MHz, CD_2Cl_2) spectrum of carbinol **3-OH**.

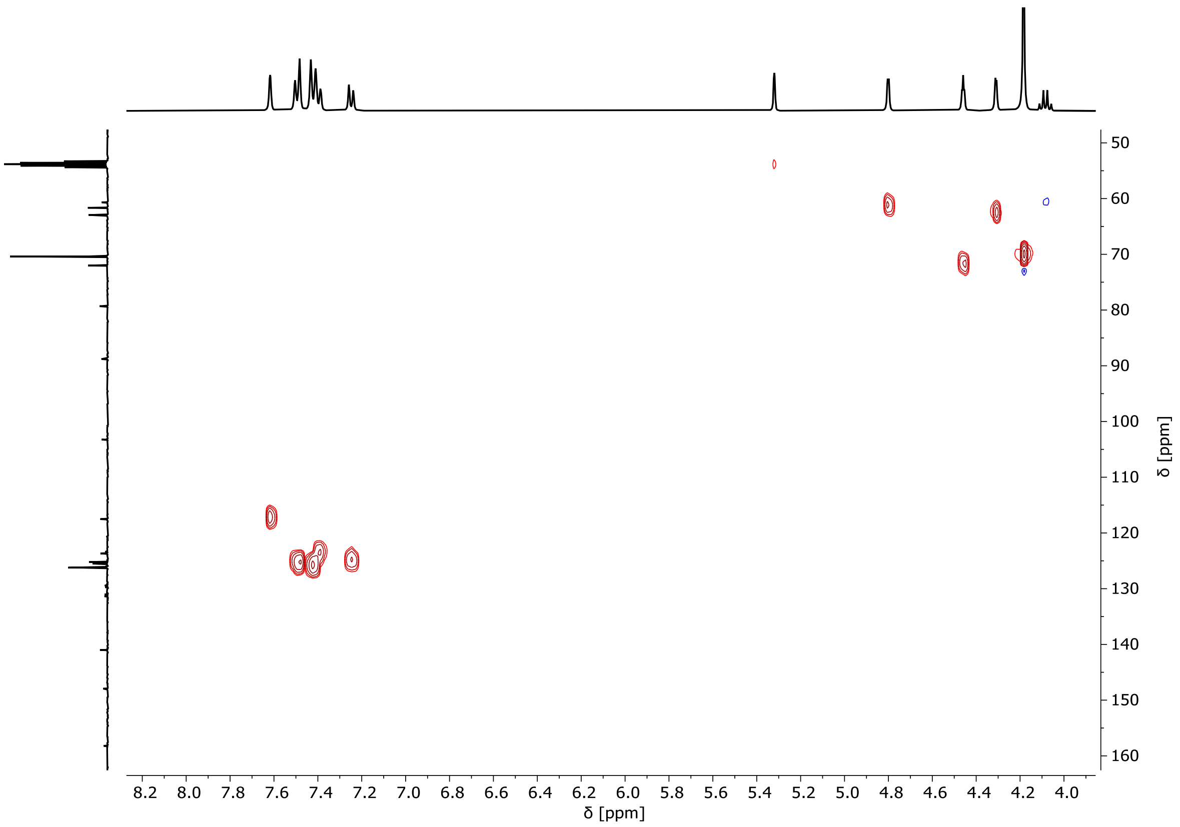

Figure S41: HSQC NMR spectrum of carbinol **3-OH**.

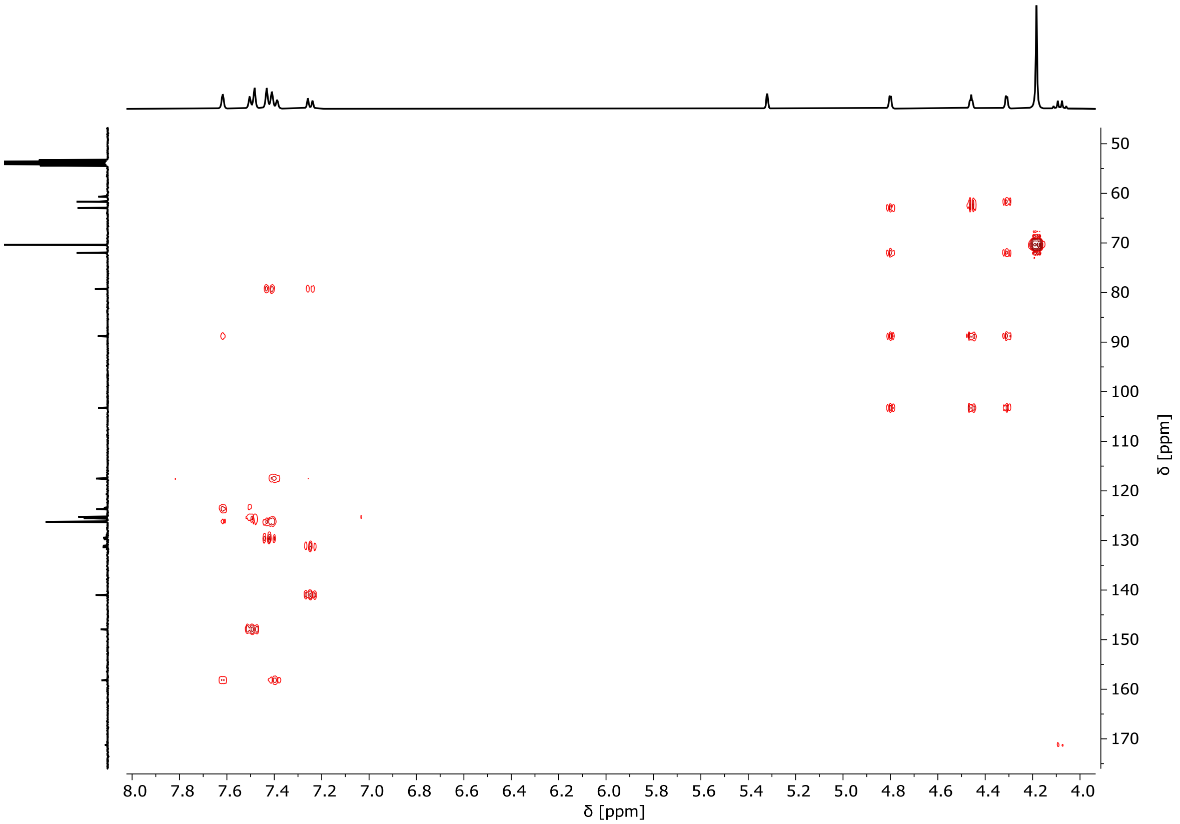

Figure S42: HMBC NMR spectrum of carbinol **3-OH**.

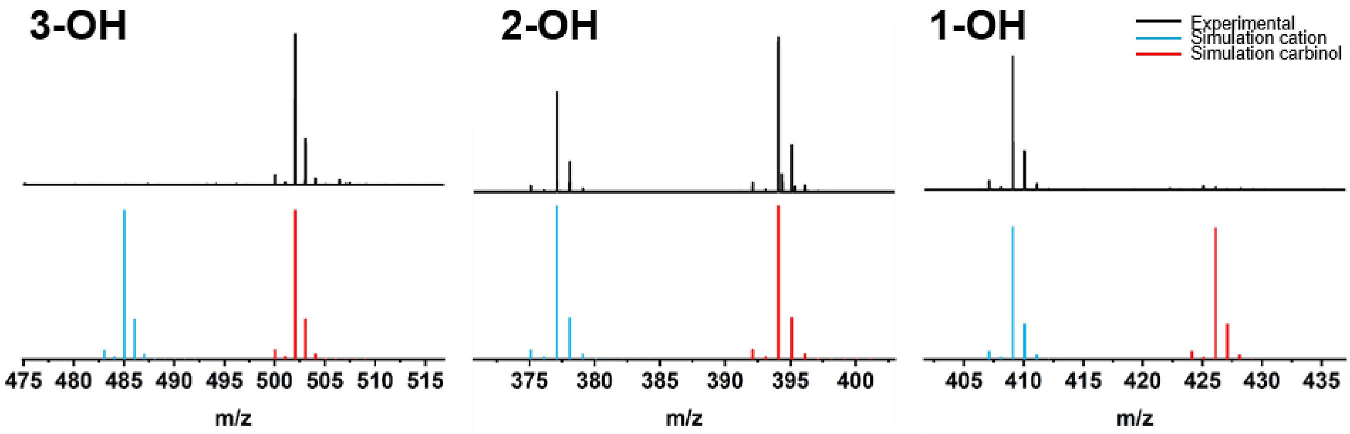

Figure S43: Experimental mass spectra of the carbinols **1-OH** – **3-OH** with the simulated distributions of the respective cations (blue), and of the carbinols (red) on the bottom.

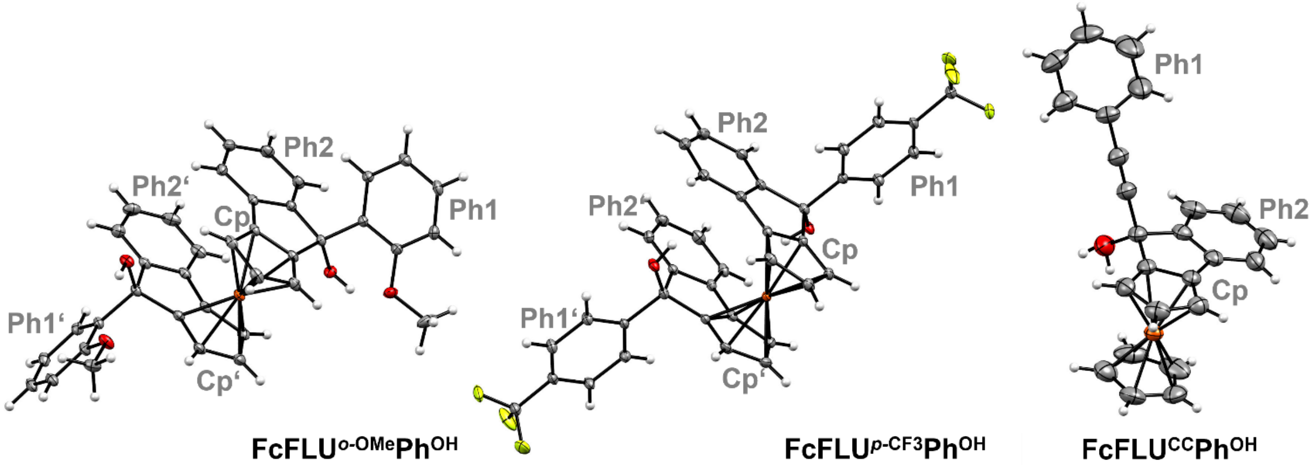

FcFLU$^{o\text{-OMe}}$Ph^{OH} **FcFLU$^{p\text{-CF3}}$Ph^{OH}** **FcFLU^{CC}Ph^{OH}**

Figure S44: Known crystal structures of other 9-phenyl carbinols derived from ferroceno[2,3]-inden-1-ones.[3,4]

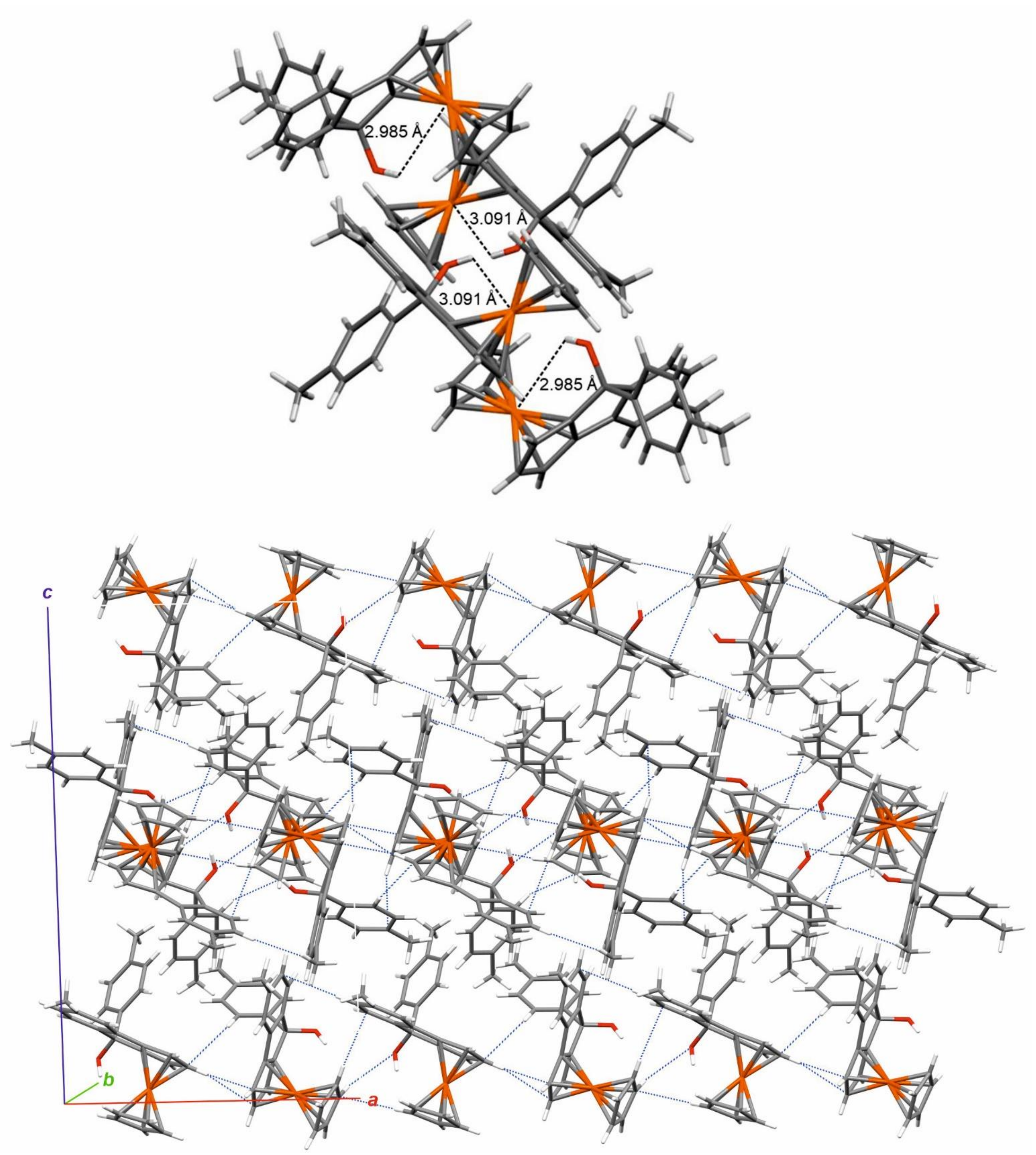

Figure S45: Top: Packing of the four molecules within the unit cell of **2-OH**. Short Fe···H-O contacts are indicated by broken lines. Bottom: Association of the tetrameric units shown in the top graphic through short CH··· π and C-H···O interactions into interdigitated chains that run parallel to the *a*-axis of the unit cell. Short contacts are indicated by blue broken lines.

Table S1: Crystal data and structure refinement for **2-OH**.

Empirical formula	$C_{50}H_{44}Fe_2O_2$
Formula weight	788.55
Temperature/K	100
Crystal system	monoclinic
Space group	$P2_1/n$
a/Å	13.6724(5)
b/Å	11.9169(4)
c/Å	22.8182(9)
β/°	90.218(3)
Volume/Å^3	3717.8(2)
Z	4
ρ_{calc}g/cm^3	1.409
μ/mm^{-1}	0.823
F(000)	1648.0
Crystal size/mm^3	0.25 × 0.217 × 0.2
Radiation	Mo Kα (λ = 0.71073)
2Θ range for data collection/°	4.868 to 54.26
Index ranges	$-17 \le h \le 17$, $-15 \le k \le 15$, $-28 \le l \le 29$
Reflections collected	22448
Independent reflections	8539 [R_{int} = 0.0712, R_{sigma} = 0.0774]
Data/restraints/parameters	8539/0/494
Goodness-of-fit on F^2	1.030
Final R indexes [$I \ge 2\sigma(I)$]	R_1 = 0.0599, wR_2 = 0.1353
Final R indexes [all data]	R_1 = 0.1314, wR_2 = 0.1792
Largest diff. peak/hole / e Å^{-3}	0.59/−0.86

Table S2: Comparison of the most pertinent bond lengths and interplanar angles of **2-OH** with those of related carbinols.[3,4]

	**2-OH**	**FcFLU$^{o\text{-MeO}}$Ph^{OH}**	**FcFLU$^{p\text{-CF3}}$Ph^{OH}**	**FcFLU^{CC}Ph^{OH}**
**space group**	monoclinic $P2_1/m$	orthorhombic $P2_12_12_1$	monoclinic $P2_1$	triclinic $P\overline{1}$
**tilt$_{Cp\text{-}Cp}$**	4.1(3)°/4.1(2)°	1.65(7)°	6.00(8)°	0.86(16)°
**conform.**	3.2(3)°/25.0(3)°	6.84(11)°	22.76(9)°	7.3(2)°
$\angle_{Ph1Ph2}$	83.5(2)°/88.7(2)°	77.81(6)°/71.90(6)°	89.96(6)°/85.87(6)°	80.72(16)°
$\angle_{CpPh1}$	84.1(2)°/81.8(2)°	80.13(6)°/72.77(6)°	88.71(7)°/88.37(7)°	79.51(16)°
$\angle_{CpPh2}$	0.6(2)°/6.9(2)°	2.76(6)°/3.32(6)°	1.42(7)°/6.25(7)°	4.97(12)°
$\angle_{Ph1\text{-}C\text{-}OH}$	107.4(3)°/106.9(3)°	111.66(9)°/110.91(9)°	106.59(9)°/106.71(9)°	n.a.
$\angle_{Ph2\text{-}C\text{-}OH}$	112.7(3)°/113.7(3)°	107.23(8)°/107.02(9)°	114.04(10)°/113.38(11)°	110.78(17)°
$\angle_{Cp\text{-}C\text{-}OH}$	114.5(3)°/113.7(3)°	116.51(9)°/116.06(9)°	114.56(10)°/116.08(10)°	115.84(19)°
**d(C-OH)**	1.427(5)/1.435(5)	1.4317(13)/1.4296(14)	1.4228(15)/1.4208(15)	1.429(2)
**d(C-Ph1)**	1.522(6)/1.513(6)	1.5332(15)/1.5355(15)	1.5354(15)/1.5281(16)	n.a.
**d(C-Ph2)**	1.535(6)/1.527(6)	1.5326(15)/1.5303(16)	1.5402(17)/1.5393(18)	1.534(3)
**d(C-Cp$^-$)**	1.506(6)/1.512(6)	1.5252(16)/1.5302(15)	1.5185(18)/1.5223(18)	1.512(3)
**d(C-C$_{flu}$)**	1.463(6)/1.472(6)	1.4637(15)/1.4623(15)	1.466(2)/1.472(2)	1.465(3)

Bond lengths d in Å.

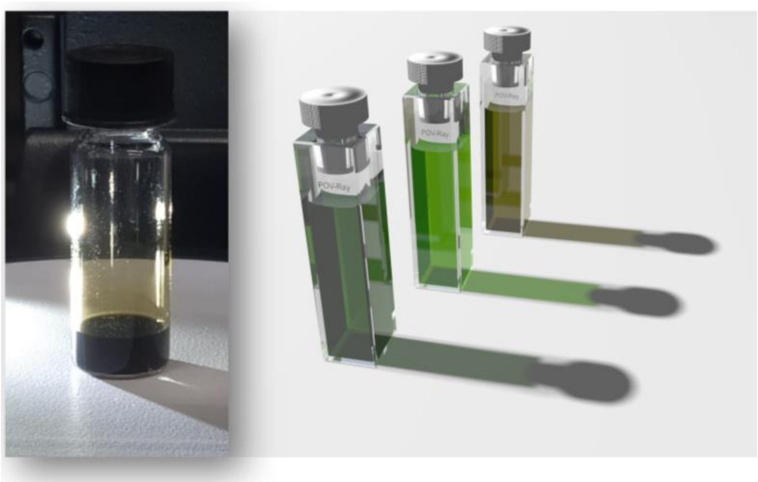

Figure S46: Left: Photo of cation **1**⁺, which stands representatively for all three cations, whose greenish colors solely differ in diluted solutions; right: color impressions generated from Vis-absorption spectra by POV-ray of the cations **1**⁺ (back), **2**⁺ (middle) and **3**⁺ (front).

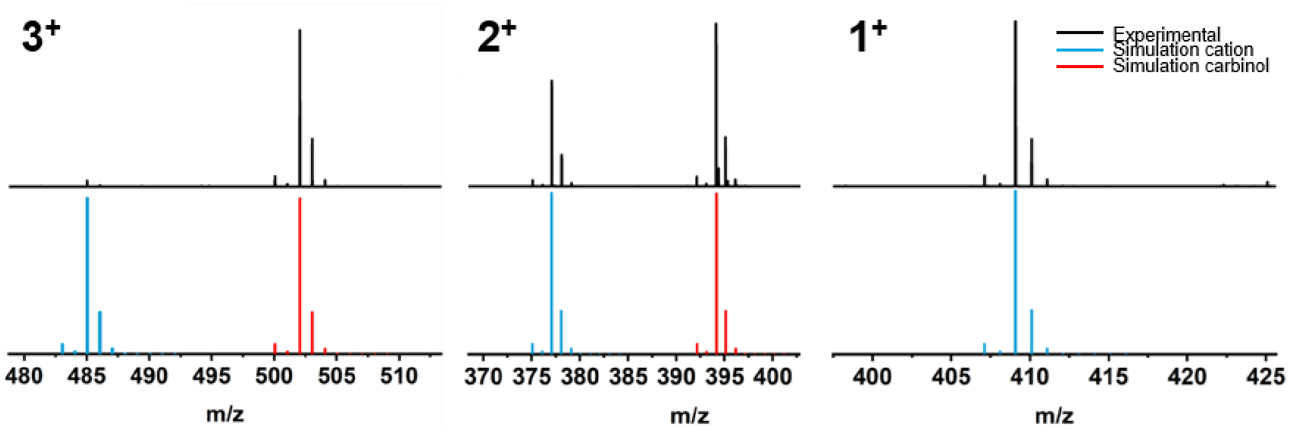

Figure S47: Experimental mass spectra of the cations **1**⁺ – **3**⁺ with the simulated distributions of the cations (blue) and the respective carbinols (red) on the bottom.

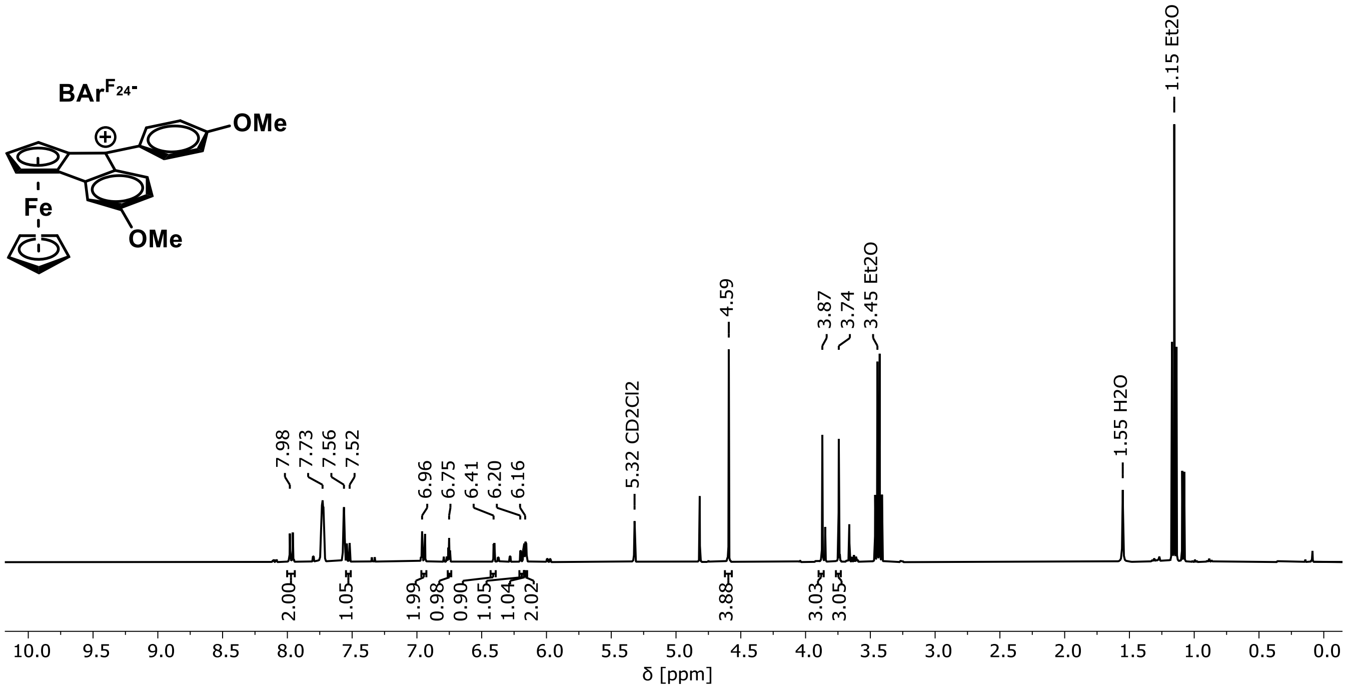

Figure S48: ^{1}H-NMR (400 MHz, CD_2Cl_2) spectrum of complex **1**$^{+}$.

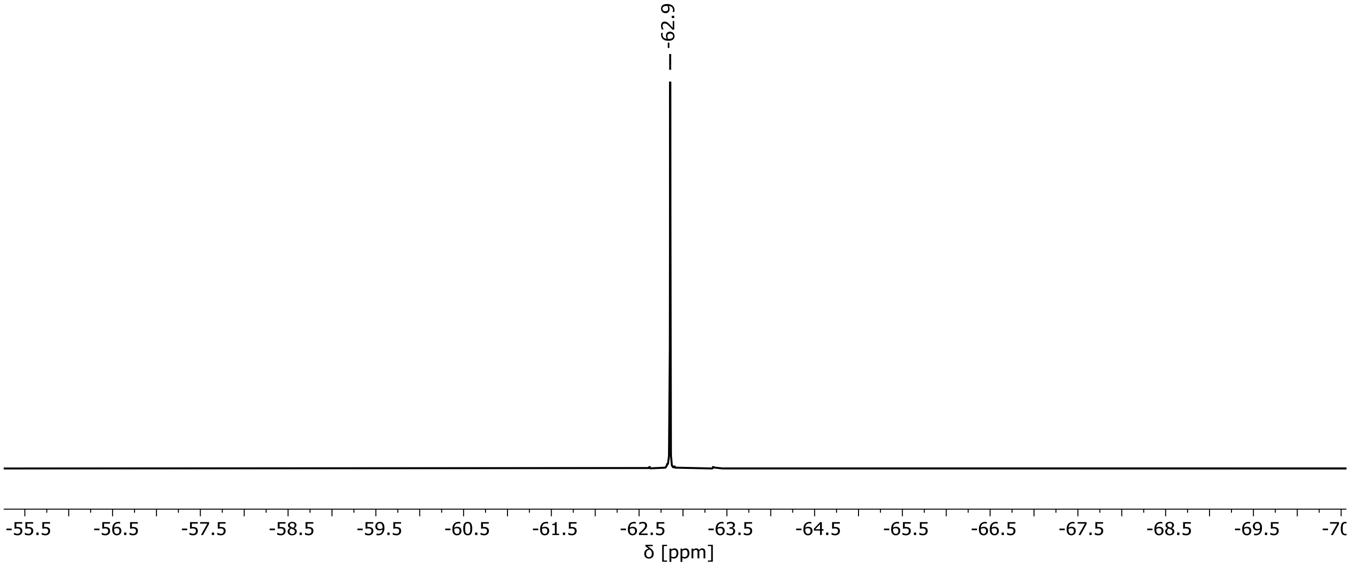

Figure S49: ^{19}F{^{1}H}-NMR (376 MHz, CD_2Cl_2) spectrum of complex **1**$^{+}$.

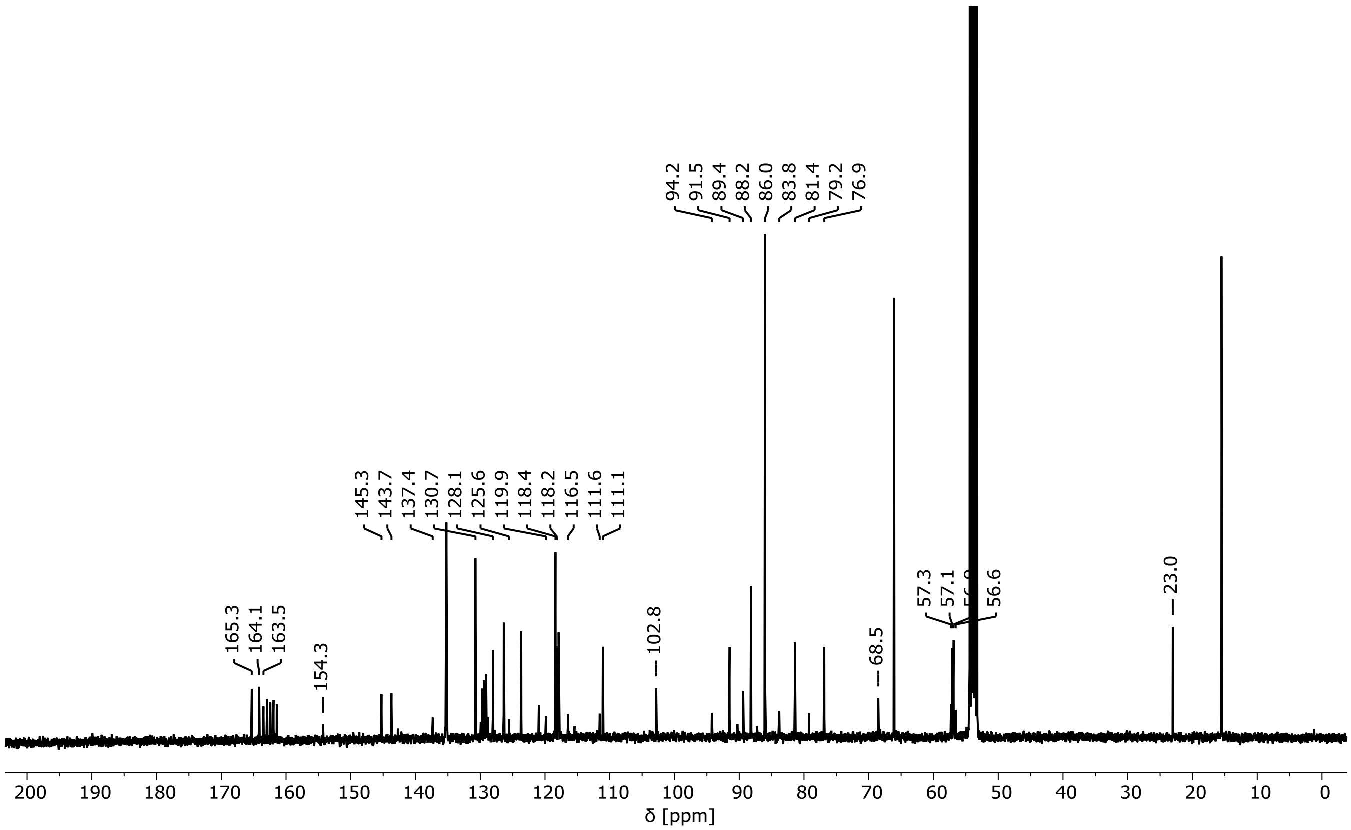

Figure S50: $^{13}C\{^{1}H\}$-NMR (101 MHz, CD_2Cl_2) spectrum of complex **1⁺**.

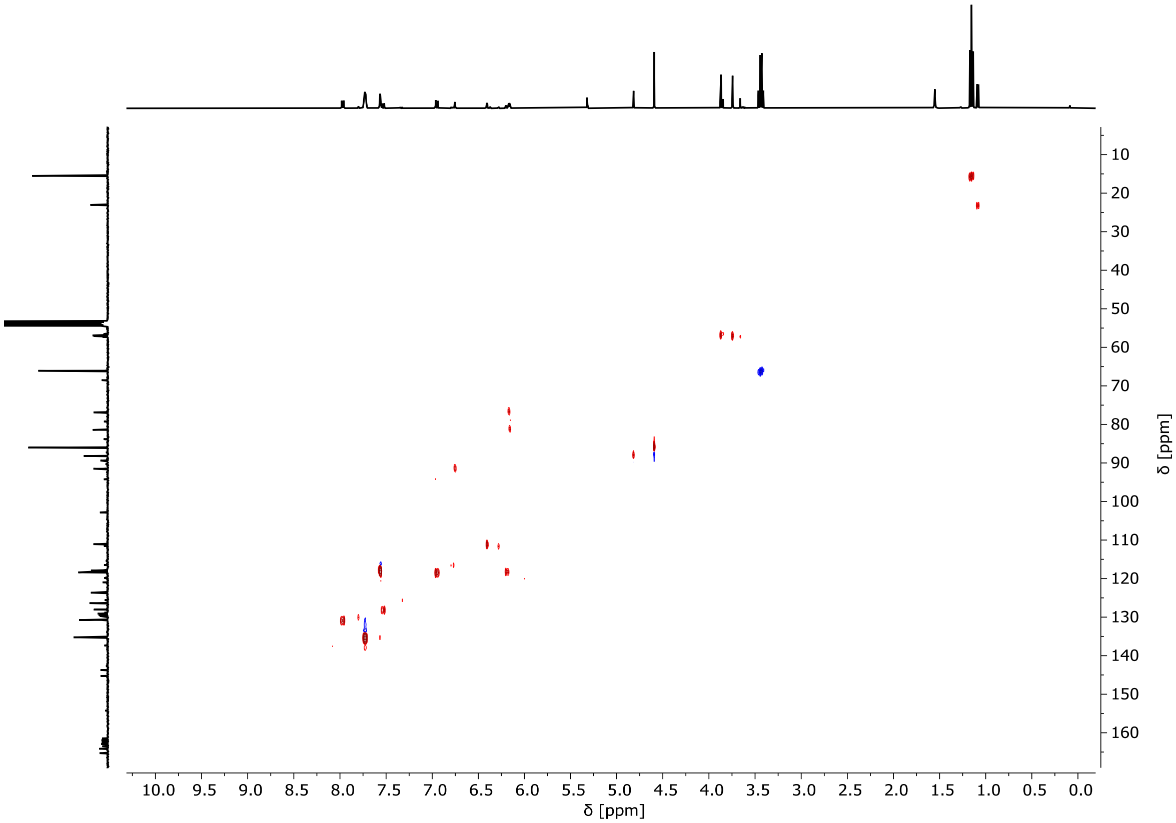

Figure S51: HSQC spectrum of complex **1⁺**.

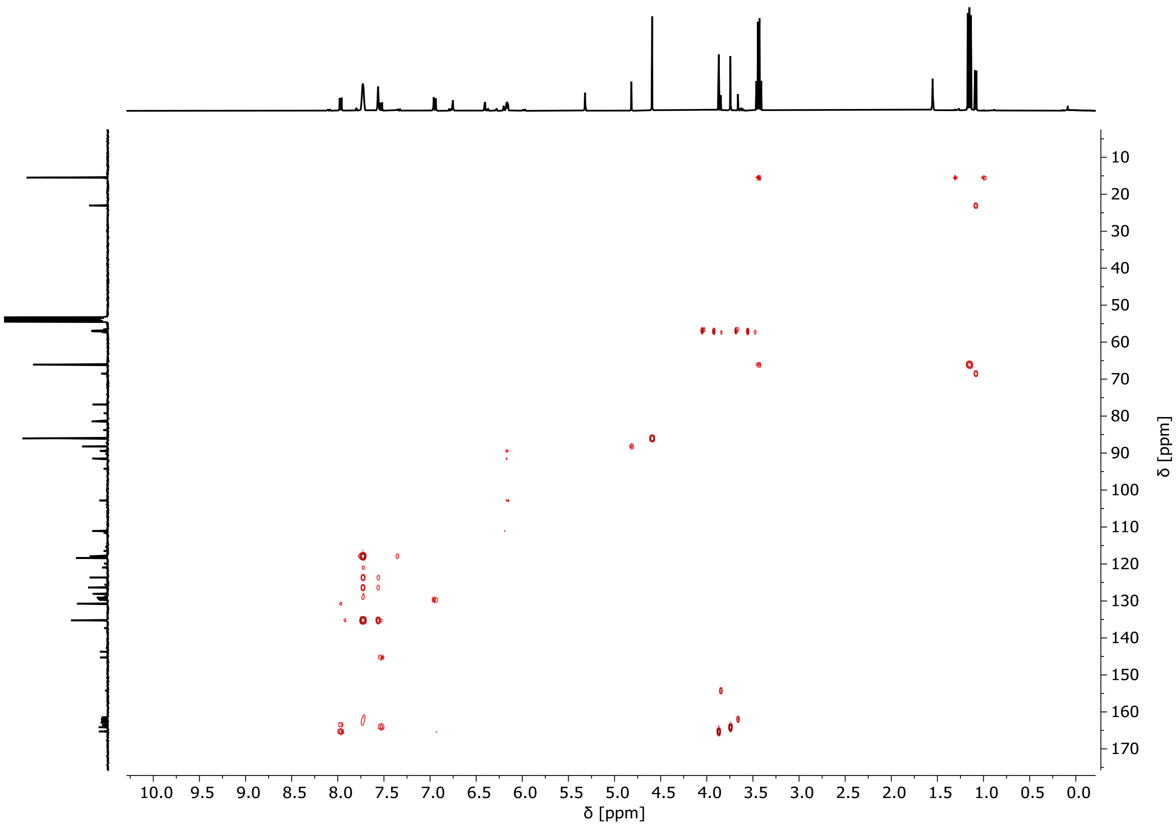

Figure S52: HMBC spectrum of complex **1⁺**.

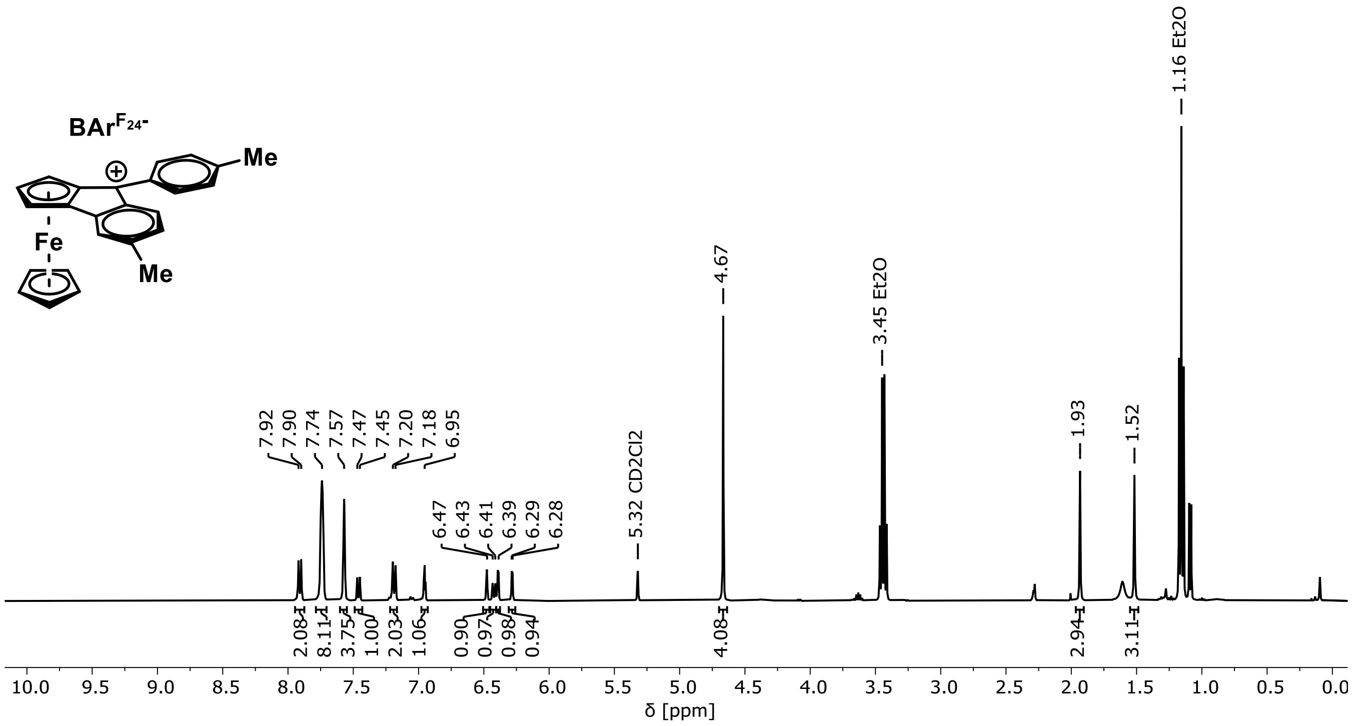

Figure S53: ^{1}H-NMR (400 MHz, CD_2Cl_2) spectrum of complex **2⁺**.

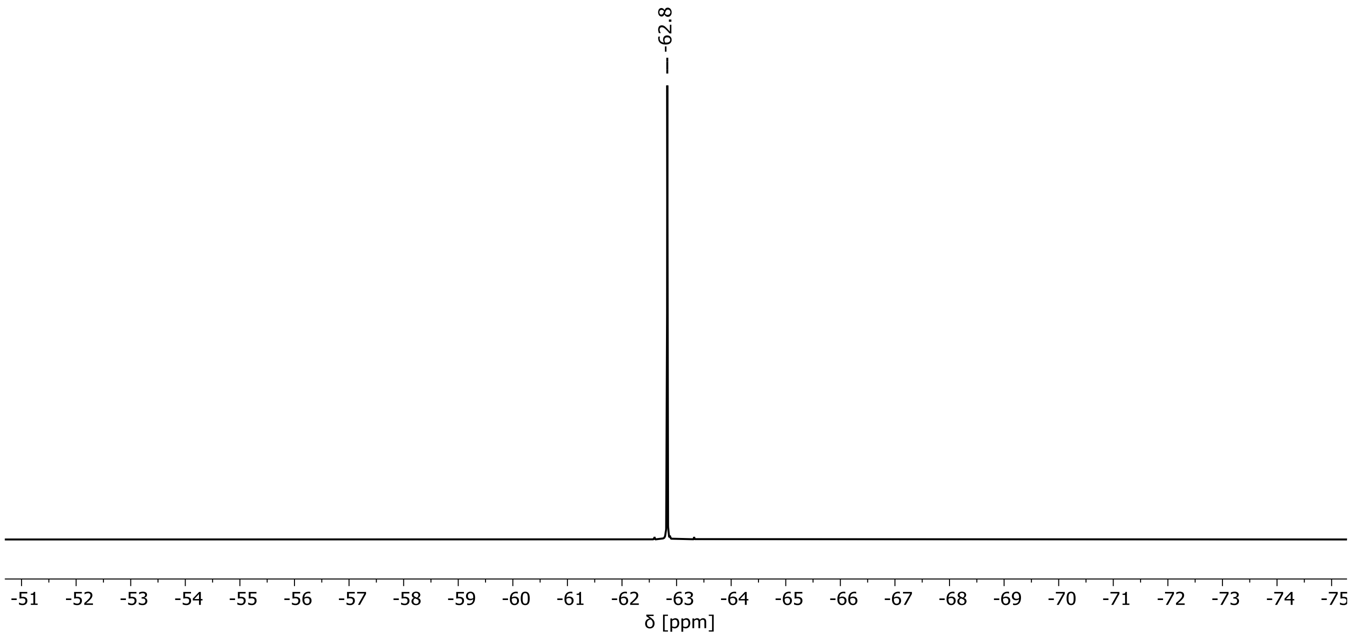

Figure S54: $^{19}F\{^1H\}$-NMR (376 MHz, CD_2Cl_2) spectrum of complex **2**$^+$.

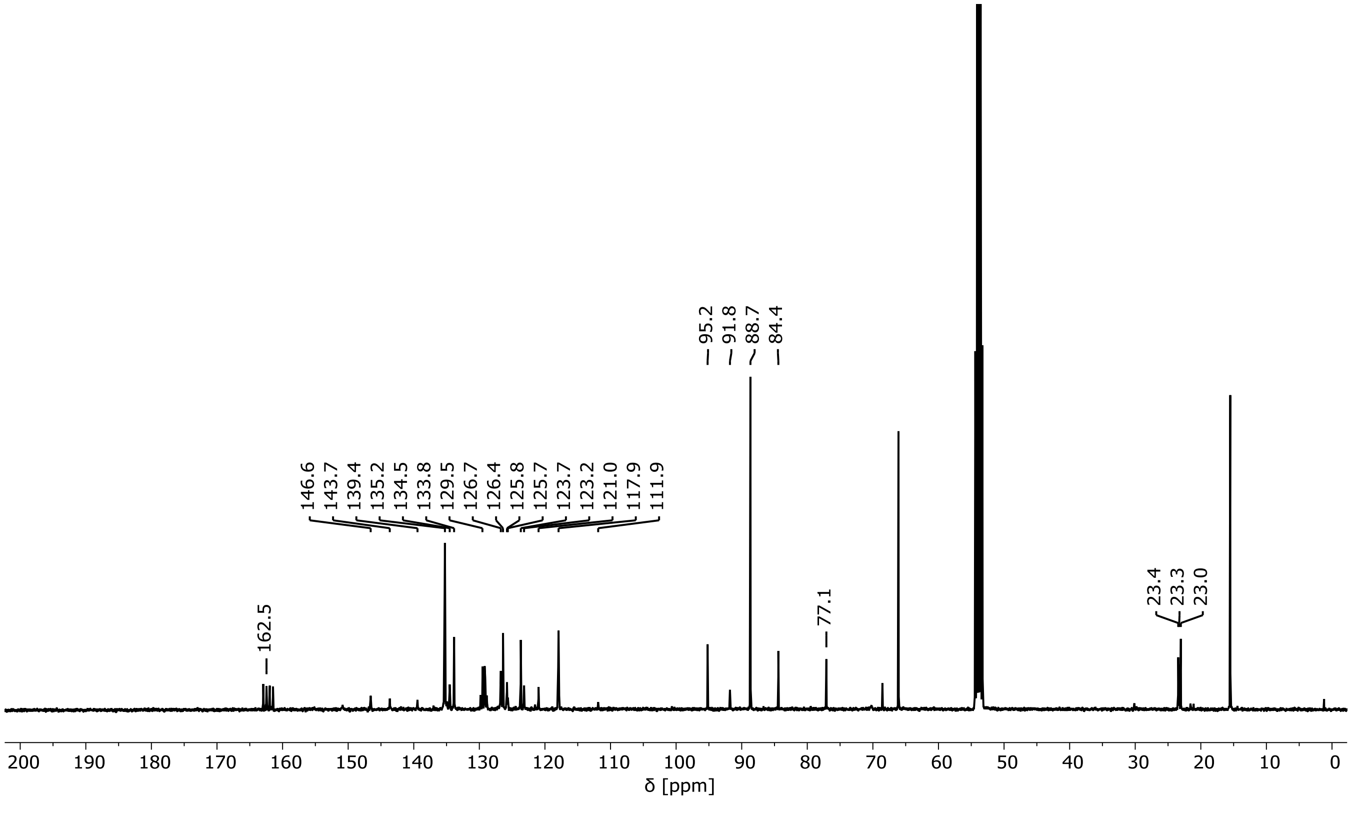

Figure S55: $^{13}C\{^1H\}$-NMR (101 MHz, CD_2Cl_2) spectrum of complex **2**$^+$.

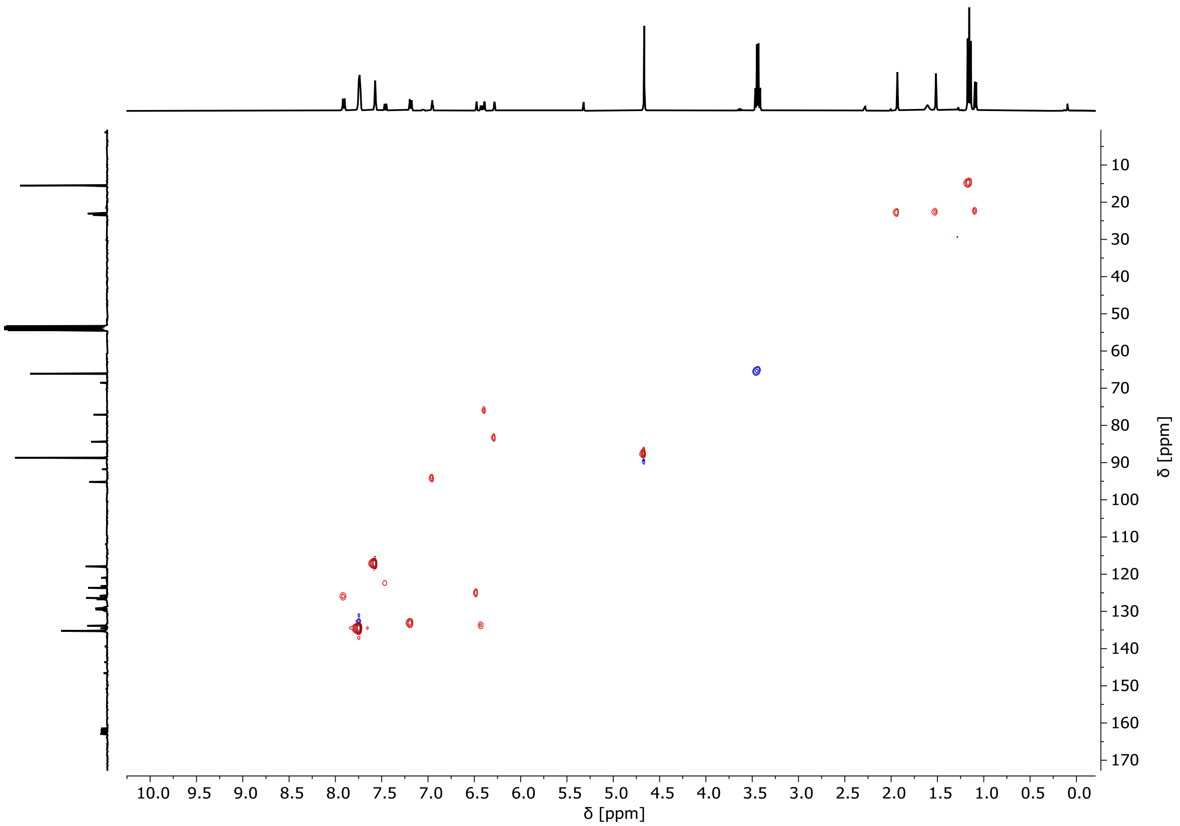

Figure S56: HSQC spectrum of complex **2**$^+$.

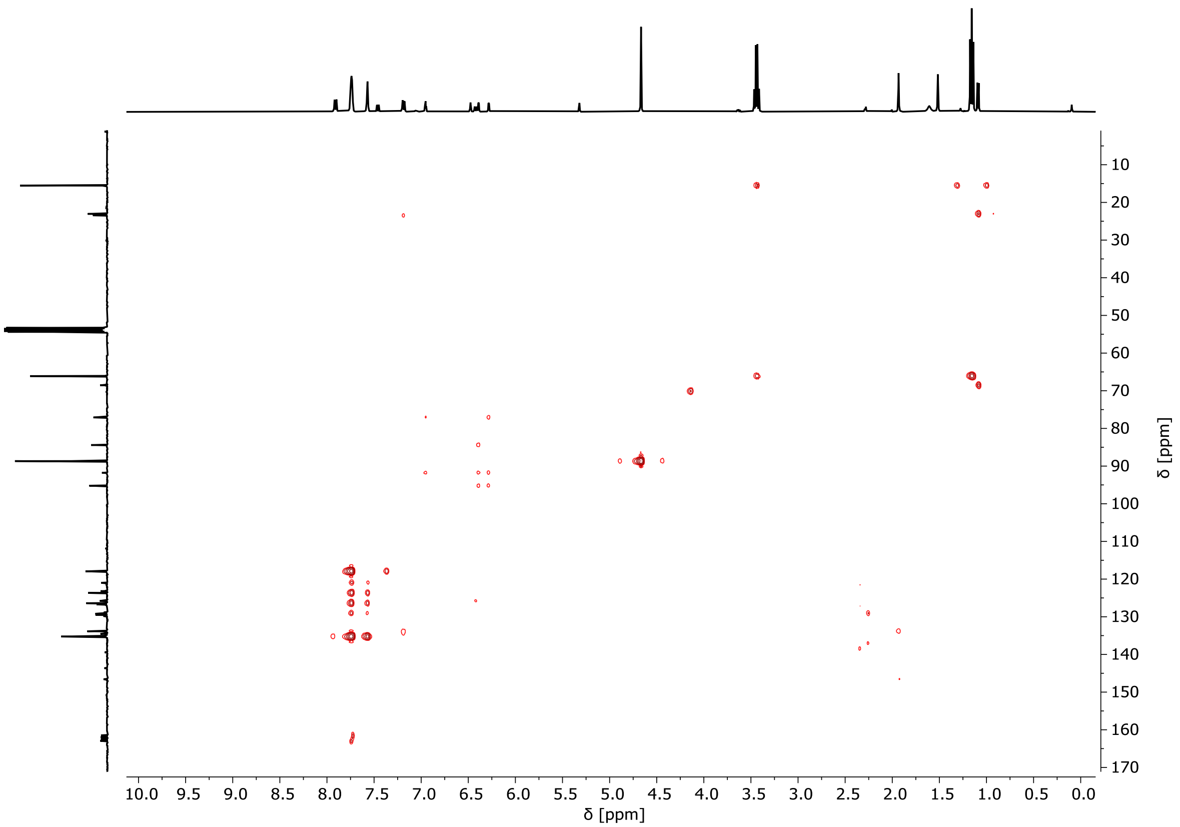

Figure S57: HMBC spectrum of complex **2**$^+$.

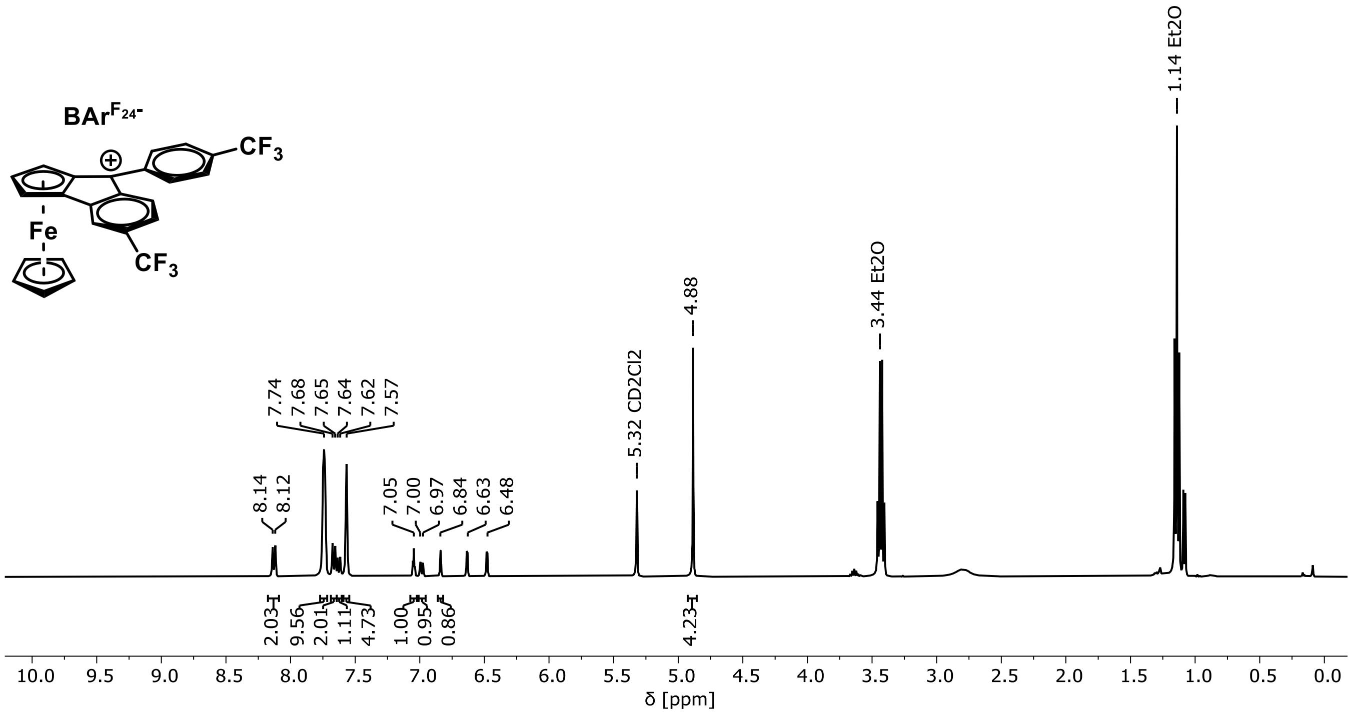

Figure S58: ^{1}H-NMR (400 MHz, CD_2Cl_2) spectrum of complex **3⁺**.

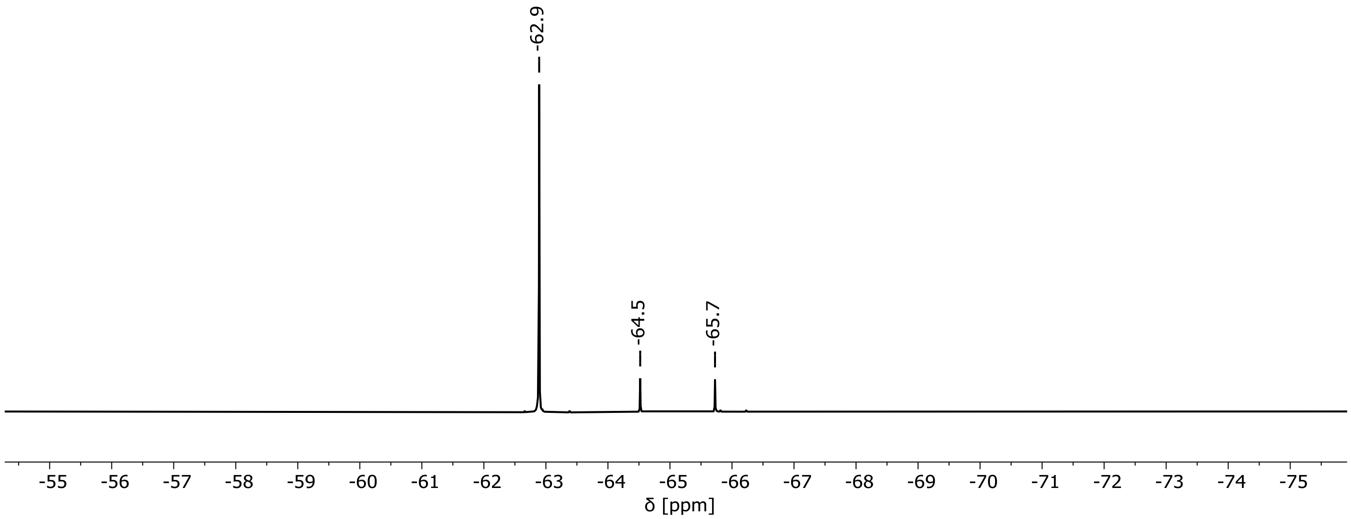

Figure S59: $^{19}F\{^{1}H\}$-NMR (376 MHz, CD_2Cl_2) spectrum of complex **3⁺**.

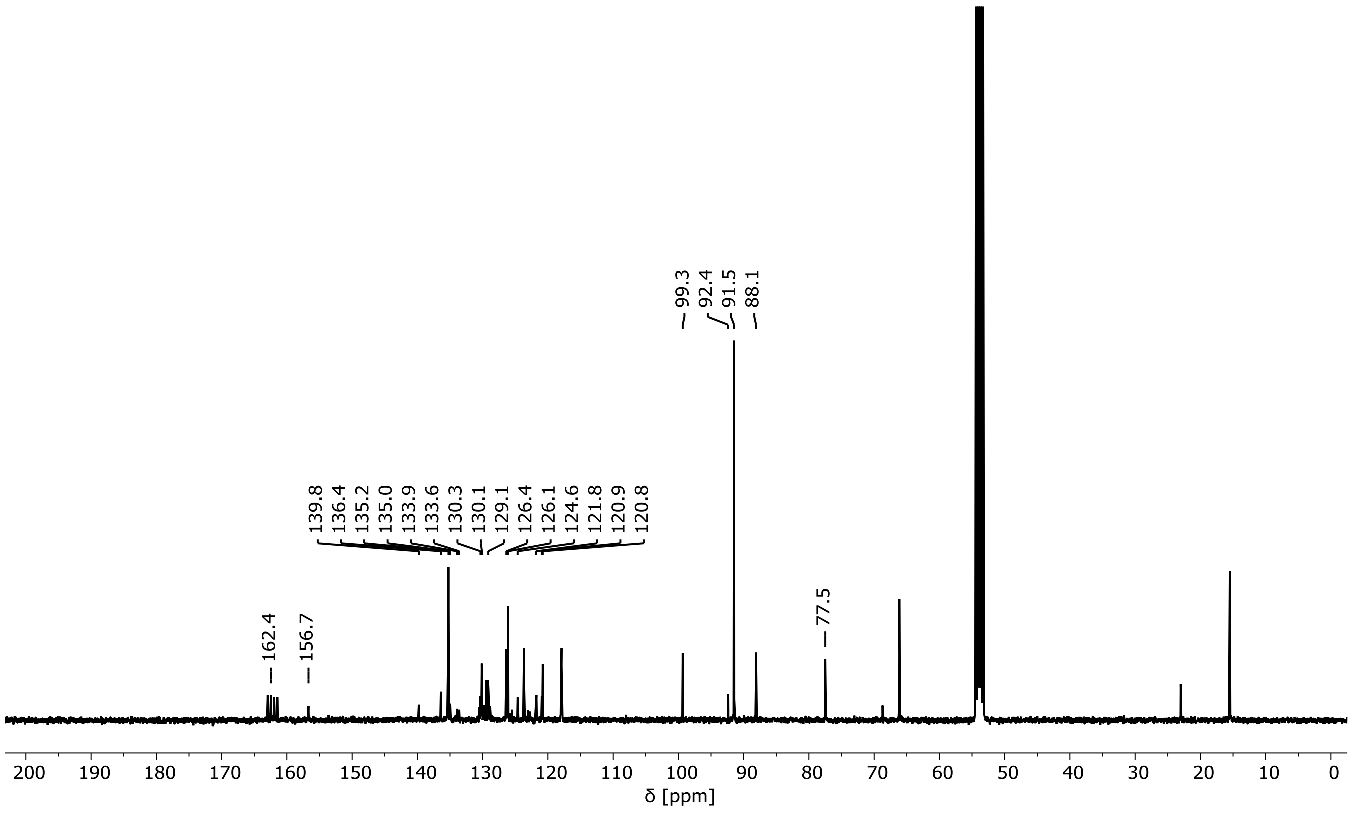

Figure S60: $^{13}C\{^{1}H\}$-NMR (101 MHz, CD_2Cl_2) spectrum of complex **3⁺**.

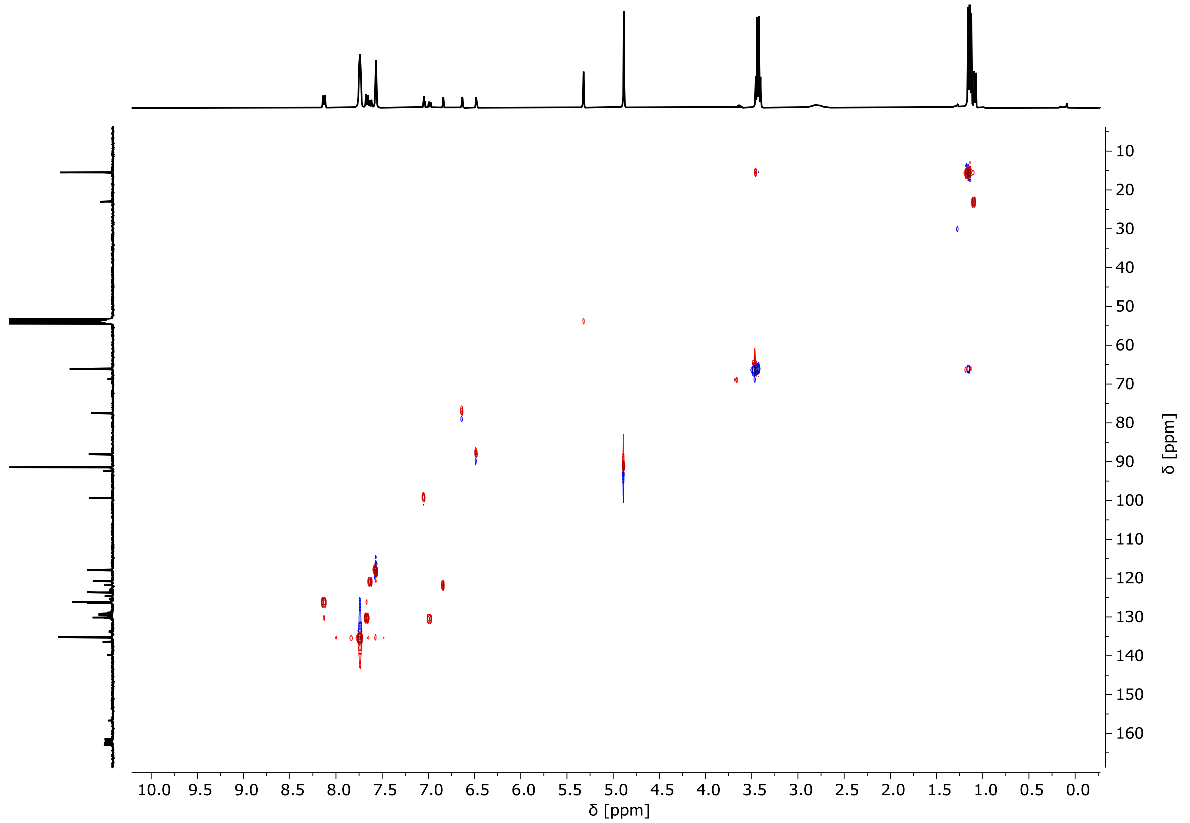

Figure S61: HSQC spectrum of complex **3⁺**.

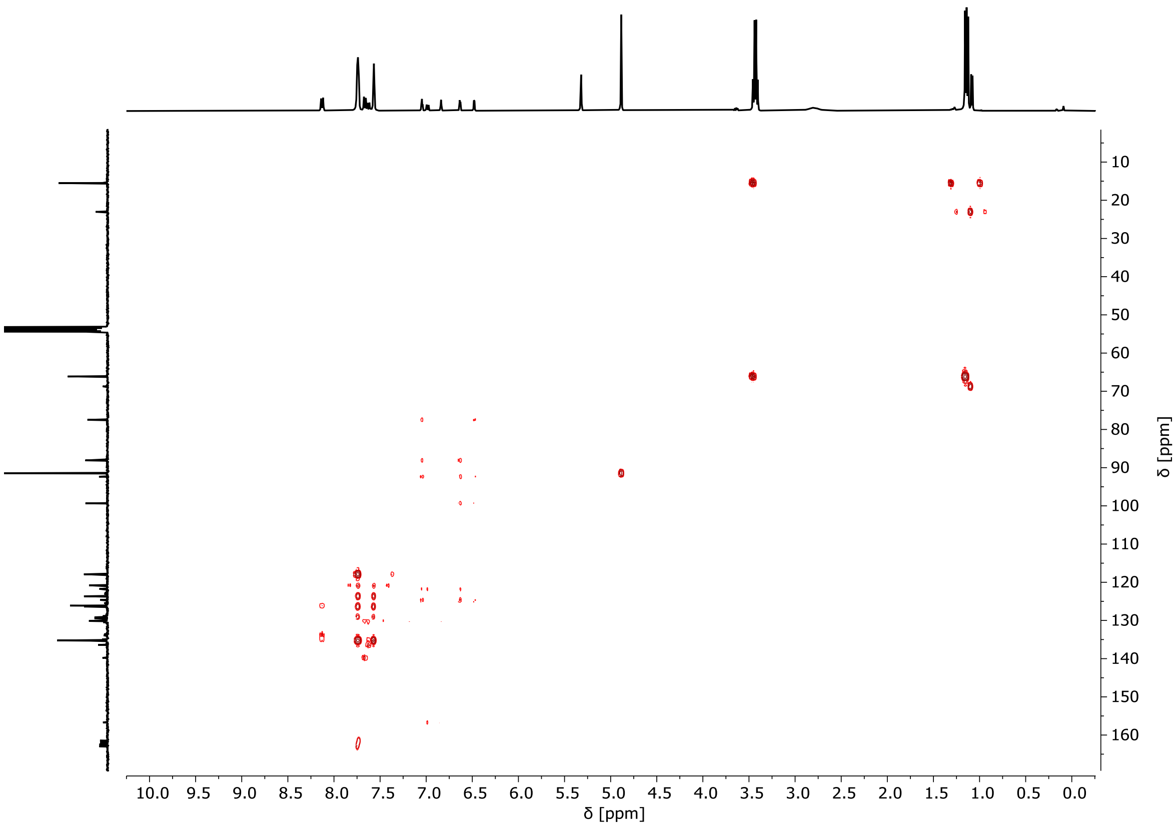

Figure S62: HMBC spectrum of complex **3**$^{+}$.

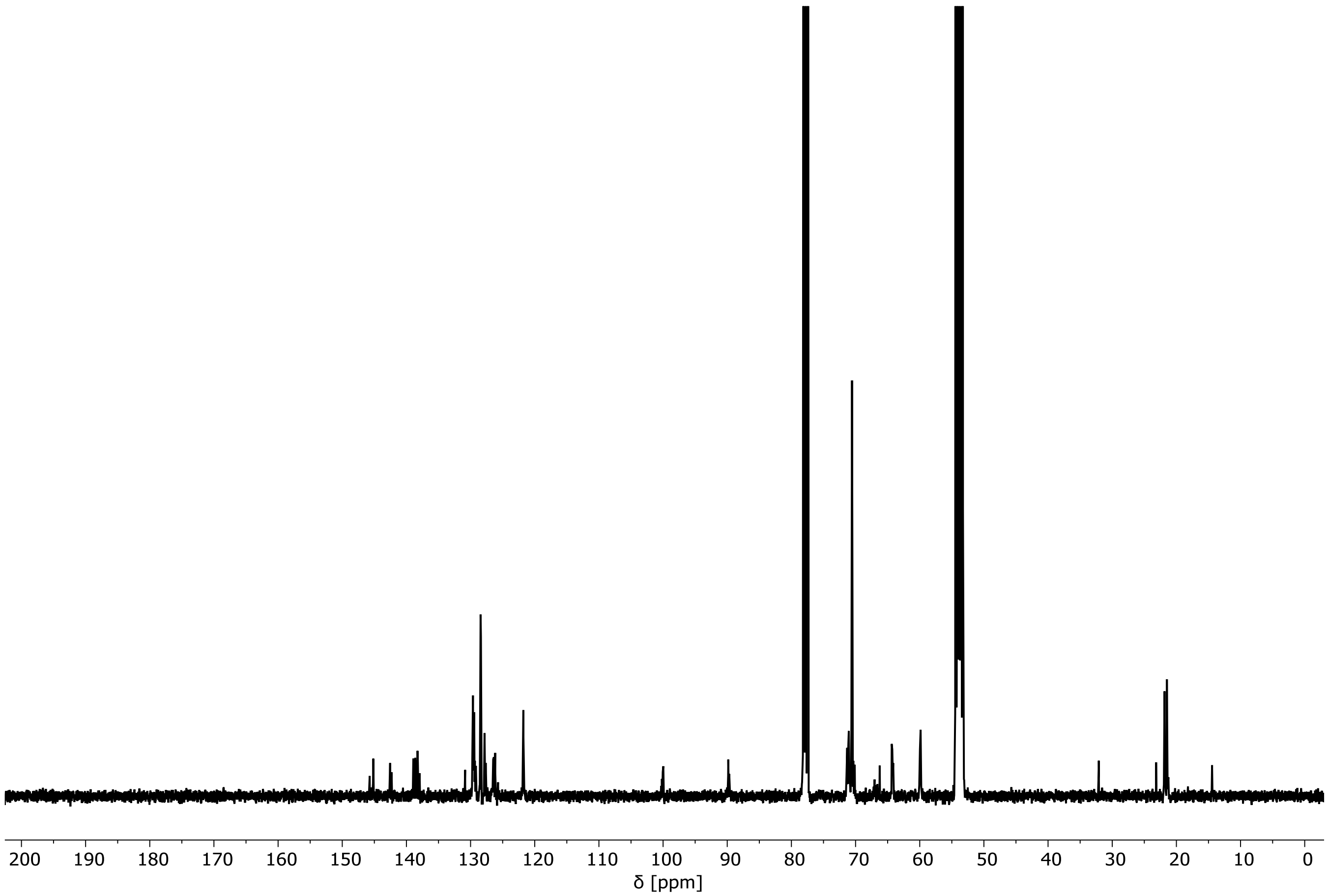

Figure S63: $^{13}C\{^{1}H\}$-NMR (101 MHz, CD_2Cl_2) spectrum of **2-2**.

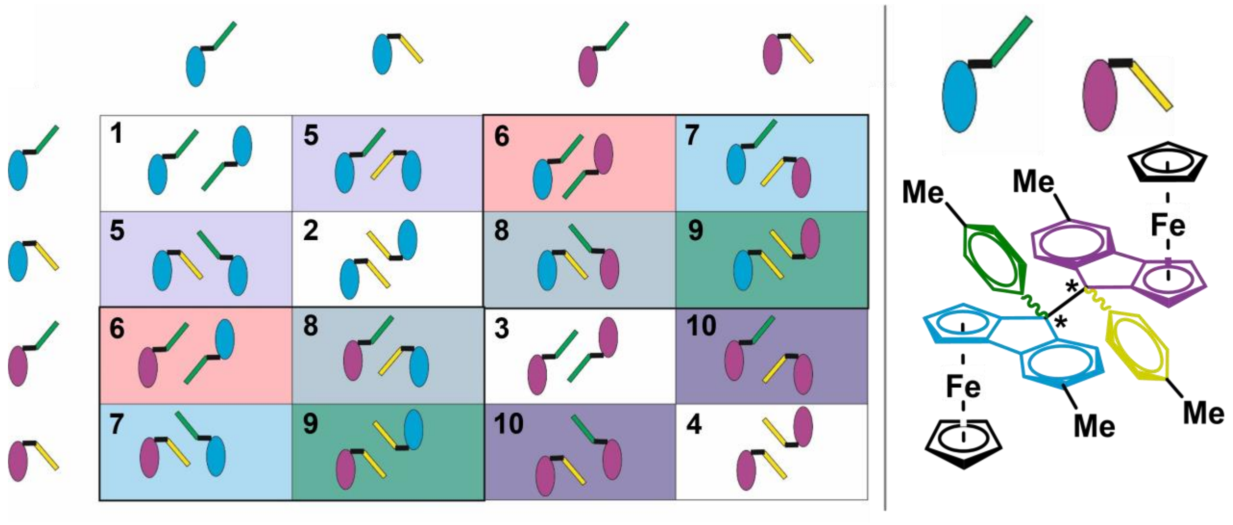

Figure S64: The ten dimers of **2-2** that can result from the combination of *Rp* and *Sp* enantiomers and *exo* or *endo* orientations of the 9-phenyl substituent. Structurally identical pairs within the sixteen possible combinations are denoted by the same color and numbering (*Sp*: blue, *Rp*: purple bulbs; *endo*: yellow, *exo*: green bars).

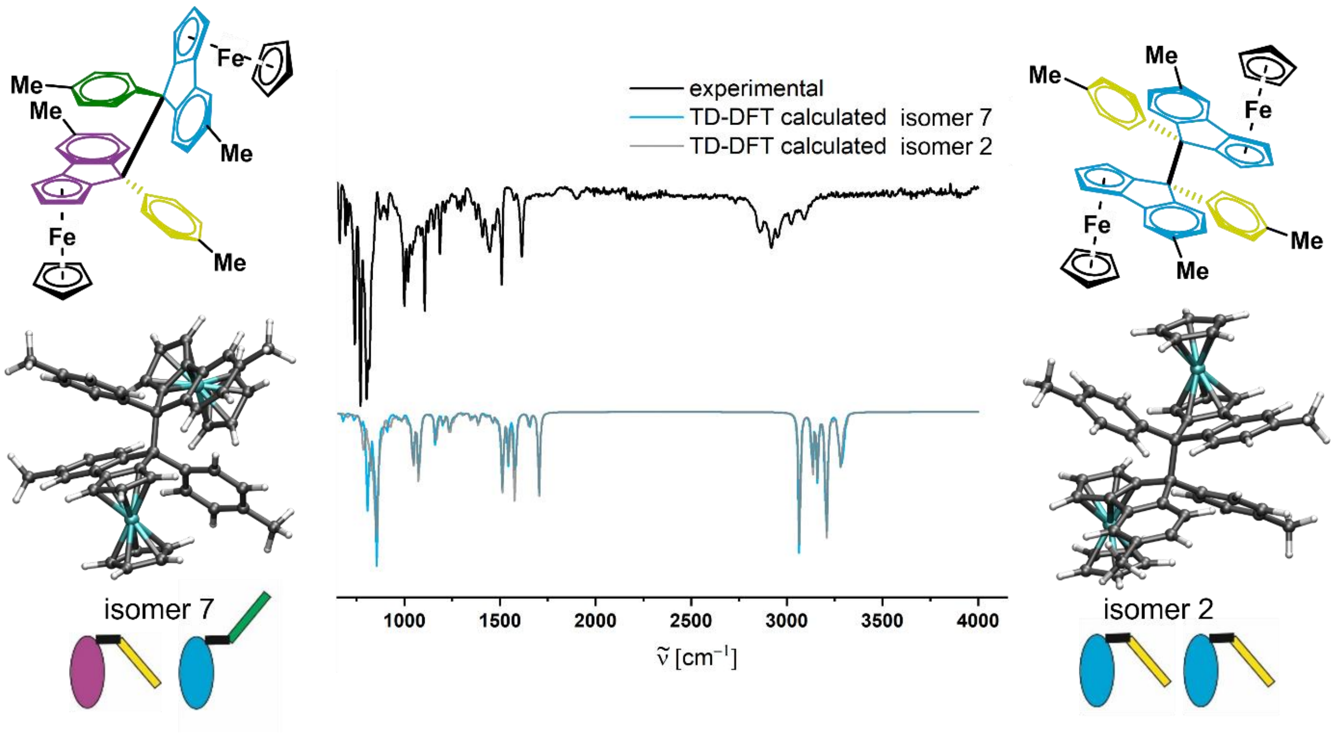

Figure S65: DFT-optimized structures of isomers **7** (*Rp*/*exo*/*Sp*/*endo*; left) and **2** (*Sp*/*exo*/*Sp*/*exo*; right) of **2**-**2** shown in Figure S14. Middle: Experimental (top, black) and overlaid DFT-calculated IR spectra of isomers 2 and 7 (blue and grey, bottom) of the mixture of isomers of dimer **2-2**.

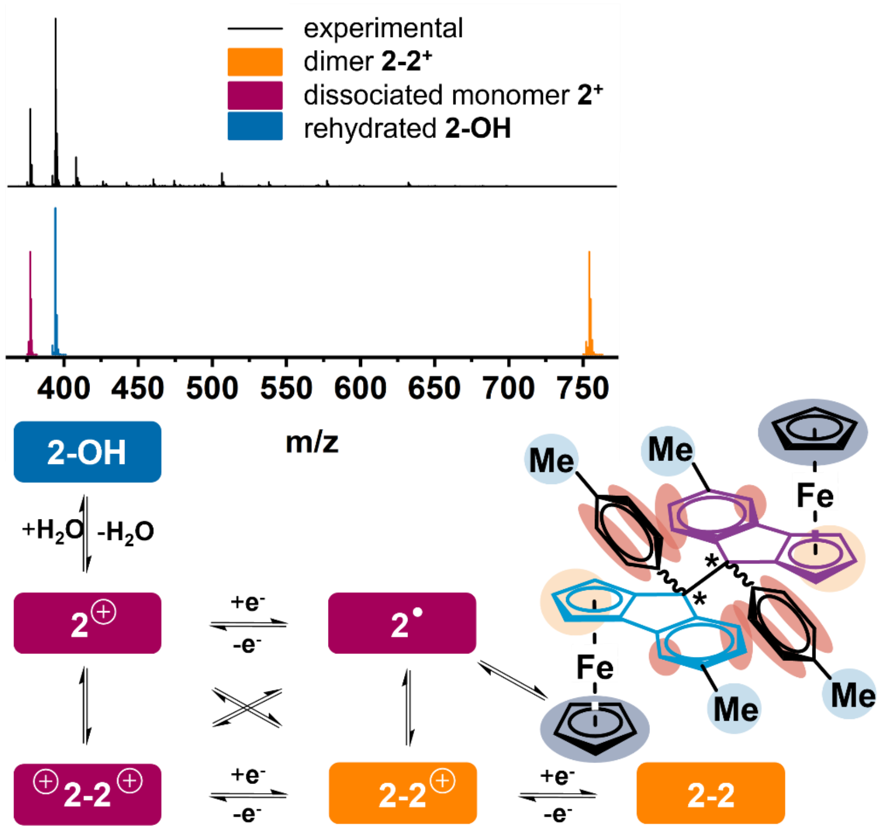

Figure S66: Experimental and simulated ESI-mass spectra of **2-2** (top). The bottom scheme shows dissociation following ionization and consecutive hydration.

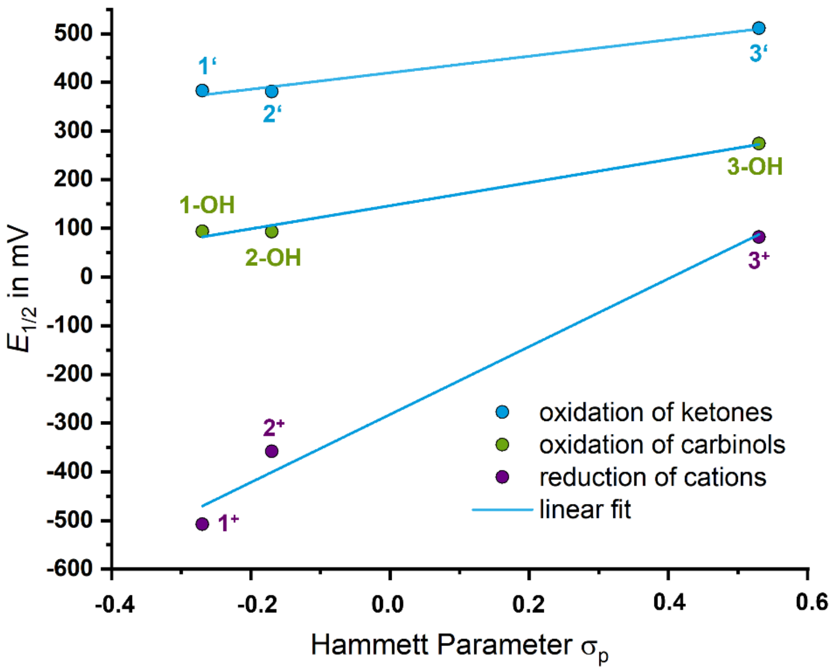

Figure S67: Hammett plot for the oxidation and reduction potentials of the ketones, carbinols and cations, respectively.

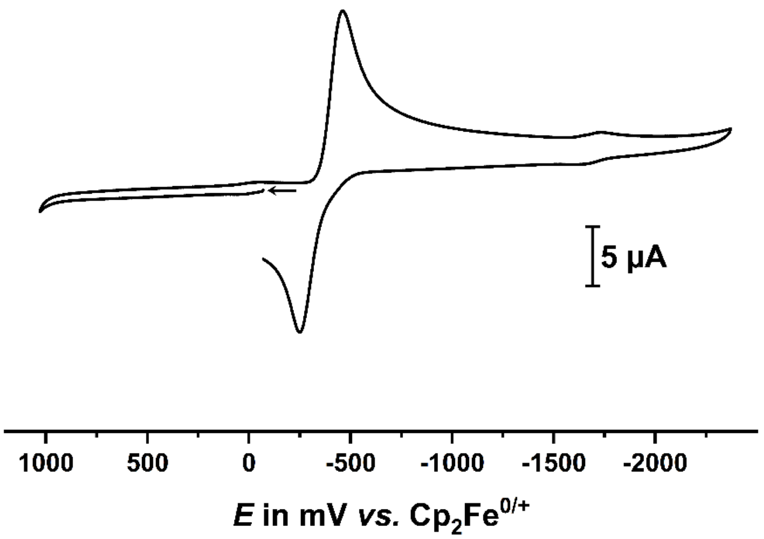

Figure S68: Cyclic voltammogram of **2**$^+$ in CH_2Cl_2/ 0.1 M NBu_4 BAr^{F24-}, T = 293(±3) K, v = 600 mV/s (Pt working, Pt counter and Ag/AgCl (pseudo) reference electrode). Plotting convention: Polarographic.

Table S3: Output parameters and estimated errors for the digital simulation of the cyclic voltammograms of compound **1**$^+$, its one-electron reduced form and associated dimers.

	E_0 [V]		α	k_s [cm^{-1}]
**1**$^+$/**1**$^•$	−0.232	± 0.05	0.5	0.01
**1-1**/[**1-1**]$^+$	0.080	± 0.01	0.5	0.01
[**1-1**]$^+$/[**1-1**]$^{2+}$	0.100	± 0.05	0.5	0.01

	K_{Diss}	$k_{b,diss}$	$k_{f,diss}$
2 **1**$^•$ ⇄ **1-1**	$2.47\cdot10^{-5} \pm 2\cdot10^{-6}$	$1.69\cdot10^{4} \pm 1\cdot10^{3}$	$4\cdot10^{-1} \pm 3\cdot10^{-1}$
**1**$^+$ + **1**$^•$ ⇄ [**1-1**]$^+$	4.76 ± 2	62.8 ± 20	$3.0\cdot10^{2} \pm 5\cdot10^{1}$
2 **1**$^+$ ⇄ [**1-1**]$^{2+}$	$1.9\cdot10^{6} \pm 0.5\cdot10^{6}$	$4.14\cdot10^{3} \pm 1\cdot10^{3}$	$8.0\cdot10^{9} \pm 1\cdot10^{10}$

	D [cm^2 s^{-1}]	
**1-1**, [**1-1**]$^+$, [**1-1**]$^{2+}$	$4.85\cdot10^{-7}$	$\pm 2\cdot10^{-7}$
**1**$^+$, **1**$^•$	$1.20\cdot10^{-6}$	$\pm 0.2\cdot10^{-6}$

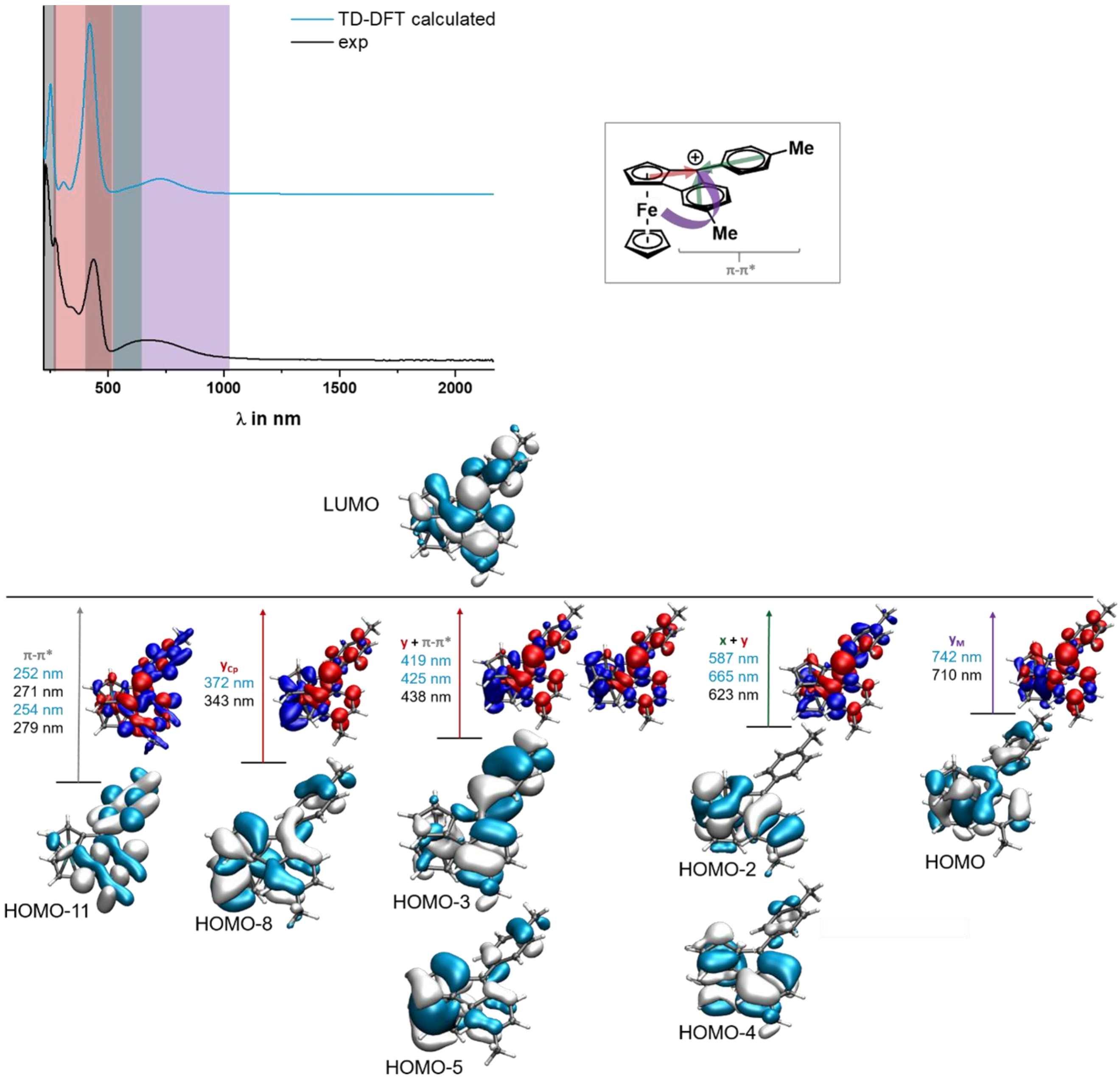

Figure S69: TD-DFT calculated UV/Vis/NIR spectrum (blue, top) and the comparison with the experimental spectrum of **2**$^{+}$ (black, bottom). The bottom part shows the MOs that are involved in important transitions. EDDM (electron density difference map) plots are displayed in dark blue (electron density loss) and red (electron density gain).

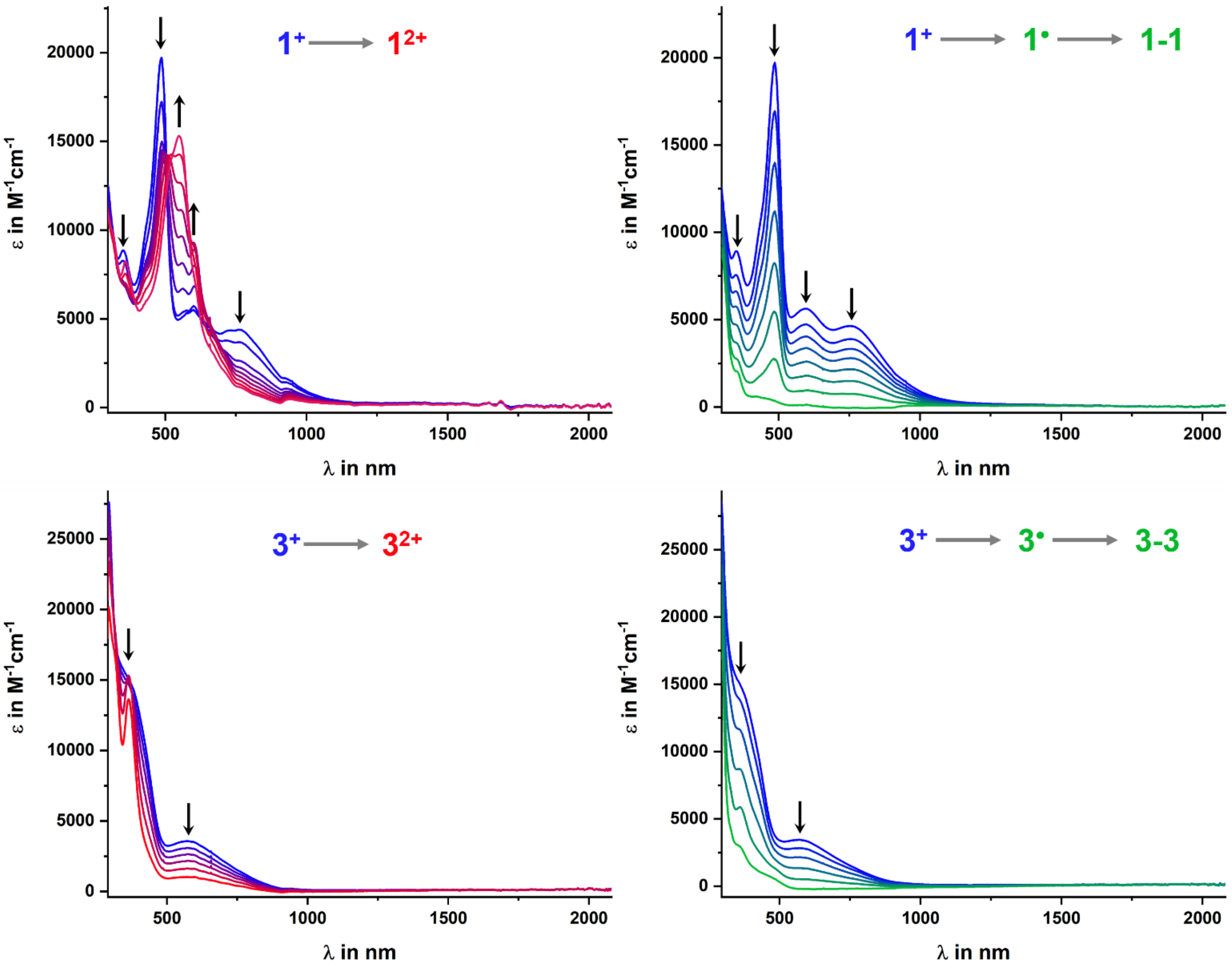

Figure S70: Spectroelectrochemistry of complexes **1**$^{+}$ and **3**$^{+}$ (1,2-$C_2H_4Cl_2$ with 0.1 M NBu_4^+ BAr^{F24-} electrolyte at T = 293(±3) K). Left: Spectral changes during oxidation are shown from blue to red colors. Right: Spectral changes during reduction are shown from blue to green colors.

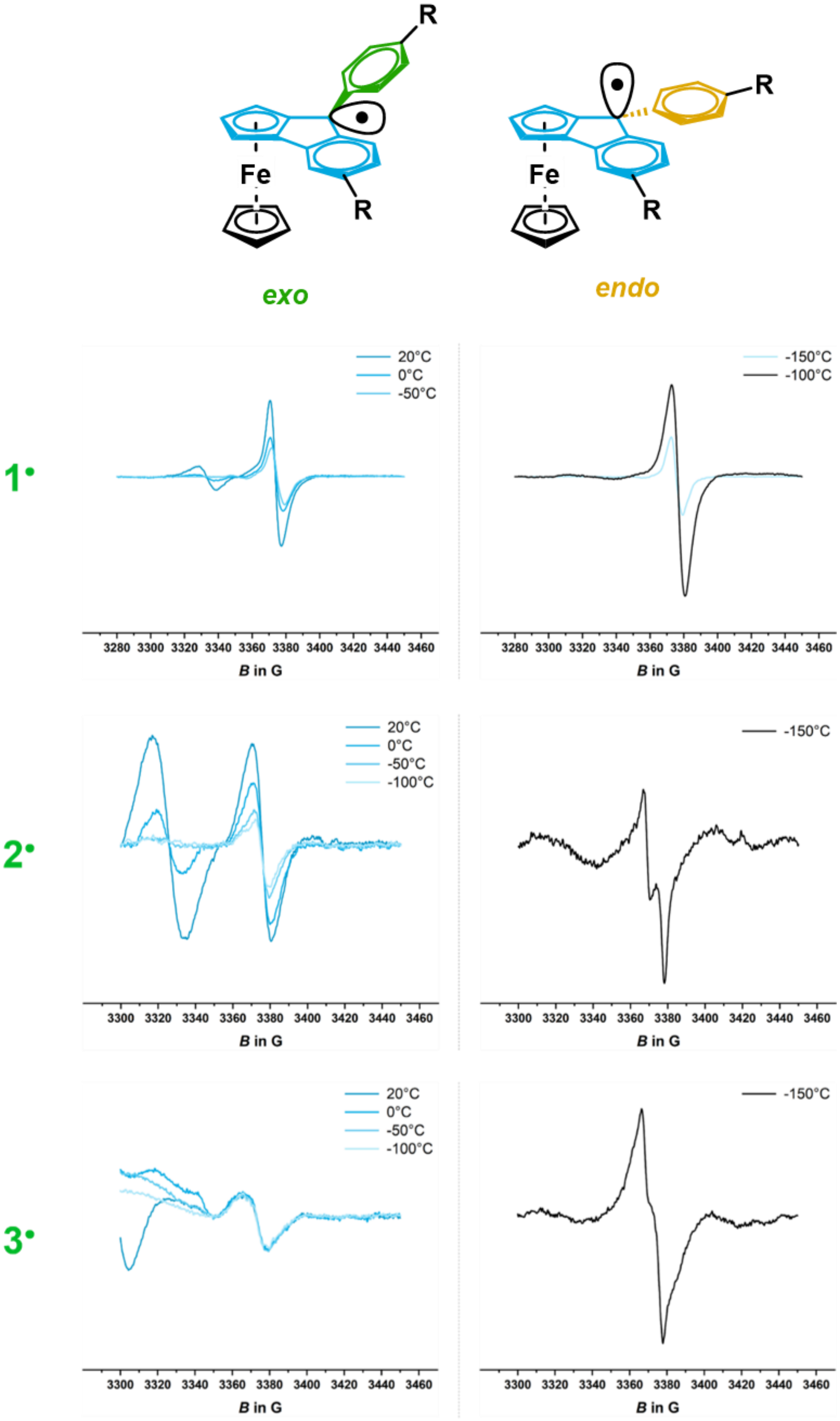

Figure S71: *T*-dependent EPR spectra of chemically generated **1•** – **3•**, with the signals between 20 °C and −100 °C on the left and the spectrum recorded in the frozen solvent −150 °C shown on the right. The y-axis scaling is identical for all spectra. The color coding denoting the stereochemistry at the chiral centers matches with that of Figures S15, S63 and S64.

Table S4: *g*-values of the minor/major components (cf. Figure S20) of the EPR signals at 20 °C and −150 °C.

	**1•**	**2•**	**3•**
**20 °C**	2.0299/2.0054	2.0342/2.0042	-/2.0056
**−150 °C**	-/2.0051	-/2.0065	-/2.0056

Table S5: DFT/pbe1pbe/6-31G(d)/pcm(/CH_2Cl_2)-calculated energies and xyz-coordinates of **1⁺**.

Sum of electronic and thermal Enthalpies: -2377.321496
Sum of electronic and thermal Free Energies: -2377.397880

Fe	1.05232	-2.01924	0.05656
C	0.06704	-0.58174	1.09169
C	1.48955	-0.37191	1.15771
C	2.08460	-1.50166	1.76765
C	1.02798	-2.41872	2.07043
C	-0.21812	-1.86935	1.65490
C	0.48664	-1.96043	-1.92330
C	0.14531	-3.21749	-1.34141
C	1.34655	-3.82360	-0.87432
C	2.42487	-2.93790	-1.15423
C	1.89208	-1.78513	-1.80548
H	3.13753	-1.66866	1.95336
H	1.16061	-3.39864	2.51104
H	-1.18161	-2.34859	1.76347
H	-0.21033	-1.24319	-2.33914
H	-0.85161	-3.63049	-1.25486
H	1.42115	-4.77128	-0.35640
H	3.46291	-3.09830	-0.89230
H	2.45346	-0.91392	-2.11904
C	-0.55271	0.58602	0.50016
C	0.51230	1.50750	0.20148
C	1.77027	0.94655	0.59174
C	-1.95630	0.77116	0.30959
C	2.95878	1.61026	0.38479
C	2.91097	2.87054	-0.24796
C	1.68499	3.41227	-0.69141
C	0.50158	2.73736	-0.48434
C	-2.55925	2.05422	0.32628
C	-2.79689	-0.34952	0.12307
C	-3.91546	2.20041	0.16018
C	-4.15568	-0.21198	-0.07567
C	-4.73094	1.07131	-0.05444
O	-6.02908	1.31857	-0.21673
O	3.98448	3.61828	-0.49122
H	3.90028	1.16904	0.69027
H	1.70585	4.36527	-1.20883
H	-0.41900	3.15757	-0.87351
H	-1.96017	2.93078	0.54545
H	-4.38588	3.17699	0.20645
H	-2.35887	-1.34135	0.07710
H	-4.76270	-1.09262	-0.24909
C	5.26735	3.14657	-0.09314
C	-6.92218	0.22715	-0.40811
H	5.96881	3.92708	-0.38513
H	5.51813	2.21426	-0.60935
H	5.30979	2.99934	0.99074
H	-7.91274	0.67158	-0.49684
H	-6.89839	-0.45185	0.45024
H	-6.68063	-0.31906	-1.32568

Table S6: DFT/pbe1pbe/6-31G(d)/pcm(/CH_2Cl_2)-calculated energies and xyz-coordinates of **1•**.

Sum of electronic and thermal Enthalpies: -2377.480604
Sum of electronic and thermal Free Energies: -2377.558818

Fe	1.08235	-2.05793	0.07717
C	0.07938	-0.54487	1.09461
C	1.50486	-0.33789	1.12858
C	2.11835	-1.46419	1.74307
C	1.06863	-2.37389	2.08824
C	-0.18579	-1.82921	1.66916
C	0.44145	-2.05086	-1.88170
C	0.15116	-3.30443	-1.27121
C	1.38294	-3.87650	-0.83482
C	2.43278	-2.97726	-1.17803
C	1.84927	-1.84583	-1.82006
H	3.17518	-1.62380	1.91642
H	1.20607	-3.34163	2.55547
H	-1.14719	-2.31140	1.78983
H	-0.28513	-1.35376	-2.28069
H	-0.83275	-3.73743	-1.14077
H	1.49698	-4.81877	-0.31357
H	3.48505	-3.11533	-0.96326
H	2.37915	-0.96789	-2.16849
C	-0.55897	0.61476	0.51871
C	0.51206	1.53590	0.19564
C	1.77572	0.97270	0.55899
C	-1.98145	0.78700	0.31972
C	2.97177	1.64735	0.34414
C	2.92743	2.90571	-0.26614
C	1.69734	3.45589	-0.67123
C	0.50709	2.78241	-0.45465
C	-2.60610	2.05335	0.36061
C	-2.81290	-0.32659	0.09749
C	-3.96952	2.19203	0.17821
C	-4.18419	-0.19906	-0.09131
C	-4.77505	1.06825	-0.05470
O	-6.09599	1.30939	-0.22375
O	4.01829	3.66785	-0.52316
H	3.91269	1.19476	0.63827
H	1.70781	4.42320	-1.16481
H	-0.41904	3.22549	-0.80704
H	-2.01627	2.93630	0.58474
H	-4.44186	3.16897	0.22587
H	-2.36730	-1.31566	0.03850
H	-4.77892	-1.08779	-0.27275
C	5.28262	3.16132	-0.14869
C	-6.94650	0.20213	-0.45116
H	6.00961	3.92225	-0.43598
H	5.51169	2.22526	-0.67320
H	5.34276	2.99271	0.93368
H	-7.95205	0.61178	-0.55491
H	-6.92535	-0.49884	0.39224
H	-6.67483	-0.32930	-1.37142

Table S7: DFT/pbe1pbe/6-31G(d)/pcm(/CH_2Cl_2)-calculated energies and xyz-coordinates of **1**-**1**.

Sum of electronic and thermal Enthalpies:	-4754.971778
Sum of electronic and thermal Free Energies:	-4755.099951

Fe	3.52489	-1.08499	-0.80651
C	1.74801	0.00855	-0.84730
C	2.77335	0.70637	-0.14278
C	3.88290	0.91861	-1.00494
C	3.54153	0.32861	-2.26067
C	2.23914	-0.26097	-2.15807
C	3.18623	-3.07136	-0.39449
C	4.11988	-2.93906	-1.46144
C	5.23733	-2.19722	-0.98105
C	4.99535	-1.87381	0.38625
C	3.72773	-2.41287	0.74686
H	4.82128	1.40055	-0.76132
H	4.18167	0.28594	-3.13364
H	1.77024	-0.84477	-2.93854
H	2.21697	-3.54989	-0.45007
H	3.98842	-3.30689	-2.47167
H	6.10286	-1.90190	-1.56128
H	5.64535	-1.29030	1.02662
H	3.23922	-2.30225	1.70668
C	0.49199	-0.14955	0.02512
C	1.06796	0.33145	1.37449
C	2.35763	0.88521	1.24074
C	0.04734	-1.62121	0.04788
C	3.03088	1.43555	2.32381
C	2.43857	1.42613	3.59226
C	1.18019	0.83420	3.73353
C	0.49899	0.28982	2.64005
C	-0.37346	-2.27388	-1.12032
C	0.18708	-2.42516	1.18606
C	-0.66723	-3.63432	-1.13884
C	-0.10619	-3.78746	1.16856
C	-0.54952	-4.42413	0.00863
C	-0.89975	-5.88539	-0.00367
C	3.14238	2.04151	4.77023
H	4.02182	1.86453	2.19028
H	0.72019	0.79242	4.71842
H	-0.47533	-0.16277	2.79237
H	-0.46968	-1.72431	-2.04671
H	-0.98816	-4.09100	-2.07309
H	0.56084	-2.00020	2.10908
H	0.02295	-4.36550	2.08151
H	3.04321	3.13451	4.76101
H	2.72771	1.68193	5.71699
H	4.21424	1.81611	4.75852
H	-0.32983	-6.43970	0.74891
H	-1.96434	-6.03632	0.21737
H	-0.70498	-6.33578	-0.98247
H	2.47894	6.50718	-0.03419
H	0.99076	4.97316	1.36463
C	1.82729	6.28714	-0.88659
H	1.05073	7.06278	-0.90770
H	2.41652	6.39054	-1.80336
H	-1.37684	-0.05735	-5.09258

C	0.83143	4.39112	0.45891
C	1.22080	4.91635	-0.77536
H	-3.59702	0.38466	-6.08962
H	0.14378	0.21047	-3.21411
C	-1.78865	0.17733	-4.11352
H	-0.03495	2.78265	1.54518
C	0.24042	3.13645	0.56004
C	-0.91686	0.32206	-3.03283
C	-4.09642	0.15543	-5.14284
C	-3.16999	0.34118	-3.97349
C	0.99641	4.12315	-1.90036
H	-4.98085	0.79515	-5.05773
H	-4.45034	-0.88184	-5.20377
H	1.28463	4.49140	-2.88305
C	0.00909	2.33352	-0.56634
C	-1.40172	0.62076	-1.76132
C	0.40319	2.86697	-1.79855
H	-2.51558	-2.54055	0.51038
C	-3.66334	0.67197	-2.70966
C	-0.65128	0.92724	-0.43388
C	-2.79215	0.80403	-1.63303
H	-4.99131	-1.74245	-0.18614
C	-3.22860	-2.04876	1.15682
H	0.24301	2.31007	-2.71271
C	-4.53764	-1.63019	0.79107
H	-4.72989	0.83258	-2.56599
C	-1.86724	1.11904	0.50603
C	-3.06972	1.18565	-0.26238
C	-3.00797	-1.66969	2.51229
Fe	-3.42739	-0.00575	1.36904
H	-2.10216	-1.84308	3.07875
C	-5.12881	-0.99245	1.92052
C	-2.19717	1.59507	1.81284
C	-4.18286	-1.01757	2.98532
C	-4.12677	1.71190	0.52648
H	-1.55015	1.67949	2.67320
H	-6.10935	-0.53358	1.95222
C	-3.57526	1.97121	1.81598
H	-5.15516	1.86502	0.22447
H	-4.32208	-0.58684	3.96922
H	-4.11923	2.34407	2.67572

Table S8: DFT/pbe1pbe/6-31G(d)/pcm(/CH_2Cl_2)-calculated energies and xyz-coordinates of **2**$^+$.

Sum of electronic and thermal Enthalpies:	-2227.065606
Sum of electronic and thermal Free Energies:	-2227.139764

Fe	1.40752	-1.57949	0.05102
C	0.10600	-0.44271	1.11498
C	1.43848	0.10867	1.18227
C	2.28774	-0.85059	1.77652
C	1.48829	-2.00602	2.06034
C	0.14804	-1.77884	1.64637
C	0.88828	-1.63896	-1.94692
C	0.84035	-2.94511	-1.37470
C	2.14072	-3.25269	-0.88211
C	2.98331	-2.13384	-1.13277
C	2.20731	-1.13474	-1.79404
H	3.35046	-0.76117	1.96033
H	1.86053	-2.92932	2.48560
H	-0.67010	-2.47959	1.74295
H	0.05024	-1.10350	-2.37627
H	-0.03162	-3.58353	-1.31403
H	2.42658	-4.15858	-0.36299
H	4.02282	-2.04490	-0.84421
H	2.55297	-0.15297	-2.09203
C	-0.76645	0.51316	0.49783
C	0.04066	1.68257	0.20340
C	1.39048	1.45851	0.61752
C	-2.17374	0.34231	0.26905
C	2.36197	2.41179	0.41268
C	2.02071	3.62282	-0.22606
C	0.70814	3.81864	-0.67590
C	-0.27958	2.85850	-0.48313
C	-3.07027	1.43004	0.32777
C	-2.69735	-0.94462	0.01429
C	-4.42657	1.23261	0.13450
C	-4.04909	-1.12294	-0.20692
C	-4.94124	-0.03963	-0.15069
C	-6.40256	-0.24343	-0.40486
C	3.06497	4.67765	-0.42276
H	3.38679	2.23871	0.72957
H	0.46077	4.73845	-1.19744
H	-1.27363	3.02683	-0.88276
H	-2.70880	2.41603	0.59873
H	-5.10481	2.07754	0.21417
H	-2.02272	-1.79200	-0.06005
H	-4.42976	-2.11661	-0.42705
H	3.33578	5.12916	0.54011
H	2.71551	5.47506	-1.08319
H	3.98216	4.25037	-0.84275
H	-6.75561	-1.18196	0.03398
H	-6.59447	-0.29984	-1.48429
H	-7.00030	0.57983	-0.00484

Table S9: DFT/pbe1pbe/6-31G(d)/pcm(/CH_2Cl_2)-calculated energies and xyz-coordinates of **2**$^{\bullet}$.

Sum of electronic and thermal Enthalpies:	-2227.224912
Sum of electronic and thermal Free Energies:	-2227.300807

Fe	1.47606	-1.60610	0.07425
C	0.10969	-0.41708	1.09382
C	1.43349	0.14837	1.14784
C	2.30645	-0.79030	1.76029
C	1.52290	-1.94420	2.08105
C	0.17640	-1.73421	1.65075
C	0.87370	-1.72582	-1.89330
C	0.92706	-3.02524	-1.31406
C	2.26304	-3.25494	-0.87158
C	3.03322	-2.09816	-1.17936
C	2.17209	-1.15031	-1.80536
H	3.36724	-0.67738	1.94475
H	1.89927	-2.84963	2.54135
H	-0.63314	-2.44431	1.75661
H	-0.01087	-1.23799	-2.28370
H	0.09646	-3.71195	-1.21017
H	2.62267	-4.14462	-0.37029
H	4.08144	-1.95244	-0.95132
H	2.44516	-0.15080	-2.11993
C	-0.79454	0.53765	0.50386
C	0.00702	1.70614	0.19415
C	1.36728	1.48720	0.58022
C	-2.20963	0.33843	0.27541
C	2.33653	2.45536	0.37021
C	1.99625	3.66705	-0.24424
C	0.67154	3.86130	-0.66498
C	-0.31378	2.90221	-0.46245
C	-3.13681	1.39708	0.32511
C	-2.71674	-0.95044	0.01701
C	-4.49009	1.17498	0.11369
C	-4.07036	-1.16096	-0.19794
C	-4.98652	-0.10376	-0.16020
C	-6.44789	-0.33132	-0.42459
C	3.02596	4.74452	-0.43878
H	3.36569	2.27590	0.67501
H	0.41182	4.78905	-1.17046
H	-1.31811	3.07997	-0.83449
H	-2.79451	2.39405	0.58412
H	-5.18217	2.01245	0.17551
H	-2.02532	-1.78655	-0.04312
H	-4.42757	-2.16796	-0.40462
H	4.03746	4.32843	-0.49218
H	3.01259	5.46153	0.39303
H	2.84413	5.31273	-1.35743
H	-6.76389	-1.32881	-0.10141
H	-6.67465	-0.25015	-1.49603
H	-7.06884	0.40597	0.09488

Table S10: DFT/pbe1pbe/6-31G(d)/pcm(/CH_2Cl_2)-calculated energies and xyz-coordinates of isomer **7** of dimer **2**-**2**.

Sum of electronic and thermal Enthalpies: -4454.465449
Sum of electronic and thermal Free Energies: -4454.588726

Fe	3.52489	-1.08499	-0.80651
C	1.74801	0.00855	-0.84730
C	2.77335	0.70637	-0.14278
C	3.88290	0.91861	-1.00494
C	3.54153	0.32861	-2.26067
C	2.23914	-0.26097	-2.15807
C	3.18623	-3.07136	-0.39449
C	4.11988	-2.93906	-1.46144
C	5.23733	-2.19722	-0.98105
C	4.99535	-1.87381	0.38625
C	3.72773	-2.41287	0.74686
H	4.82128	1.40055	-0.76132
H	4.18167	0.28594	-3.13364
H	1.77024	-0.84477	-2.93854
H	2.21697	-3.54989	-0.45007
H	3.98842	-3.30689	-2.47167
H	6.10286	-1.90190	-1.56128
H	5.64535	-1.29030	1.02662
H	3.23922	-2.30225	1.70668
C	0.49199	-0.14955	0.02512
C	1.06796	0.33145	1.37449
C	2.35763	0.88521	1.24074
C	0.04734	-1.62121	0.04788
C	3.03088	1.43555	2.32381
C	2.43857	1.42613	3.59226
C	1.18019	0.83420	3.73353
C	0.49899	0.28982	2.64005
C	-0.37346	-2.27388	-1.12032
C	0.18708	-2.42516	1.18606
C	-0.66723	-3.63432	-1.13884
C	-0.10619	-3.78746	1.16856
C	-0.54952	-4.42413	0.00863
C	-0.89975	-5.88539	-0.00367
C	3.14238	2.04151	4.77023
H	4.02182	1.86453	2.19028
H	0.72019	0.79242	4.71842
H	-0.47533	-0.16277	2.79237
H	-0.46968	-1.72431	-2.04671
H	-0.98816	-4.09100	-2.07309
H	0.56084	-2.00020	2.10908
H	0.02295	-4.36550	2.08151
H	3.04321	3.13451	4.76101
H	2.72771	1.68193	5.71699
H	4.21424	1.81611	4.75852
H	-0.32983	-6.43970	0.74891
H	-1.96434	-6.03632	0.21737
H	-0.70498	-6.33578	-0.98247
H	2.47894	6.50718	-0.03419
H	0.99076	4.97316	1.36463
C	1.82729	6.28714	-0.88659
H	1.05073	7.06278	-0.90770
H	2.41652	6.39054	-1.80336
H	-1.37684	-0.05735	-5.09258

C	0.83143	4.39112	0.45891
C	1.22080	4.91635	-0.77536
H	-3.59702	0.38466	-6.08962
H	0.14378	0.21047	-3.21411
C	-1.78865	0.17733	-4.11352
H	-0.03495	2.78265	1.54518
C	0.24042	3.13645	0.56004
C	-0.91686	0.32206	-3.03283
C	-4.09642	0.15543	-5.14284
C	-3.16999	0.34118	-3.97349
C	0.99641	4.12315	-1.90036
H	-4.98085	0.79515	-5.05773
H	-4.45034	-0.88184	-5.20377
H	1.28463	4.49140	-2.88305
C	0.00909	2.33352	-0.56634
C	-1.40172	0.62076	-1.76132
C	0.40319	2.86697	-1.79855
H	-2.51558	-2.54055	0.51038
C	-3.66334	0.67197	-2.70966
C	-0.65128	0.92724	-0.43388
C	-2.79215	0.80403	-1.63303
H	-4.99131	-1.74245	-0.18614
C	-3.22860	-2.04876	1.15682
H	0.24301	2.31007	-2.71271
C	-4.53764	-1.63019	0.79107
H	-4.72989	0.83258	-2.56599
C	-1.86724	1.11904	0.50603
C	-3.06972	1.18565	-0.26238
C	-3.00797	-1.66969	2.51229
Fe	-3.42739	-0.00575	1.36904
H	-2.10216	-1.84308	3.07875
C	-5.12881	-0.99245	1.92052
C	-2.19717	1.59507	1.81284
C	-4.18286	-1.01757	2.98532
C	-4.12677	1.71190	0.52648
H	-1.55015	1.67949	2.67320
H	-6.10935	-0.53358	1.95222
C	-3.57526	1.97121	1.81598
H	-5.15516	1.86502	0.22447
H	-4.32208	-0.58684	3.96922
H	-4.11923	2.34407	2.67572

Table S11: DFT/pbe1pbe/6-31G(d)/pcm(/CH_2Cl_2)-calculated energies and xyz-coordinates of isomer **2** of dimer **2**-**2**.

Sum of electronic and thermal Enthalpies:	-4454.475575
Sum of electronic and thermal Free Energies:	-4454.600518

Fe	3.47223	-0.19432	1.51765
C	1.57048	0.23428	0.78431
C	2.55393	1.11896	0.24960
C	3.19816	1.81700	1.30566
C	2.60686	1.35012	2.51922
C	1.61613	0.36644	2.20376
C	4.05927	-2.06199	0.89648
C	3.98443	-2.01525	2.31921
C	4.87757	-1.00254	2.77452
C	5.50277	-0.42273	1.63260
C	4.99681	-1.07799	0.47257
H	4.00066	2.53883	1.22254
H	2.88778	1.65762	3.51936
H	1.04204	-0.17686	2.94062
H	3.47397	-2.70437	0.25197
H	3.34622	-2.62773	2.94382
H	5.03217	-0.70519	3.80431
H	6.21161	0.39601	1.64306
H	5.24538	-0.83749	-0.55389
C	0.73682	-0.38907	-0.33873
C	1.58379	0.04587	-1.56105
C	2.59015	0.96915	-1.19499
C	0.52383	-1.90050	-0.22744
C	3.44265	1.54565	-2.12679
C	3.33591	1.19991	-3.47825
C	2.37230	0.25639	-3.83995
C	1.50788	-0.31780	-2.90186
C	0.77539	-2.57645	0.97272
C	0.03865	-2.67765	-1.28822
C	0.57866	-3.94780	1.10227
C	-0.15569	-4.05064	-1.16075
C	0.11631	-4.71918	0.03379
C	-0.06163	-6.20644	0.15939
C	4.23296	1.83118	-4.50671
H	4.20050	2.25649	-1.80370
H	2.28814	-0.04369	-4.88211
H	0.78450	-1.03706	-3.26117
H	1.15142	-2.02860	1.82690
H	0.79068	-4.42523	2.05717
H	-0.23239	-2.21983	-2.22984
H	-0.53477	-4.60993	-2.01382
H	3.90937	2.85407	-4.73796
H	4.22890	1.26466	-5.44295
H	5.26694	1.89477	-4.15031
H	-0.83334	-6.57653	-0.52343
H	-0.34133	-6.49139	1.17905
H	0.86820	-6.73782	-0.08206
H	0.83171	6.57699	-0.52444
H	0.53488	4.61017	-2.01322
C	0.06185	6.20647	0.16020
H	-0.86872	6.73780	-0.07854
H	0.34414	6.49102	1.17926
H	-2.28902	0.04462	-4.88170

C	0.15578	4.05081	-1.16021
C	-0.11617	4.71924	0.03439
H	-4.23011	-1.26365	-5.44227
H	-0.78528	1.03793	-3.26079
C	-2.37292	-0.25573	-3.83961
H	0.23241	2.22010	-2.22950
C	-0.03861	2.67784	-1.28783
C	-1.50843	0.31840	-2.90155
C	-4.23335	-1.83077	-4.50640
C	-3.33626	-1.19954	-3.47795
C	-0.57854	3.94777	1.10281
H	-3.90913	-2.85327	-4.73847
H	-5.26712	-1.89533	-4.14957
H	-0.79052	4.42510	2.05777
C	-0.52381	1.90061	-0.22714
C	-1.58401	-0.04563	-1.56081
C	-0.77532	2.57644	0.97311
H	-3.47388	2.70370	0.25150
C	-3.44270	-1.54559	-2.12654
C	-0.73689	0.38919	-0.33856
C	-2.59015	-0.96914	-1.19477
H	-5.24532	0.83661	-0.55380
C	-4.05918	2.06152	0.89621
H	-1.15135	2.02852	1.82724
C	-4.99676	1.07742	0.47258
H	-4.20039	-2.25662	-1.80348
C	-1.57039	-0.23431	0.78451
C	-2.55374	-1.11911	0.24981
C	-3.98433	2.01517	2.31895
Fe	-3.47217	0.19405	1.51785
H	-3.34611	2.62781	2.94339
C	-5.50271	0.42249	1.63281
C	-1.61600	-0.36651	2.20396
C	-4.87748	1.00261	2.77456
C	-3.19787	-1.81725	1.30586
H	-1.04198	0.17684	2.94084
H	-6.21156	-0.39623	1.64351
C	-2.60660	-1.35032	2.51942
H	-4.00028	-2.53919	1.22275
H	-5.03209	0.70557	3.80444
H	-2.88744	-1.65788	3.51957

Table S12: DFT/pbe1pbe/6-31G(d)/pcm(/CH_2Cl_2)-calculated energies and xyz-coordinates of **3⁺**.

Sum of electronic and thermal Enthalpies:	-2822.008152
Sum of electronic and thermal Free Energies:	-2822.093055

Fe	-0.95053	2.43779	0.01964
C	-0.12751	1.03450	1.23967
C	-1.57393	0.96948	1.27947
C	-2.05765	2.20401	1.75903
C	-0.91991	3.04676	1.99065
C	0.27225	2.35896	1.65180
C	-0.48418	2.19215	-1.98598
C	0.01952	3.43487	-1.49586
C	-1.08826	4.19638	-1.02795
C	-2.26581	3.41764	-1.20492
C	-1.89068	2.17552	-1.80053
H	-3.09084	2.49604	1.89402
H	-0.97084	4.07477	2.32589
H	1.27681	2.75208	1.72999
H	0.11232	1.37728	-2.37811
H	1.05725	3.74195	-1.47933
H	-1.03855	5.17410	-0.56621
H	-3.26661	3.70513	-0.90938
H	-2.55530	1.35392	-2.03549
C	0.37495	-0.14359	0.63464
C	-0.77611	-1.00726	0.36903
C	-1.97389	-0.34091	0.76509
C	1.76752	-0.45001	0.38567
C	-3.20350	-0.93642	0.58681
C	-3.24239	-2.20881	-0.00547
C	-2.08522	-2.85399	-0.43133
C	-0.83960	-2.24868	-0.26015
C	2.26175	-1.75575	0.55987
C	2.65638	0.57419	0.00654
C	3.60827	-2.02558	0.36446
C	3.99382	0.29535	-0.21429
C	4.46979	-1.00432	-0.03041
C	5.93778	-1.28265	-0.21004
C	-4.58901	-2.84268	-0.23141
H	-4.12029	-0.44150	0.89019
H	-2.15157	-3.82805	-0.90217
H	0.05087	-2.74520	-0.62895
H	1.60653	-2.54632	0.90793
H	3.98656	-3.03041	0.51758
H	2.28162	1.57905	-0.15959
H	4.66713	1.08334	-0.53477
F	-5.37081	-2.71816	0.85161
F	-4.49359	-4.14547	-0.52037
F	-5.23072	-2.24787	-1.25065
F	6.45552	-0.55615	-1.21063
F	6.17152	-2.57382	-0.47710
F	6.62389	-0.97227	0.90176

Table S13. DFT/pbe1pbe/6-31G(d)/pcm(/CH_2Cl_2)-calculated energies and xyz-coordinates of **3•**.

Sum of electronic and thermal Enthalpies: -2822.192023
Sum of electronic and thermal Free Energies: -2822.278800

Fe	-1.05120	2.53128	0.05094
C	-0.13573	1.01207	1.15571
C	-1.57586	0.92257	1.21705
C	-2.08741	2.12893	1.76983
C	-0.96541	2.97469	2.03742
C	0.23407	2.31819	1.62476
C	-0.48748	2.38653	-1.94013
C	-0.08469	3.64102	-1.40226
C	-1.25604	4.32032	-0.95172
C	-2.37887	3.48580	-1.21386
C	-1.90114	2.28579	-1.81830
H	-3.12507	2.38129	1.94743
H	-1.02120	3.97926	2.43860
H	1.23081	2.73358	1.69576
H	0.17194	1.61977	-2.32789
H	0.93129	4.00793	-1.32968
H	-1.28261	5.29126	-0.47330
H	-3.40969	3.70956	-0.97026
H	-2.50425	1.43314	-2.10446
C	0.39737	-0.20170	0.61634
C	-0.74301	-1.05969	0.35345
C	-1.95548	-0.38507	0.71312
C	1.80111	-0.48640	0.38693
C	-3.19079	-0.99249	0.54700
C	-3.23929	-2.27618	0.00126
C	-2.06664	-2.93347	-0.39473
C	-0.82591	-2.33296	-0.23029
C	2.33317	-1.78380	0.51789
C	2.68593	0.55512	0.04155
C	3.68206	-2.02908	0.30555
C	4.03300	0.31093	-0.17356
C	4.53730	-0.98456	-0.04536
C	6.00170	-1.23686	-0.22332
C	-4.55987	-2.93736	-0.23735
H	-4.10514	-0.48253	0.83380
H	-2.12998	-3.92516	-0.83215
H	0.06420	-2.85456	-0.56624
H	1.69142	-2.60062	0.83050
H	4.07210	-3.03589	0.41767
H	2.29700	1.56022	-0.09198
H	4.69311	1.12521	-0.45643
F	-5.52911	-2.43958	0.54960
F	-4.50660	-4.26361	-0.01662
F	-4.97863	-2.78127	-1.51057
F	6.54698	-0.41135	-1.13375
F	6.25290	-2.49577	-0.62027
F	6.68612	-1.04950	0.92302

Table S14: DFT/pbe1pbe/6-31G(d)/pcm(/CH_2Cl_2)-calculated energies and xyz-coordinates of **3**-**3**.

Sum of electronic and thermal Enthalpies: -5644.393243
Sum of electronic and thermal Free Energies: -5644.538099

Fe	2.41265	-2.49476	1.96166
C	1.31031	-0.89629	1.22837
C	2.63244	-0.91660	0.69046
C	3.58215	-0.83498	1.74332
C	2.83346	-0.76796	2.95669
C	1.43748	-0.81939	2.64737
C	1.58157	-4.27526	1.35884
C	1.55727	-4.17377	2.78050
C	2.89966	-4.02637	3.23446
C	3.75285	-4.03537	2.09326
C	2.93785	-4.18901	0.93469
H	4.66093	-0.85074	1.65716
H	3.25101	-0.72859	3.95535
H	0.65115	-0.82504	3.38838
H	0.71791	-4.36914	0.71368
H	0.67356	-4.18867	3.40615
H	3.21379	-3.89822	4.26278
H	4.82849	-3.91079	2.10270
H	3.28470	-4.19497	-0.09142
C	0.27495	-0.78734	0.10716
C	1.18519	-1.05373	-1.11802
C	2.55159	-1.06019	-0.75092
C	-0.91727	-1.73952	0.22510
C	3.56530	-1.22077	-1.68495
C	3.21804	-1.40847	-3.02191
C	1.87959	-1.44811	-3.40167
C	0.86707	-1.27477	-2.45505
C	-1.18500	-2.39372	1.43425
C	-1.80800	-1.97015	-0.83265
C	-2.27129	-3.24716	1.58277
C	-2.89515	-2.82690	-0.69821
C	-3.13126	-3.47174	0.51135
C	-4.26761	-4.43740	0.64581
C	4.29422	-1.52333	-4.05907
H	4.60623	-1.21668	-1.37719
H	1.61529	-1.61856	-4.44017
H	-0.15391	-1.31227	-2.80748
H	-0.52511	-2.25180	2.27914
H	-2.44821	-3.73329	2.53727
H	-1.69297	-1.46124	-1.77894
H	-3.56465	-2.97891	-1.53895
H	3.57000	2.97796	-1.53137
C	4.26672	4.44020	0.65233
H	-1.60984	1.62893	-4.43865
C	2.89829	2.82701	-0.69219
C	3.13095	3.47396	0.51698
H	0.15814	1.32269	-2.80416
C	-1.87503	1.45495	-3.40097
H	1.69944	1.45918	-1.77398
C	1.81188	1.96966	-0.82819
C	-0.86331	1.28124	-2.45352
C	-4.28916	1.52643	-4.06097
C	-3.21383	1.41122	-3.02297

C	2.26835	3.25072	1.58649
H	2.44250	3.73847	2.54067
C	0.91847	1.74033	0.22770
C	-1.18275	1.05559	-1.11764
C	1.18283	2.39647	1.43649
H	-0.72458	4.36925	0.71457
C	-3.56242	1.21947	-1.68690
C	-0.27339	0.78807	0.10792
C	-2.54956	1.05866	-0.75200
H	-3.29097	4.19027	-0.09075
C	-1.58810	4.27332	1.35964
H	0.52081	2.25546	2.27988
C	-2.94418	4.18456	0.93539
H	-4.60373	1.21276	-1.38047
C	-1.30977	0.89497	1.22826
C	-2.63149	0.91312	0.68917
C	-1.56367	4.17115	2.78127
Fe	-2.41563	2.49088	1.96147
H	-0.68002	4.18763	3.40697
C	-3.75892	4.02867	2.09384
C	-1.43805	0.81697	2.64710
C	-2.90578	4.02079	3.23508
C	-3.58196	0.82916	1.74115
H	-0.65233	0.82391	3.38875
H	-4.83431	3.90187	2.10316
C	-2.83421	0.76278	2.95516
H	-4.66068	0.84309	1.65406
H	-3.21967	3.89146	4.26332
H	-3.25257	0.72211	3.95342
F	-5.29119	-4.12885	-0.16826
F	-4.74875	-4.47615	1.90019
F	-3.89354	-5.69467	0.33735
F	-4.65299	0.31799	-4.53295
F	3.90079	-2.24927	-5.11821
F	5.40602	-2.09669	-3.56855
F	4.65840	-0.31474	-4.53040
F	5.29409	4.12773	-0.15540
F	4.74193	4.48492	1.90876
F	3.89426	5.69602	0.33598
F	-3.89505	2.25279	-5.11955
F	-5.40140	2.09955	-3.57105

References

(1) Panetta, C. A.; Garlick, S. M.; Durst, H. D.; Longo, F. R.; Ward, J. R. ffu:Potent catalysts for the hydrolysis of phosphorus esters. *J. Org. Chem.* **1990**, *55* (18), 5202–5205. DOI: 10.1021/jo00305a011.

(2) Deng, R.; Huang, Y.; Ma, X.; Li, G.; Zhu, R.; Wang, B.; Kang, Y.-B.; Gu, Z. Palladium-catalyzed intramolecular asymmetric C-H functionalization/cyclization reaction of metallocenes:An efficient approach toward the synthesis of planar chiral metallocene compounds. *J. Am. Chem. Soc.* **2014**, *136* (12), 4472–4475. DOI: 10.1021/ja500699x.

(3) Banide, E. V.; Ortin, Y.; Chamiot, B.; Cassidy, A.; Niehaus, J.; Moore, A.; Seward, C. M.; Müller-Bunz, H.; McGlinchey, M. J. Syntheses, Structures, and Dimerizations of Ferrocenyl- and Fluorenylideneallenes: Push−Pull Multiple Bonds? *Organometallics* **2008**, *27* (16), 4173–4182. DOI: 10.1021/om800293m.

(4) Nottingham, C.; Müller-Bunz, H.; Guiry, P. J. A Family of Chiral Ferrocenyl Diols: Modular Synthesis, Solid-State Characterization, and Application in Asymmetric Organocatalysis. *Angew. Chem. Int. Ed.* **2016**, *55* (37), 11115–11119. DOI: 10.1002/anie.201604840.
